# Supplementary material for: Why India is struggling to feed their young children? A qualitative analysis for tribal communities
Source: BMJ Open. 2022 Jul 27;12(7):e051558. doi: 10.1136/bmjopen-2021-051558 (PMC9341212; doi:10.1136/bmjopen-2021-051558)
Supplement: Supplementary data [file bmjopen-2021-051558supp001.pdf]

Supplementary Table

Trends in IYCF indicators in India (in %)

	States	Children receiving solid or semi-solid food and breast milk (%)		Trends in IYCF (%)
		NFHS 3 (2005 – 2006)	NFHS 4 (2015 – 2016)	
**India**		52.6	42.7	-9.9
**North**	Rajasthan	38.7	30.1	-8.6
	Punjab	50.9	41.1	-9.8
	Uttar Pradesh	41.2	32.6	-8.6
**North East**	Sikkim	85.4	61.8	-23.6
	Manipur	77.4	78.38	1.4
	Arunachal Pradesh	80.2	53.6	-26.6
**West**	Gujarat	54.1	49.4	-4.7
	Maharashtra	45.5	43.3	-2.2
**Central**	Madhya Pradesh	46	38.1	-7.9
	Chhattisgarh	49	53.8	4.8
**East**	Bihar	54.5	30.7	-23.8
	Jharkhand	60.2	47.2	-13
	West Bengal	47.1	52	4.9
	Odisha	65.4	54.9	-10.5
**South**	Karnataka	69.7	46	-23.7
	Tamil Nadu	81.2	67.5	-13.7
	Kerala	93.9	63.1	-30.8

Source: Dasgupta, Chaand and Rakshit (2018) [19]
