# Supplementary material for: Why India is struggling to feed their young children? A qualitative analysis for tribal communities
Source: BMJ Open. 2022 Jul 27;12(7):e051558. doi: 10.1136/bmjopen-2021-051558 (PMC9341212; doi:10.1136/bmjopen-2021-051558)
Supplement: Supplementary data [file bmjopen-2021-051558supp002.pdf]

Core Theme	Sub-theme	Participant type	FDG/KII	Example Quote
**Infant and Young Child Feeding (IYCF) – Current Practices**	*Partial breastfeeding in the first 6 months*	Mothers, Kushalgarh	FDG	"*When the child starts crying after birth then breast milk is produced and the mother is ready to breastfeed the child*."
		Mothers, Ghatol	FDG	"*No, we do not breastfeed the child soon after childbirth. Breastfeeding depends on the mother's ability to produce milk. Breastfeeding begins only after midnight if a birth occurs during the evening.*
		AS, Kushalgarh	KII	"*If the baby is delivered at home breastfeeding is starts immediately after birth. Most cases I am present during the childbirth and hence I ensure that breastfeeding starts within an hour for cases that I attend.*"
		Grandmothers, Kushalgarh	FGD	"*Only when the mother's milk secretion does not start, goat's milk is given. Honey or any water is not given*."
		Mothers, Ghatol	FGD	"*Mothers of children who are on breast milk work inside the village, so they can be summoned home whenever the child cries"*
		AWW, Ghatol	KII	"*In the summer season the child becomes thirsty, so water is given to the child. We also recommend giving water sometimes*."
		Grandmothers, Kushalgarh	FDG	"*When the child's mother is out of the village due to some work or is working in the field, goat milk is given when the child cries*."
	Biscuits as complementary food after 6 months	Grandmothers, Kushalgarh	FGD	"*Children like biscuits and it is easy to feed them. We give them one biscuit and they keep eating it. We do not have to feed them*."
		Mothers, Ghatol	FGD	"*This is the cheapest option and is easily available in the village*."
		ANM, Kushalgarh	KII	"*We tell the mothers that they can give biscuits to the child, at least it will keep them full*."
		Mothers, Ghatol	FDG	"*The baby starts drinking milk (dairy) after 6 months. We also give rab (grounded maize porridge), biscuits dipped in the water and sometime daal ka pani (lentil soup). We know what to give and what not to. The AWW also tells us about this. We only take care of all things related to the child like cooking and feeding*."

	Dietary preferences and recent shifts	Mothers FGD, Kushalgarh	FGD	"*We feed them separately. We do not feed them with us. Whenever the children get hungry we feed them. They do not understand anything; they eat whatever we give them.*"
		Ward Panch, Ghatol	KII	"*Nobody eats non-vegetarian food as they have become 'followers'. This is a recent trend. Hawan (worshipping with holy fire) was conducted in the village after which every family has stopped eating. No question of giving it to children.*"
		Teacher, Kushalgarh	KII	"*Very few families eat non-vegetarian (foods). Those who eat also give their children. This is made only if there is a guest at home or there is any occasion. Chicken is mostly eaten (on such occasions). Eggs are not eaten as they are left to hatch and the goats are sold.*"
		Teacher, Kushalgarh	KII	"*Fruits are not very common in daily diet. Only when the parents go to the market they get fruits. There is availability of local fruits which are not too good for children, like tamarind and plum.*"
		ANM, Ghatol	KII	"*Some vegetables are grown in the backyard of every home, mostly beans and brinjal. This is used quite regularly. Potatoes, tomatoes and onion are cooked almost daily. The local vegetables like spinach are seldom grown as people do not want to eat them.*"
	Time Constraints	Grandmother, Kushalgarh	FDG	"*The mother cannot sit at home taking care of the child. Who will look after the animals, get water or cook? They also have to do agricultural work.*"
		ANM, Ghatol	KII	"*Some mothers work a lot and hence are not able to take care of the child adequately. Grandmothers help, but they are also old people and cannot do much.*"
		Grandmother, Ghatol	FDG	*When my daughter-in-law works outside, then we grandmothers take care of the children. Like when the child is crying and have to be fed; we keep them in our laps and make them comfortable. We keep an eye on the children and their activities.*"
		School Teacher, Kushalgarh	KII	"*Most people here are in agriculture. Both males and females of all families take part in agriculture. Men play an important role of sowing and harvesting while women do the rest. Women are engaged all year round.*"
		Ward Panch, Ghatol	KII	"*Most houses keep goats, some also have cows. Buffaloes are very less in the village. All of these animals do not give milk. Milk from goat is used for consumption at home. Cow*

**Infant and Young Child Feeding (IYCF) – Key Drivers and Challenges**	*Livelihood challenges in a chronic poverty setting*			*and buffalo milk is used for making ghee. Few families also sell it. They do not feed milk to their children"*
		Mothers, Ghatol	FDG	*Taking care of the animals is the work of women as they stay at home. They are the ones who clean the shed and give the animals fodder and water*."
		School Teacher, Ghatol	KII	"*Some men from the village work in Mayur mills (textile mill near Banswara). Some also work as wage labour, but only during lean seasons. Women with small children do not go out of the village to work*."
	Poor Quality of Childcare services	AWW, Kushalgarh	KII	"*Neither LS (Lady Supervisor) or the CDPO (Child Development Project Officer) visits our centre. We only go to the sector meetings where they ask us about the number of pregnant women registered, children born and packets of THR distributed. They sometime give us new information. The focus is on children coming to AWC."*
		AWW, Ghatol	KII	*"We take the weight of the child who comes for the vaccination. Sometimes the mothers do not get the card when they come for vaccination, so weight cannot be recorded in the (Mamta) card."*
		AWW, Kushalgarh	KII	*"We take weight, but currently the weighing machine is not working."*
	Emerging Role of Schools and Panchayats	School Teacher, Kushalgarh	KII	"*The AWC is in the school premises; we can always keep a check whether children are getting their meals. The AWW and the other staff also come in time and regularly open the centre*."
		School Teacher, Ghatol	KII	"*We are already overburdened with the work of the school. It is not possible to check the functioning of the AWC. However sometimes while going back from school I go to the AWC and speak to the workers. I have seen their records and gave them suggestions to maintain it better. What more can we do?"*
		AWW, Ghatol	KII	"*Toilets have been constructed in every household but not everyone is using it. Elder members prefer open defecation*."
		ANM, Kushalgarh	KII	"*Few families have constructed the toilets and very few members use it due to scarcity of water*."

	Vital role of WASH practices	Ward Panch, Kushalgarh	KII	"*Those who constructed the toilets have not got their due payments. I have been following up with the Sarpanch's office regularly, but there is no response. This discourages those who have not yet constructed.*"
		Ward Panch, Ghatol	KII	"*The government has constructed toilets in every household. Whatever facilities the government has provided us, I have provided in this village. Half of the villagers use toilets. There is a delay in payment, but the Sarpanch has told me that it will be soon released.*"
