# Supplementary material for: Why India is struggling to feed their young children? A qualitative analysis for tribal communities
Source: BMJ Open. 2022 Jul 27;12(7):e051558. doi: 10.1136/bmjopen-2021-051558 (PMC9341212; doi:10.1136/bmjopen-2021-051558)
Supplement: Author's manuscript [file bmjopen-2021-051558.draft_revisions.pdf]

BMJ Open

BMJ Open is committed to open peer review. As part of this commitment we make the peer review history of every article we publish publicly available.

When an article is published we post the peer reviewers' comments and the authors' responses online. We also post the versions of the paper that were used during peer review. These are the versions that the peer review comments apply to.

The versions of the paper that follow are the versions that were submitted during the peer review process. They are not the versions of record or the final published versions. They should not be cited or distributed as the published version of this manuscript.

BMJ Open is an open access journal and the full, final, typeset and author-corrected version of record of the manuscript is available on our site with no access controls, subscription charges or pay-per-view fees (http://bmjopen.bmj.com).

If you have any questions on BMJ Open's open peer review process please email info.bmjopen@bmj.com

BMJ Open

Why India is struggling to feed their young children? A Qualitative analysis for tribal communities

Journal:	*BMJ Open*
Manuscript ID	bmjopen-2021-051558
Article Type:	Original research
Date Submitted by the Author:	06-May-2021
Complete List of Authors:	Lakhanpaul, Monica; University College London, Institute of Child Health; Whittington Health NHS Trust<br>Roy, Susrita; Save The Children, National Support Office<br>Benton, Lorna; University College London Institute of Child Health, Population, Policy and Practice<br>Lall, Marie; University Colege London, Institute of Education<br>Khanna, Rajesh; Save the Children, National Support Office<br>Vijay, Virendra ; Indian Institute of Technology Delhi<br>Sharma, Sanjay ; University College London, Engineering for International Development Centre<br>Manikam, Logan; University College London Institute of Epidemiology and Health Care, Department of Epidemiology and Public Health; Aceso Global Health Consultants<br>Santwani, Neha; Save the Children Fund, Health and Nutrition<br>Reddy, Hanimi; Save the Children, National Support Office<br>Chaturvedi, Hemant; Save the Children, National Support Office<br>Allaham, Shereen ; UCL, Department of Epidemiology and Public Health; Aceso Global Health Consultants Ltd,<br>Pattanaik, Satya Prakash ; Save the Children, National Support Office<br>Singh, Tol; Save the Children<br>Pandya, Pramod; Save the Children<br>Dang, Priyanka; Save the Children, National Support Office<br>Parikh, Priti; University Colege London, Engineering for International Development Centre, Faculty of the Built Environment, London, UK
Keywords:	NUTRITION & DIETETICS, Community child health < PAEDIATRICS, PUBLIC HEALTH

SCHOLARONE™
Manuscripts

I, the Submitting Author has the right to grant and does grant on behalf of all authors of the Work (as defined in the below author licence), an exclusive licence and/or a non-exclusive licence for contributions from authors who are: i) UK Crown employees; ii) where BMJ has agreed a CC-BY licence shall apply, and/or iii) in accordance with the terms applicable for US Federal Government officers or employees acting as part of their official duties; on a worldwide, perpetual, irrevocable, royalty-free basis to BMJ Publishing Group Ltd ("BMJ") its licensees and where the relevant Journal is co-owned by BMJ to the co-owners of the Journal, to publish the Work in this journal and any other BMJ products and to exploit all rights, as set out in our licence.

The Submitting Author accepts and understands that any supply made under these terms is made by BMJ to the Submitting Author unless you are acting as an employee on behalf of your employer or a postgraduate student of an affiliated institution which is paying any applicable article publishing charge ("APC") for Open Access articles. Where the Submitting Author wishes to make the Work available on an Open Access basis (and intends to pay the relevant APC), the terms of reuse of such Open Access shall be governed by a Creative Commons licence – details of these licences and which Creative Commons licence will apply to this Work are set out in our licence referred to above.

Other than as permitted in any relevant BMJ Author's Self Archiving Policies, I confirm this Work has not been accepted for publication elsewhere, is not being considered for publication elsewhere and does not duplicate material already published. I confirm all authors consent to publication of this Work and authorise the granting of this licence.

For peer review only

Why India is struggling to feed their young children? A Qualitative analysis for tribal communities

Professor Monica Lakhanpaul; MBBS, MD, FRCPCH [1,2]

Susrita Roy; MA, MPhil[3]

Dr Lorna Benton; PhD [1]

Professor Marie-Carine Lall; MPhil, PhD [4]

Dr. Rajesh Khanna; MBBS, DNB, MSc [3]

Professor Virendra Kumar Vijay, PhD [5]

Sanjay Sharma; MSc, LLB [6]

Dr Logan Manikam, MBcHB, FFPH, PhD [7,8]

Neha Santwani; MSc [3]

Dr Hanimi Reddy; MPhil, MSc, PhD [3]

Hemant Chaturvedi; MSc [9]

Shereen Allaham; PharmD, MPH [7,8]

Satya Prakash Pattanaik [6]

Tol Singh [9]

Pramod Pandya [9]

Priyanka Dang; MBA [3]

Dr Priti Parikh; MPhil, PhD [10]

Institutions

1. Population, Policy and Practice, UCL Great Ormond Street Institute of Child Health, 30 Gilford Street, London, WC1N 1EH, UK
2. Whittington Health NHS Trust, London, N19 5NF, UK
3. Save the Children, National Support Office, Gurugram, India
4. Institute of Education, University College London, UK
5. Indian Institute of Technology Delhi (IIT), India
6. Save the Children India, Rajasthan State Programme Office, Jaipur, India
7. Department of Epidemiology and Public Health, University College London Institute of Epidemiology and Health Care, London, UK.
8. Aceso Global Health Consultants Limited, London, UK.
9. Save the Children, Rajasthan, India
10. UCL Engineering for International Development Centre, Bartlett School of Construction and Project Management, The Bartlett, UCL Faculty of the Built Environment, London, UK

For peer review only - http://bmjopen.bmj.com/site/about/guidelines.xhtml

Corresponding author:

Monica Lakhanpaul,

Professor of Integrated Community Child Health

Population, Policy and Practice, UCL Great Ormond Street Institute of Child Health, London WC1N 1EH.

Email address: m.lakhanpaul@ucl.ac.uk

Telephone: 02079052259

Word count: 5699

Acknowledgements:

PANChSHEEEL gratefully acknowledges the input from our community champions and field team, which made this project possible. We would like to thank Isabel-Catherine Demel, a medical student who helped out in formatting and finalising the paper. The opinions in this paper solely reflect the views of the authors and not of organizations who have financially supported this research. All data used in this research is the property of the authors. The data that support the findings of this study are available from the corresponding author, Prof Monica Lakhanpaul, upon reasonable request. Prof Monica Lakhanpaul was supported by the National Institute for Health Research (NIHR) Collaboration for Leadership in Applied Health Research and Care (CLAHRC) North Thames at Bart's Health NHS Trust. This work is supported by the NIHR GOSH BRC. Dr Logan Manikam and Shereen Al Laham are funded via an NIHR Advanced Fellowship (Ref: NIHR300020).

For peer review only - http://bmjopen.bmj.com/site/about/guidelines.xhtml

Why India is struggling to feed their young children? A Qualitative analysis for tribal communities

Abstract

Objective: This interdisciplinary qualitative study aims to explore the health, education, engineering and environment (HEEE) factors impacting on feeding practices in rural India. The ultimate goal of the PANChSHEEEL project is to identify challenges and opportunities for improvement to subsequently develop socio-culturally appropriate, tailored, innovative interventions for the successful implementation of appropriate IYCF (Infant and Young Child Feeding) practices locally.

Design: Qualitative research method, involving 5 phases, (1) identification of local feeding practices; (2) identification of the local needs and opportunities for children aged 6-24 months; (3)-(5) analysis of the gathered qualitative data, intervention design, review and distribution.

Setting: Nine villages in two community development blocks, i.e. Ghatol and Kushalgarh, located in the Banswara district in Rajasthan, India.

Participants: 68 participants completed semi-structured interviews or focus group discussions including: mothers, grandmothers, Auxiliary Nurse Midwife, Anganwadi Worker, ASHA Sahyogini, school teachers, and local elected representative.

Phenomenon of Interest: IYCF practices and the factors associated with it.

Analysis: Thematic analysis.

Results: Our results could be broadly categorized into two domains: (1) The current practices of IYCF and (2) The key drivers and challenges of IYCF. We explicate the complex phenomena and emergent model focusing upon: mother's role and autonomy, knowledge and attitude towards feeding of young children, availability of services and resources that shape these practices set against the context of agriculture and livelihood patterns and its contribution to availability of food as well as on migration cycles thereby affecting the lives of 'left behind' and access to basic health, education and infrastructure services.

Conclusions: This interdisciplinary and participatory study explored determinants impacting feeding practices across political, village and household environments. These results shaped the process for co-creation of our context-specific intervention package.

Word count: 281

For peer review only - http://bmjopen.bmj.com/site/about/guidelines.xhtml

Keywords: Feeding practices; Children; Infant; Early Interventions**;** Participatory; Community.

Article Summary

Strengths and Limitations of this study

Strengths

- As an interdisciplinary study, the PANChSHEEEL (Participatory Approach for Nutrition in Children: Strengthening Health Education Engineering and Environment Linkages - HEEE) project explored a plethora of themes across the four domains of health, education, engineering and environment (HEEE) to evaluate contributing factors, current challenges and potential opportunities in Infant and Young Child Feeding (IYCF) practices in India. Using the qualitative data presented in this paper, the ultimate objective was to co-develop a context-specific multisectoral HEEE Nutrition intervention package that is socio-culturally appropriate, targeted and innovative to enhance IYCF practices 6-24 months aged children locally.
- This study employed a bottom-up approach, involving a close collaboration with the local affected population, interdisciplinary teams of researchers, non-government organizations (NGOs), as well as pre-existing and emergent research evidence in identifying current feeding practices, as well as perceived barriers and future opportunities for intervention.

Limitations

- The study was conducted on a relatively limited geographic scale, with data gathered merely across two community blocks in Rajasthan. Further research be conducted in neighboring rural areas in India will be needed to ensure the generalizability of the findings.

For peer review only - http://bmjopen.bmj.com/site/about/guidelines.xhtml

Introduction

The Global Nutrition Report (2018) reported that globally 150.8 million and 50.5 million children are stunted (inadequate height for age) and wasted (inadequate weight for height) respectively [1]. With 38.4% stunted children, India contributed a third of the world's burden for stunting. With respect to wasting, India accounts for an even larger proportion, with 25.5 million children affected nationally – a number equivalent to half of the global burden of wasting [2]. Conscious of the fact that adequate nutrition enhances cumulative lifelong learning capacity and adult productivity, the Government of India (GOI) recognizes that improving nutrition will be of vital importance in poverty alleviation and the country's economic development in the long-run [3].

A variety of conceptual models have been used to explain the myriad and complex set of undernutrition determinants. One such model, proposed by the United Nations Children's Fund [4], illustrates the socio-ecological determinants influencing undernutrition. According to this model, inadequate dietary intake and infectious diseases are two key proximate factors for undernutrition alongside household food insecurity, inadequate care and feeding practices and unhealthy household environments.

Infant Young Child Feeding (IYCF) practices entail initiation of breastfeeding in the first hour of birth, exclusive breastfeeding till 6 months followed by complementary feeding after sixth months as well as optimal feeding practices essential for children until the age of two. Complementary feeding includes the introduction of age-appropriate semi-solid food alongside breast milk after six months of exclusive breast feeding [5]. Universal coverage of optimal breastfeeding can prevent 13% of global deaths in children less than five years of age, while appropriate complementary feeding practices could result in an additional 6% reduction in under-five mortality [6]. With 80-85% of brain growth taking place during the first two years of life, optimal IYCF plays an essential role also with respect to cognitive development, as reiterated by The Global Nutrition Report [7].

When comparing India's indicators with the global and regional indicators, two important phenomena become apparent: (i) India's breastfeeding indicators, while nearly at par with the global and South Asian averages, are lower than neighboring Sri Lanka and Bangladesh; and, (ii) India's complementary feeding indicators are lagging not only the global and South Asian averages, but also neighbouring countries (Table 1).

For peer review only - http://bmjopen.bmj.com/site/about/guidelines.xhtml

Table 1: IYCF Indicators Comparison: Global and selected South Asian countries (in %)

Indicators	Global	South Asia	India	Sri Lanka	Bangladesh
Early initiation of breastfeeding	44	40	41.5	90	50.8
Exclusive breastfeeding under 6 months	41	54	54.9	82	55.3
Introduction of solid, semi-solid or soft foods (6-8) months	70	51	42.7	N.A	64.7
Complementary feeding – Minimum Dietary Diversity	29	20	19.9	N.A	26.6
Complementary feeding – Minimum Meal Frequency	52	43	35.9	N.A	63.6
Complementary feeding – Minimum Acceptable Diet	18	12	9.6	N.A	22.8
Source: UNICEF (2019) [8]					

Complementary feeding rates declined in India between 2005-06 and 2015-16 from 52.6% to 42.7%. The recent National Family and Health Survey (NFHS) data reported that a mere 9.6% of children aged 6-23 months were receiving an adequate diet. There is marked intra-country variation with some of the highest declines in states with better/stronger health systems. (Table 2).

Table 2: Trends in IYCF indicators in India (in %)

	States	Children receiving solid or semi-solid food and breast milk (%)		Trends in IYCF (%)
		NFHS 3 (2005 – 2006)	NFHS 4 (2015 – 2016)	
**India**		52.6	42.7	-9.9
**North**	Rajasthan	38.7	30.1	-8.6

For peer review only - http://bmjopen.bmj.com/site/about/guidelines.xhtml

	States	Children receiving solid or semi-solid food and breast milk (%)		Trends in IYCF (%)
		NFHS 3 (2005 – 2006)	NFHS 4 (2015 – 2016)	
	Punjab	50.9	41.1	-9.8
	Uttar Pradesh	41.2	32.6	-8.6
**North East**	Sikkim	85.4	61.8	-23.6
	Manipur	77.4	78.38	1.4
	Arunachal Pradesh	80.2	53.6	-26.6
**West**	Gujarat	54.1	49.4	-4.7
	Maharashtra	45.5	43.3	-2.2
**Central**	Madhya Pradesh	46	38.1	-7.9
	Chhattisgarh	49	53.8	4.8
**East**	Bihar	54.5	30.7	-23.8
	Jharkhand	60.2	47.2	-13
	West Bengal	47.1	52	4.9
	Odisha	65.4	54.9	-10.5
**South**	Karnataka	69.7	46	-23.7
	Tamil Nadu	81.2	67.5	-13.7
	Kerala	93.9	63.1	-30.8

Source: Dasgupta, Chaand and Rakshit (2018) [9]

For peer review only

Disaggregated data from the NFHS 4 [10] point to Rajasthan recording the lowest IYCF indicators (Table 3). Within the state Banswara District, a predominantly tribal area, only; 39.4% children under age 3 years were breastfed within one hour of birth; and 56% children under age 6 months were exclusively breastfed. Only 0.9% and 0.8% breastfeeding and all children age 6-23 months respectively received an adequate diet.

For peer review only - http://bmjopen.bmj.com/site/about/guidelines.xhtml

Table 3: Child Feeding Practices across states, NFHS 4

States	Children age 6-23 months receiving an adequate diet (India: 9.6%)	Children age 6-8 months receiving solid or semi-solid food and breast milk (India: 42.7%)	Breastfeeding children age 6-23 months receiving an adequate diet (India: 8.7%)	Non-breastfeeding children age 6-23 months receiving an adequate diet (India: 14.3%)
Bihar	7.5	30.7	7.3	9.2
Jharkhand	7.2	47.2	7.2	7.1
Madhya Pradesh	6.6	38.1	6.9	4.8
Chhattisgarh	10.9	53.8	11.1	8.4
Odisha	8.5	54.9	8.9	5.0
Rajasthan	3.4	30.1	3.4	3.7
Assam	8.9	49.9	8.7	10.8
Uttar Pradesh	5.3	32.6	5.3	5.3
Karnataka	8.2	46.0	5.8	14.4
Kerala	21.4	63.1	21.3	22.3
Tamil Nadu	30.7	67.5	21.4	47.1
Maharashtra	6.5	43.3	5.3	12.2

For peer review only

Several Indian studies have sought to unravel some key aspects of India's malnutrition scenario, addressing important determinants at three levels: (i) household (maternal time constraint, dwindling family size, mother's age and education; lack of adequate knowledge; poor uptake of existing nutritional services; child targeted market with wide availability and consumption of ready-to-eat marketed food items); (ii) community (social and economic context; feminization of agriculture; fragile food security/seasonal food paucity due to less focus on food crops and vegetables; dwindling livestock – especially milk producing animals; low connectivity to remote locations; migration; exposure to media); and, (iii) government (inadequate and unresponsive Integrated Child Development Scheme [ICDS] and health care system; paucity of technical knowledge among service providers regarding IYCF). Several studies over the last

For peer review only - http://bmjopen.bmj.com/site/about/guidelines.xhtml

decade have examined varying associations between household environmental characteristics and stunting in under-five children, highlighting the need for interdisciplinary research [9] [11-16].

Our study, the Participatory Approach for Nutrition in Children: Strengthening Health, Education, Environment and Engineering Linkage (PANChSHEEEL) funded by the Medical Research Council (UK) was designed to: (i) explore health, education, engineering and environment (HEEE) factors that influenced Infant and Young Child Feeding (IYCF) practices and (ii) develop a socio-culturally appropriate, tailored, innovative and integrated cross sector HEEE package to support optimal IYCF practices. This is central to the convergent action planning process of the POSHAN Abhiyan (Mission) that has IYCF as the first target to be monitored.

Conceptual framework

Drawing upon the review of the above studies, the principal investigator and the co-investigators synthesized the determinants from the literature review to frame the HEEE conceptual framework as a socio-ecological model (Figure 1). This framework sought to synthesize the complex interplay of factors across three environments: political, village and household; and the inter-linkages between institutions, initiatives (schools, health services, ICDS and public distribution system) and communities. The qualitative studies that were reviewed focused on undernutrition or stunting and not specifically on IYCF and child feeding practices that have assumed crisis proportions but have received scanty attention among public health nutrition researchers in India.

Figure 1: HEEE Conceptual Framework

The formative phase of the PANChSHEEEL study was thus framed to develop an understanding of a deep-dive interplay of the determinants of IYCF to inform the co-designing of an intervention model that can address this crisis in such contexts.

This paper presents the qualitative findings of the PANChSHEEEL study; that aims to identify and document local community IYCF practices including water, sanitation & hygiene (WASH) and energy practices, to identify local challenges, drivers, resources, opportunities and needs for IYCF in 6-24 month

For peer review only - http://bmjopen.bmj.com/site/about/guidelines.xhtml

old children at individual, household, community and environmental level; and, to map the linkages between identified opportunities and challenges in order to determine how the needs identified can be addressed.

Methodology

The larger PANCHShEEEL study used both qualitative and quantitative research methods, triangulating the two to synthesize evidence. This paper presents the qualitative component of the formative study, in line with COREQ guidelines.

Study setting

The study was conducted in nine villages in two community development blocks (herein after, blocks) of Banswara district in Rajasthan, India. These two blocks, Ghatol and Kushalgarh, were purposively chosen to represent district diversity. The Ghatol Block is located in the 'command area' of the Mahi River Dam; all villages are irrigated by canals and have multiple crops yearly. In contrast, Kushalgarh is dry and semi-arid with poor irrigation; consequently, most villages are mono-crop areas. The village selection process is represented in the following consort diagram (Figure 2).

Figure 2: Consort Diagram for selection of Study Villages

Patients and Public involvement and Engagement

In line with PANChSHEEL participatory approach, considerable efforts were placed on Patient and Public Involvement and Engagement (PPIE). Suitable individuals and representatives from both the Ghatol and Kushalgarh Block were identified and subsequently engaged in all steps of the study, i.e. protocol development, study design, results dissemination.

The Core Research Team was further supported by Community Researches from the two study blocks who aided in steering the project phases locally. With the input from 'Save the Children' Rajasthan, two well-suited individuals were identified who had been proactive collaborators in previous projects by 'Save the Children' India. They were mainly involved in selecting suitable candidates who could engage actively in project activities, as well as coordinating with the Community Champions (CCs), who were crucial in establishing networks with potential study participants among Community Members (CMs).

For peer review only - http://bmjopen.bmj.com/site/about/guidelines.xhtml

Relevant stakeholders were identified and selected in a stakeholder mapping workshop as part of the formative study phase. Through snowballing, i.e. a recruitment method where existing participants are involved in the identification and recruitment of future participants using their social networks, the research team remained open to potential new stakeholder(s) identified during the data collection process. The recruitment of study participants was based on willingness to participate and availability.

Respondent selection

The respondents included stakeholders who play a critical role in feeding and caring for young children. There are three cadres of frontline workers at the village level in Rajasthan: (i) the Auxiliary Nurse Midwife (ANM), the multi-purpose female health worker; (ii) the Anganwadi Worker (AWW), the cadre of the Integrated Child Health Services (ICDS) who runs the Anganwadi Centre (AWC) at each village; and (iii) the ASHA Sahyogini (AS), the community health worker (ASHA in other states). One ANM was selected from each of the sub-centers in the study villages; in case of sub-centers with 2 ANMs, the senior ANM was selected. At the village level, all ASs, AWWs, school teachers and local elected representatives were selected. At household level, mothers and grandmothers (with at least one 6-24 month old child) were identified and selected either during the household survey or by frontline health workers.

Data collection

Key Informant Interviews (KIIs) and Focus Group Discussions (FGDs) were used for collecting primary data. Semi-structured open-ended interview guides were used to understand respondent knowledge and feeding practices perceptions. FGDs were conducted with mothers and grandmothers. The date, time and meeting place were decided in consultation with AWW and AS. FGD guides included topics on child feeding, maternal time use and WASH practices. Participants were encouraged to share their frank opinions, irrespective of the views of others in the group, in order to elicit insights about IYCF issues (Table 4).

FGDs were conducted by community researchers from the same blocks or adjoining block who were able to communicate fluently in the local dialect, *Wagdi.* They were trained on FGD techniques by the principal investigator and co-investigators which included principles and methods of qualitative research with a special focus on interview and group discussions, and an outline of data analysis. Hands-on support was provided by other team members who were well versed with the research methods and local contexts. The duration of the interviews ranged between 25-40 minutes and the FGDs ranged between 45-75 minutes.

For peer review only - http://bmjopen.bmj.com/site/about/guidelines.xhtml

We relied on a hybrid data saturation approach: achieving both: (i) a priori thematic saturation (the degree to which identified codes or themes are exemplified in the data) that informed sampling strategy and (i) inductive thematic saturation (the emergence of new codes or themes) that informed data analysis [17]. Field notes based on observations played a crucial role in data collection and analysis. Digital recorders were used to record all KIIs and FGDs. Written consent was taken from key informants whilst for FGDs verbal consent was sought and formally recorded. Audio data was then transcribed (from local language to Hindi) and translated (from Hindi to English) with back translations done for quality assurance. Both audio data and text were anonymised to maintain confidentiality and securely stored. A pilot round was conducted in January 2018 following which formal data collection continued for four months. Caution was maintained regarding selection bias of FGD participants due to the active role of frontline health workers in service delivery. FGDs were organized at Integrated Child Development Services (ICDS) or Anganwadi Centers (AWC) centers at each village with care taken to ensure that residents residing far from AWCs were not excluded. This study was conducted according to the guidelines laid down in the Declaration of Helsinki and all procedures involving research study participants were approved by the UCL ethics [Ethics ID 4032/002] and Sigma IRB [10025/IRB/D/17-18].

Topic Guide

Through the means of a literature review, expert advice and pilot testing with different population subsets, a thematic guide was designed prior to the qualitative data collection process. This guide facilitated the conductance of the KIIs and FDGs, allowing the topic of IYCF to be explored across the four major dimensions (1) Health and Nutrition, (2) Education, (3) Water, Sanitation and Hygiene and (4) Energy to capture current practices, barriers and possible opportunities to foster positive change across all four areas.

Data processing and analysis

The research team undertook daily meetings to reflect upon their key observations and data collected from field notes. Data was transcribed and translated to bring out common major themes based on commonality and differences. Data generated from all sources were triangulated and linked thematically for key findings. The data was coded and analyzed jointly by the manuscript authors (co-investigators) to ensure consistency and reliability in interpretation. Codes were analyzed across axial and selective codes across respondent categories and sites (villages and blocks). Triangulation was performed across methods and respondents. Results from KIIs and FGDs were compared and assessed for similarities and differences across respondent

For peer review only - http://bmjopen.bmj.com/site/about/guidelines.xhtml

For peer review only

categories. Findings from villages within a block conveyed similar interpretations and hence reinforced data validity. The investigator group reached a consensus to merge village and block level findings, except in those themes or sub-themes where they presented novel findings. Data analysis was first completed independently by interviewers followed by a series of joint sessions between interviewers and co-investigators.

Respondent validation

A series of meetings were conducted with representatives from all respondent categories from the same villages to validate the data. Within the qualitative data sets, all steps of a thematic analysis, i.e. (1) identifying, (2) analyzing and (3) interpreting patterns of meaning, i.e. themes, were employed. Respondent validation was facilitated by Community Researchers in September 2018 and closely overseen by local Save the Children staff. This enabled us to refine the emergent themes and model to its final shape.

Quality assurance

The inter-disciplinary team comprised of experts in public health nutrition, epidemiology, social sciences, paediatrics, education and civil and environmental engineering. A dedicated team of co-investigators supervised training, data collection, transcription and translation, data management and analysis. Field teams were oriented with the study protocol, tools and research techniques, including hands-on exercises, before actual data collection began.

Results

A total of 68 participants were recruited (table 4)

Table 4: Respondents Categories and Methods

Category of Respondent	Method Used	Numbers
Mothers and Grandmothers	FGD	17
ANM	KII	7
AWW	KII	13
ASHA Sahyogini	KII	13
School Teacher	KII	9
Local elected representative	KII	9

For peer review only - http://bmjopen.bmj.com/site/about/guidelines.xhtml

Deriving from the study objectives, this section is divided into two parts. The first section provides an in-depth description of IYCF practices. The second section will explain the factors (drivers and challenges) associated with it. Online supplementary table illustrates the themes identified and example quotes from focus groups and interviews with respondents.

IYCF practices

Figure 3. Infant and Young Child Feeding (IYCF) – Current Practices

✶ *Core theme 1: Partial breastfeeding is a common practice in first six months.*

Most mothers and grandmothers reported that breastfeeding was initiated within the first two to three hours of childbirth in hospital. Delay in breastfeeding was reported mainly due to "*secretion of milk not starting*." [Grandmothers FGD, Kushalgarh].

"*If the baby is delivered at home breastfeeding is starts immediately after birth.*"
[AS, Kushalgarh]

Giving colostrum was considered mandatory among a majority of respondents, with only a minority of grandmothers in Kushalgarh expressing negative views about colostrum as "dirty milk". All respondents denied giving any pre-lacteals like honey to the children though these practices were reportedly followed in cases of home births, albeit very few.

Most mothers in Ghatol reported that they exclusively breastfed for four to six months. In Kushalgarh, a majority of respondents breastfed till the third month. Breastfeeding frequency in the first six months was reported to be 'on cue', as and when the child cried.

"*Mothers of children who are on breast milk work inside the village, so they can be summoned home whenever the child cries*"
[Mothers, Ghatol]

Mothers who resumed their agricultural work after three months reported coming home from work every two hours for breastfeeding. On further prompting it was revealed that, at times, water was given to children below six months, especially during summer months due to the belief that the child gets thirsty. Dissolving biscuits in milk or water and feeding it to children in case they cried or the mother was not available

For peer review only - http://bmjopen.bmj.com/site/about/guidelines.xhtml

appeared to occur on occasion, thus compromising exclusive breastfeeding. This practice was also promoted by FHWs. Animal milk, especially goat milk, was given when the mother was unavailable or when the mother's milk amount was perceived to be inadequate.

"*In the summer season the child becomes thirsty, so water is given to the child. We also recommend giving water sometimes*."
[AWW, Ghatol]

For peer review only

✶ *Core theme 2: Biscuits given as complementary food after six months*

Consumption of biscuits as convenience foods increased after six months in both blocks. Soaked in water or tea, they were used as a semi-solid food for local children. Two reasons were cited for this practice: (i) easy availability and low costs (5 INR or 0.7 USD per packet) and, (ii) children liked the sweet taste; hence making it easy to feed.

"*Children like biscuits and it is easy to feed them.*"
[Grandmothers FGD, Kushalgarh]

"*This is the cheapest option and is easily available in the village.*"
[Mothers FGD, Ghatol]

These were given in the morning as the first meal of the day, and/or between meals when children cried. FHWs commonly approved of this practice.

"*We tell the mothers that they can give biscuits to the child, at least it will keep them full*."
[ANM, Kushalgarh]

Biscuits were often employed as a method to *pacify* the child when the mother or other care givers were busy in their daily chores. The number of biscuits given per day varied from five to ten per day.

For peer review only - http://bmjopen.bmj.com/site/about/guidelines.xhtml

✶ *Core theme 3: Dietary preferences and recent shifts*

On average, complementary feeding was commenced around 7-8months. The main reason of delayed complementary feeding was the child's "lack of interest" in eating semi-solid foods, marked by prolonged feeding time, crying and also fidgeting while eating. A majority of participants reported to be confident regarding appropriate complementary feeding.

"*The baby starts drinking milk (dairy) after 6 months. We know what to give and what not to.*"

[Mothers FGD, Ghatol]

The foods included pulses (in watery consistency), *khichdi* (savoury rice and pulse gruel), *dalia* (porridge with ground wheat with milk or water, mostly sweet) or small pieces of roti dipped in milk. The use of commercial baby food was nominal. FHWs reported that children were fed four to five times a day on average; mothers and grandmothers, however, reported demand feeding to be common practice, i.e. feeding when the children cried.

"Whenever the children get hungry we feed them."

[Mothers FGD, Kushalgarh]

Most mothers from both blocks reported that, because children ate in small quantities, it was difficult to cook separately for them every day. Neither FHWs nor the mothers had clarity about the frequency and quantity to be fed to children. Portion size varied depending on the child's "interest". All mothers confirmed that they received packets of Take-Home Ration (THR) regularly, but had little knowledge of the proper recipe.

Consumption of non-vegetarian food was dwindling while those who consumed animal products, e.g. meat and eggs, did so discretely. This was attributed to religious reasons, locally termed the '*bhagat'* (disciple) culture.

"This is a recent trend. Hawan (worshipping with holy fire) was conducted in the village after which every family has stopped eating. No question of giving it to children."

[Ward Panch, Ghatol]

This practice was more prevalent in Ghatol, where more respondents ascribed to these beliefs.

For peer review only - http://bmjopen.bmj.com/site/about/guidelines.xhtml

In Kushalgarh, hens were reared in most households, consequently resulting in a higher availability and consumption of non-vegetarian foods. However, due to religious values, respondents often denied its consumption. .

"This is made only if there is a guest at home or there is any occasion.
Chicken is mostly eaten (on such occasions)."
[Teacher, Kushalgarh]

Responses from both blocks confirmed that children under five years were rarely given non-vegetarian food, and if so, only in small quantities.

Consumption of fruits, though unanimously considered to be beneficial, was rare on account of availability and cost. Fruits are generally available in the markets located far away from the villages; more so in Kushalgarh. Purchase was possible only when a family member visited these markets.

"*Fruits are not very common in daily diet. Only when the parents go to the market they get fruits."*
[Teacher, Kushalgarh]

Vegetables consumption also comprised "*potatoes, tomatoes and onions almost daily*." [ANM, Ghatol] There was less consumption of green leafy vegetables, for children, even though it was readily available.

Key Drivers and Challenges

IYCF practices described in the previous sections are attributable to contextual issues emerging at the household, community and government level. Differences evident between Ghatol and Kushalgarh include cropping patterns, market access and fruit and vegetable availability. These differences aside, there were similarities in the drivers of IYCF practices between these two blocks.

Figure 4. Infant and Young Child Feeding (IYCF) – Key Drivers and Challenges: Connecting subthemes.

✶ *Core theme 4: Time-constrained mother and role of other family members*

For peer review only - http://bmjopen.bmj.com/site/about/guidelines.xhtml

Time constraints amongst mothers emerged as a critical determinant accounting for lack of exclusive breastfeeding (infants below six months) and feeding (home cooked food) at regular intervals (to older children).

"*The mother cannot sit at home taking care of the child. Who will look after the animals, get water or cook? They also have to do agricultural work*."
[Grandmothers FGD, Kushalgarh]

All mothers, while being primary caregivers, reported that they were guided and influenced by grandmothers, other family members and FHWs. Mothers stayed at or worked in close proximity to their homes until the child was about two months old. During this time, they continued with cooking and other household chores, as well as outdoor work, i.e. firewood and water collection. Mothers from Ghatol reported that they did not go out for work outside the villages. Occasionally, mothers took up daily work in nearby factories, but only once their child was at least one year of age. Women in Kushalgarh migrated to nearby towns and districts as a family, once children were above six months.

"*When my daughter-in-law works outside, then we grandmothers take care of the children*."
[Grandmothers FGD, Ghatol]

Teachers reported that also elder siblings, especially girls, were often absent from school to take care of younger siblings, especially in families where both parents had migrated (seasonally) or if the primary care giver was unavailable.

✶ *Core theme 5: Livelihood challenges in a chronic poverty setting*

Agriculture is the main source of livelihood and revenue for families in both blocks, despite clear differences in irrigation facilities and cropping pattern in Ghatol and Kushalgarh.

"*Most people here are in agriculture. Both males and females of all families take part in agriculture.*
[School Teacher, Kushalgarh]

Women in all study villages were engaged in agriculture and livestock farming throughout the year; men took part in agriculture only during sowing and harvesting seasons. Some women were engaged under the National Rural Employment Guarantee Scheme though employing mothers with children aged less than

For peer review only - http://bmjopen.bmj.com/site/about/guidelines.xhtml

two years was not common practice. Local wage labor was reported to be a common source of income for men residing in Ghatol villages due to the proximity to the district town and a cloth mill. Circular migration to urban areas of the adjacent states of Madhya Pradesh and Gujarat was common among men in Kushalgarh.

"*Some men from the village work in Mayur mills (textile mill near Banswara). Some also work as wage labour, but only during lean seasons.*" [School Teacher, Ghatol]

For peer review only

✶ *Core theme 6: Childcare services fails to deliver on most counts*

Proactive steps are being taken by government programs to address IYCF related challenges. Breastfeeding information was mostly provided at childbirth facilities and, these practices were much better compared to complementary feeding. The overwhelming evidence from all the villages was that level of knowledge and counseling skills were poor among AWW and AS. They were not aware of malnutrition indicators and appropriate complementary foods, often promoting the introduction of animal milk, semi-solid or solid food into a baby's diet before the age of six months. Infant weight was recorded in some cases.

"We take weight, but currently the weighing machine is not working."
[AWW, Kushalgarh]

In many villages, several (non-health) key informants reported that AWWs mostly catered to community needs staying close to the AWC. The supervisory cadres rarely visited the villages.

"*Neither LS (Lady Supervisor) nor the CDPO (Child Development Project Officer) visits our centre. We only go to the sector meetings where they ask us about the number of pregnant women registered, children born and packets of THR distributed. "*
[AWW, Kushalgarh]

Ghatol received more support from the district level than Kushalgarh owing to its proximity to the headquarters; this was further complemented by the presence of NGOs and other civil society actors in Ghatol.

✶ *Core theme 7: Emerging role of schools and panchayats in health and nutrition programs*

For peer review only - http://bmjopen.bmj.com/site/about/guidelines.xhtml

School teachers were an important resource for local communities; the schools functioned regularly and efficiently with teachers attending school daily. Mid-day meals were provided. With male members mostly working outside the village (either daily wage or migration), parents' participation in these meetings were occasional. According to school teachers (mostly male), fathers interacted with them with few mothers participating. The state has recently formulated at policy for co-location of AWC at school premises.

"*The AWC is in the school premises; we can always keep a check whether children are getting their meals. The AWW and the other staff also come in time and regularly open the centre*."
[School Teacher, Kushalgarh]

Many teachers reported taking a personal interest in visiting and monitoring the functioning of AWCs; however due to their own busy schedule and excessive workload (in part due to staff shortage), they were not always proactive.

"*We are already overburdened with the work of the school. It is not possible to check the functioning of the AWC.*"
[School Teacher, Ghatol]

All the ward panches (elected Panchayat [local self-government] member) were unaware of the Village Health Sanitation and Nutrition Committees (VHSNC) and played no role in this critical village level platform for promoting health and nutrition of mothers and children.

✶ *Core theme 8: WASH practices can play a vital role*

Caregivers were unanimous in their perception that illnesses such as diarrhea, fever or cough represented one contributing factor of inappropriate nutrition among children. There was little awareness that these diseases may be partially linked to WASH deficiencies. Hand pumps were the only source of drinking water in both blocks. Unlike in Ghatol where a majority of hand pumps have water throughout the year, some sources in Kushalgarh dry up "*due to scarcity of water*" [ANM, Kushalgarh] during the summer months. The only measure for drinking water treatment was using a cloth for filtering the water while filling; this practice (as reported by the FHWs) was more common in Kushalgarh than in Ghatol. In several villages of Kushalgarh, a local reverse osmosis plant using solar panels was set up under a new scheme by the Public Health and Engineering department.

Mothers, grandmothers and other key informants reported that communities in Ghatol were more willing to construct sanitary facilities than those in Kushalgarh, even though there is a national sanitation program which incentivizes toilet construction at household level.

For peer review only - http://bmjopen.bmj.com/site/about/guidelines.xhtml

"Those who constructed the toilets have not got their due payments. I have been following up with the Sarpanch's office regularly, but there is no response. This discourages those who have not yet constructed."

[Ward Panch, Kushalgarh]

In both blocks, however, a majority of households had access to functional toilets.

"The government has constructed toilets in every household. Whatever facilities the government has provided us, I have provided in this village."

[Ward Panch, Ghatol]

Most members reported using toilets, with the exception of a few elders who prefer open defecation. None of the under-two children defecated in toilets. Small children defecated on pieces of cloth which were subsequently disposed of in open fields near the house. The older children defecated in the open, near their homes, except some in Ghatol who were reported to use toilets.

Awareness on the importance of hand washing was high in all study villages. Hand washing with water and soap before cooking and eating was unanimously reported in Ghatol, but less so in Kushalgarh due to water scarcity.

Discussion

IYCF practices can be categorized as optimal and suboptimal. Optimal practices such as early breastfeeding initiation, reduction in pre-lacteal use, mothers being encouraged to exclusively breastfeed the child (till six months) and continue breastfeeding till two years were reported in our study villages. Suboptimal practices were more widespread and included giving water in the first six months, feeding biscuits from the fourth month and complementary feeds lacking in quantity, frequency and diversity. The emergent model bears out the interactions of the socio-ecological elements framed in our conceptual framework (Figure 5).

Figure 5: Emergent Model

It is in this backdrop that we sought to identify factors that influence and shape these existing practices and then design interventions that are able to address barriers and promote improvement. The evidence

For peer review only - http://bmjopen.bmj.com/site/about/guidelines.xhtml

presented in this paper forms the basis of the socio-culturally appropriate, tailored, integrated and interdisciplinary interventions developed as part of the PANCHSHEEL project drawing on the sectors of: (i) health and nutrition, (ii) education and (iii) engineering and environment.

Most of the facilitators and barriers were in the realm of knowledge and skills. The two most common barriers across most household were lack of appropriate knowledge of mothers and other caregivers about: (i) complementary feeding practices (frequency, quantity and quality) and, (ii) recipe for cooking Take Home Ration (supplementary nutrition) distributed by the Anganwadi Centre. The knowledge gaps also resonated with incomplete information that was provided by the FHWs; this was also one of the barriers to promoting positive IYCF practices. Two such (highly prevalent) practices were giving water to babies less than 6 months and feeding biscuits. We also noted the divergence between perceptions of mothers and grandmothers. This was largely on account of mothers caring and feeding the children more actively in contrast to grandmothers who looked after children largely when mothers were away for work and were not often the final decision makers of care and feeding practices.

Resource constraints mothers emerged as another significant factor. Lack of mother's time to continue exclusive breastfeeding as well as dedicating time for separate cooking and feeding was one of the commonest barriers to positive IYCF practice. As male members travelled to nearby towns for daily wage labor or migrated to nearby states, (owing to lack of employment opportunities in the villages), women were compelled to dedicate more time to agricultural work. The poor environmental conditions and limited access to basic water and sanitation services also contributed to the time and resource constraints faced by women.

Little or no availability of animal milk resulted in children between 6 months to 2 years hardly receiving any milk in their diet. Limited use of vegetables and fruits was due lack of markets, inadequate resources to cultivate and high cost. As a result, children's diets were seldom rich in iron and vitamins. The absence of adequate resources was not merely a household level issue, but also rooted in the local cultural and religious contexts. The remaining two determinant categories; motivation and attitude, were mostly linked to either knowledge or resources.

Delineation of these factors helped in identifying key 'actors' who could bring about improvement in IYCF practices. To ensure continued support to mothers and other caregivers on positive IYCF practices, the knowledge and skills of FHWs, especially ASHA Sahyogini and Anganwadi Workers, need to be upgraded. Finally, there is a need for garnering community support for these children for which the role of important members like school teachers and elected representative was also identified as crucial.

For peer review only - http://bmjopen.bmj.com/site/about/guidelines.xhtml

This study, and the urgent need for IYCF interventions, assumes significance in the light of the recently released Global Hunger Index 2019 data [18]. India's Global Hunger Index (GHI) score declined from 38.8 in 2000 (rank: 83 out of 113 countries) to 30.3 in 2019 (rank: 102 out of 117 countries), placing the country in the 'serious' category. Achieving internationally agreed targets on stunting and wasting in under-five children is key to achieving Sustainable Development Goals. The complex set of factors and barriers to appropriate IYCF practices – captured in the HEEE conceptual framework and the qualitative data – pose a challenge in achieving the SDGs. While the Government of India has designed and implemented several programs to address these barriers, they reported limited success primarily because of the unifaceted nature of these interventions as opposed to much needed multifaceted approach. The task at hand is therefore a well-designed, locally feasible, multi-sectoral interventions across health and nutrition, education as well as water and sanitation. Through this study we demonstrate the need for integrated and cross-sectoral research to comprehensively shape the feeding practices of children; thereby creating a scope for coordinating the ongoing interventions in a manner to achieve the desired target (the subject of another paper).

Declaration of Conflicting Interest:

The authors declared no potential conflicts of interest with respect to the research, authorship and/or publication of this paper.

Financial Support and Funding:

This work was also supported by the Global Challenges Research Fund and funded by the MRC, AHRC, BBSRC, ESRC and NERC [grant number: MR/P024114/1].

Authorship:

ML, ML, PP, RK, and SS conceived the original concept of the study and designed the research methodology. SR, ML, PP, RK, HC, SS, SPP, TS, and PP carried out the interviews, analysed the data, and wrote the paper. ML, ML, PP, SR, HC, NS, SS, SPP, and RK validate the study and revised the manuscript critically for important intellectual content. SA contributed to the manuscript writing, edited the final manuscript and prepared for submission. ML had primary responsibility for the final content. All authors

For peer review only - http://bmjopen.bmj.com/site/about/guidelines.xhtml

read and contributed to the reviewing the analysis of the data, the designing of the manuscript, and the approval of the final manuscript.

Data Statement:

The data of this study is available from the corresponding author upon reasonable request.

For peer review only

References

1. Fanzo J, Hawkes C, Udomkesmalee E, et al. (2018) Global Nutrition Report: Shining a light to spur action on nutrition.
2. International Institute for Population Sciences. National Family Health Survey (NFHS-3), 2005-06: India. International Institute for Population Sciences; 2007. Available from: https://dhsprogram.com/pubs/pdf/FR339/FR339.pdf.
3. Niti Aayog. (2017). National Nutrition Strategy. New Delhi; India: Niti Ayog, Government of India. Available from: http://niti.gov.in/writereaddata/files/document_publication/Nutrition_Strategy_Booklet.pdf.
4. UNICEF. (2017). Forest Lanterns. New Delhi: Penguin Random House.
5. World Health Organization. Infant and young child feeding: model chapter for textbooks for medical students and allied health professionals. Available from: https://www.who.int/nutrition/publications/infantfeeding/9789241597494.pdf
6. Jones AD, Ickes SB, Smith LE, et al. (2013) World Health Organization infant and young child feeding indicators and their associations with child growth: a synthesis of recent findings.
7. Gupta A, Thakur N. Infant and young feeding practices in india: current status and progress towards SDG targets. Proceedings of the Indian National Science Academy. (2018) Nov 13;84(4):853-65.
8. UNICEF. (2019). Global UNICEF Global Databases: Infant and Young Child Feeding: Minimum acceptable diet, Minimum diet diversity, Minimum meal frequency. New York; USA: UNICEF. Available from: https://www.unicef.org/nutrition/files/IYCF_programming_guide_Apr_2013.pdf
9. Dasgupta R, Chaand I, Barla KR. The slippery slope of child feeding practices in India. Indian pediatrics. 2018 Apr 1;55(4):284-6.
10. International Institute of Population Sciences. (2017). National Family Health Survey, India: Key Findings from NFHS-4 (2015-16). Mumbai, India: International Institute of Population Sciences. Available from: https://dhsprogram.com/pubs/pdf/FR339/FR339.pdf
11. Imran M, Ilyas N, Mahmood T. Infant and young child feeding (IYCF) practices. Rawal Medical Journal. 2017 Oct 1;42(4):546-9.
12. Chaturvedi S, Ramji S, Arora NK, Rewal S, Dasgupta R, Deshmukh V. Time-constrained mother and expanding market: emerging model of under-nutrition in India. BMC public health. 2016 Dec;16(1):632.

For peer review only - http://bmjopen.bmj.com/site/about/guidelines.xhtml

13. Dasgupta R, Arora NK, Ramji S, Chaturvedi S, Rewal S, Suresh K, Deshmukh V, Thakur N. Managing Childhood Under-Nutrition: Role and Scope of Health Services. Economic and Political Weekly. 2012 Apr 21:15-9.
14. Ramji S. Impact of infant & young child feeding & caring practices on nutritional status & health. Indian J Med Res. 2009 Nov 1;130(5):624-6.
15. Desai S, Banerji M. Negotiated identities: Male migration and left-behind wives in India. Journal of Population Research. 2008 Oct 1;25(3):337-55.
16. Lee C, Lakhanpaul M, Maza Stern B, Parikh P. Associations between the household environment and stunted child growth in rural India: A cross-sectional analysis. UCL Open: Environment. 2019 Mar 6.
17. Saunders B, Sim J, Kingstone T, Baker S, Waterfield J, Bartlam B, Burroughs H, Jinks C. Saturation in qualitative research: exploring its conceptualization and operationalization. Quality & quantity. 2018 Jul 1;52(4):1893-907.
18. Welt Hunger Hilfe. (2019). Global Hunger Index: The Challenge of Hunger and Climate Change. Dublin, Ireland: Welt Hunger Hilfe. Available from: https://www.globalhungerindex.org/pdf/en/2019.pdf

For peer review only

For peer review only - http://bmjopen.bmj.com/site/about/guidelines.xhtml

For peer review only

For peer review only - http://bmjopen.bmj.com/site/about/guidelines.xhtml

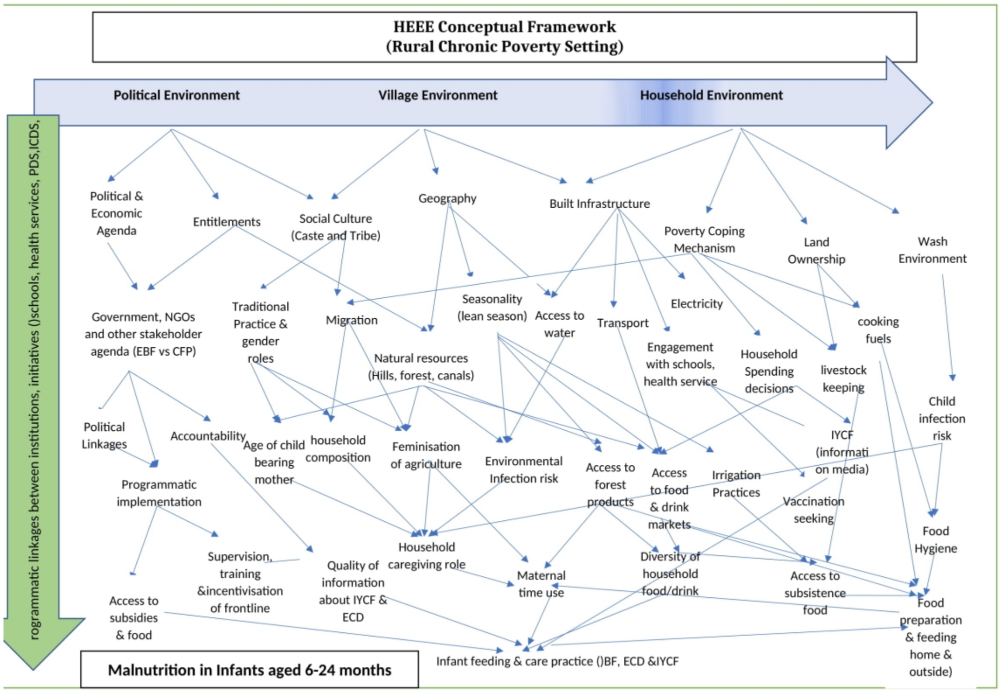

HEEE Conceptual Framework

825x583mm (72 x 72 DPI)

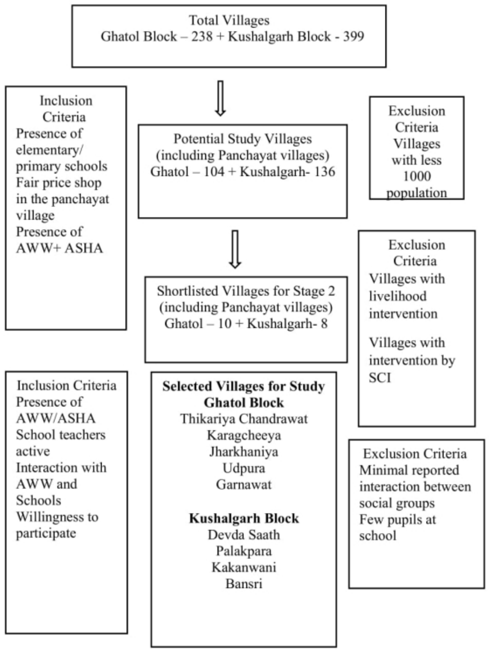

Consort Diagram for selection of Study Villages

583x825mm (72 x 72 DPI)

Figure 3. Infant and Young Child Feeding (IYCF) – Current Practices

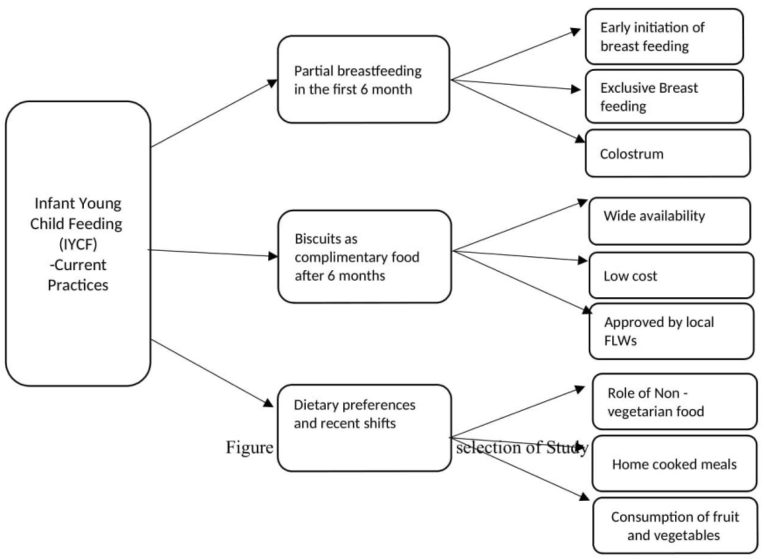

Infant and Young Child Feeding (IYCF) – Current Practices

583x386mm (72 x 72 DPI)

Figure 4. Infant and Young Child Feeding (IYCF) – Key Drivers and Challenges

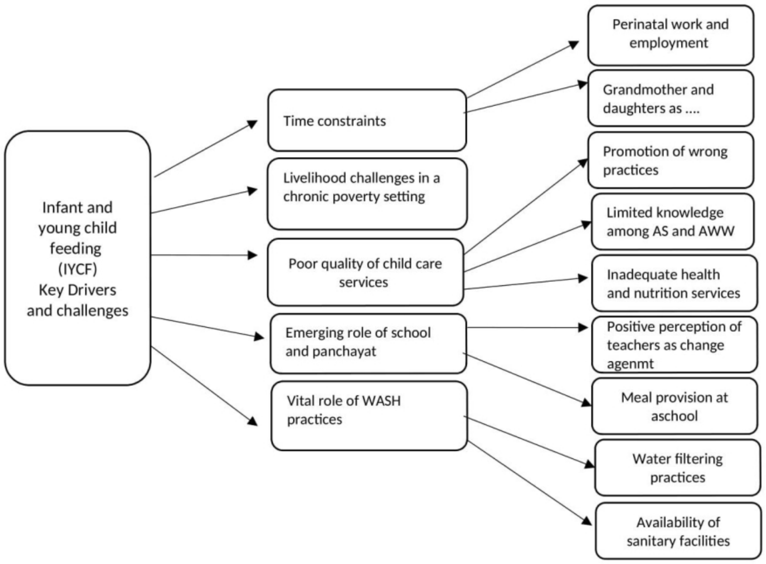

Infant and Young Child Feeding (IYCF) – Key Drivers and Challenges: Connecting subthemes.

580x443mm (72 x 72 DPI)

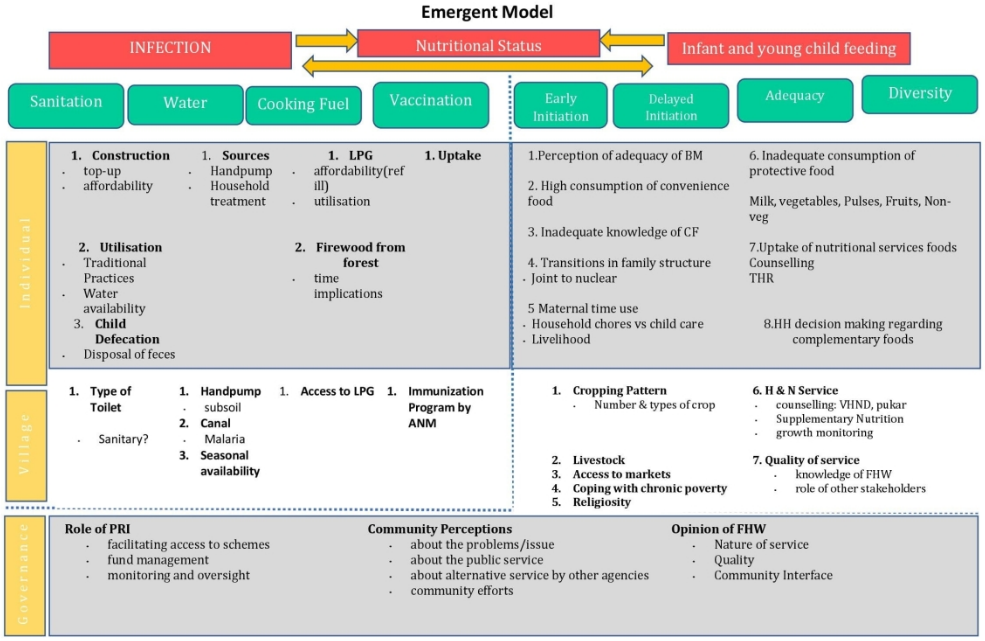

Emergent Model

297x209mm (200 x 200 DPI)

Core Theme	Sub-theme	Participant type	FDG/KII	Example Quote
**Infant and Young Child Feeding (IYCF) – Current Practices**	*Partial breastfeeding in the first 6 months*	Mothers, Kushalgarh	FDG	"*When the child starts crying after birth then breast milk is produced and the mother is ready to breastfeed the child.*"
		Mothers, Ghatol	FDG	"*No, we do not breastfeed the child soon after childbirth. Breastfeeding depends on the mother's ability to produce milk. Breastfeeding begins only after midnight if a birth occurs during the evening.*
		AS, Kushalgarh	KII	"*If the baby is delivered at home breastfeeding is starts immediately after birth. Most cases I am present during the childbirth and hence I ensure that breastfeeding starts within an hour for cases that I attend.*"
		Grandmothers, Kushalgarh	FGD	"*Only when the mother's milk secretion does not start, goat's milk is given. Honey or any water is not given.*"
		Mothers, Ghatol	FGD	"*Mothers of children who are on breast milk work inside the village, so they can be summoned home whenever the child cries"*
		AWW, Ghatol	KII	"*In the summer season the child becomes thirsty, so water is given to the child. We also recommend giving water sometimes.*"
		Grandmothers, Kushalgarh	FDG	"*When the child's mother is out of the village due to some work or is working in the field, goat milk is given when the child cries.*"
	Biscuits as complementary food after 6 months	Grandmothers, Kushalgarh	FGD	"*Children like biscuits and it is easy to feed them. We give them one biscuit and they keep eating it. We do not have to feed them.*"
		Mothers, Ghatol	FGD	"*This is the cheapest option and is easily available in the village.*"
		ANM, Kushalgarh	KII	"*We tell the mothers that they can give biscuits to the child, at least it will keep them full.*"
		Mothers, Ghatol	FDG	"*The baby starts drinking milk (dairy) after 6 months. We also give rab (grounded maize porridge), biscuits dipped in the water and sometime daal ka pani (lentil soup). We know what to give and what not to. The AWW also tells us about this. We only take care of all things related to the child like cooking and feeding.*"

For peer review only

	Dietary preferences and recent shifts	Mothers FGD, Kushalgarh	FGD	"*We feed them separately. We do not feed them with us. Whenever the children get hungry we feed them. They do not understand anything; they eat whatever we give them.*"
		Ward Panch, Ghatol	KII	"*Nobody eats non-vegetarian food as they have become 'followers'. This is a recent trend. Hawan (worshipping with holy fire) was conducted in the village after which every family has stopped eating. No question of giving it to children.*"
		Teacher, Kushalgarh	KII	"*Very few families eat non-vegetarian (foods). Those who eat also give their children. This is made only if there is a guest at home or there is any occasion. Chicken is mostly eaten (on such occasions). Eggs are not eaten as they are left to hatch and the goats are sold.*"
		Teacher, Kushalgarh	KII	"*Fruits are not very common in daily diet. Only when the parents go to the market they get fruits. There is availability of local fruits which are not too good for children, like tamarind and plum.*"
		ANM, Ghatol	KII	"*Some vegetables are grown in the backyard of every home, mostly beans and brinjal. This is used quite regularly. Potatoes, tomatoes and onion are cooked almost daily. The local vegetables like spinach are seldom grown as people do not want to eat them.*"
	Time Constraints	Grandmother, Kushalgarh	FDG	"*The mother cannot sit at home taking care of the child. Who will look after the animals, get water or cook? They also have to do agricultural work.*"
		ANM, Ghatol	KII	"*Some mothers work a lot and hence are not able to take care of the child adequately. Grandmothers help, but they are also old people and cannot do much.*"
		Grandmother, Ghatol	FDG	*When my daughter-in-law works outside, then we grandmothers take care of the children. Like when the child is crying and have to be fed; we keep them in our laps and make them comfortable. We keep an eye on the children and their activities.*"
		School Teacher, Kushalgarh	KII	"*Most people here are in agriculture. Both males and females of all families take part in agriculture. Men play an important role of sowing and harvesting while women do the rest. Women are engaged all year round.*"
		Ward Panch, Ghatol	KII	"*Most houses keep goats, some also have cows. Buffaloes are very less in the village. All of these animals do not give milk. Milk from goat is used for consumption at home. Cow*

**Infant and Young Child Feeding (IYCF) – Key Drivers and Challenges**	*Livelihood challenges in a chronic poverty setting*			*and buffalo milk is used for making ghee. Few families also sell it. They do not feed milk to their children"*
		Mothers, Ghatol	FDG	*Taking care of the animals is the work of women as they stay at home. They are the ones who clean the shed and give the animals fodder and water."*
		School Teacher, Ghatol	KII	*"Some men from the village work in Mayur mills (textile mill near Banswara). Some also work as wage labour, but only during lean seasons. Women with small children do not go out of the village to work."*
	Poor Quality of Childcare services	AWW, Kushalgarh	KII	*"Neither LS (Lady Supervisor) or the CDPO (Child Development Project Officer) visits our centre. We only go to the sector meetings where they ask us about the number of pregnant women registered, children born and packets of THR distributed. They sometime give us new information. The focus is on children coming to AWC."*
		AWW, Ghatol	KII	*"We take the weight of the child who comes for the vaccination. Sometimes the mothers do not get the card when they come for vaccination, so weight cannot be recorded in the (Mamta) card."*
		AWW, Kushalgarh	KII	*"We take weight, but currently the weighing machine is not working."*
	Emerging Role of Schools and Panchayats	School Teacher, Kushalgarh	KII	*"The AWC is in the school premises; we can always keep a check whether children are getting their meals. The AWW and the other staff also come in time and regularly open the centre."*
		School Teacher, Ghatol	KII	*"We are already overburdened with the work of the school. It is not possible to check the functioning of the AWC. However sometimes while going back from school I go to the AWC and speak to the workers. I have seen their records and gave them suggestions to maintain it better. What more can we do?"*
	Vital role of	AWW, Ghatol	KII	*"Toilets have been constructed in every household but not everyone is using it. Elder members prefer open defecation."*
		ANM, Kushalgarh	KII	*"Few families have constructed the toilets and very few members use it due to scarcity of water."*

For peer review only

	WASH practices	Ward Panch, Kushalgarh	KII	*"Those who constructed the toilets have not got their due payments. I have been following up with the Sarpanch's office regularly, but there is no response. This discourages those who have not yet constructed."*
		Ward Panch, Ghatol	KII	*"The government has constructed toilets in every household. Whatever facilities the government has provided us, I have provided in this village. Half of the villagers use toilets. There is a delay in payment, but the Sarpanch has told me that it will be soon released."*

For peer review only

COREQ (COnsolidated criteria for REporting Qualitative research) Checklist

A checklist of items that should be included in reports of qualitative research. You must report the page number in your manuscript where you consider each of the items listed in this checklist. If you have not included this information, either revise your manuscript accordingly before submitting or note N/A.

Topic	Item No.	Guide Questions/Description	Reported on Page No.
**Domain 1: Research team and reflexivity**			
Personal characteristics			
Interviewer/facilitator	1	Which author/s conducted the interview or focus group?	
Credentials	2	What were the researcher's credentials? E.g. PhD, MD	
Occupation	3	What was their occupation at the time of the study?	
Gender	4	Was the researcher male or female?	
Experience and training	5	What experience or training did the researcher have?	
Relationship with participants			
Relationship established	6	Was a relationship established prior to study commencement?	
Participant knowledge of the interviewer	7	What did the participants know about the researcher? e.g. personal goals, reasons for doing the research	
Interviewer characteristics	8	What characteristics were reported about the inter viewer/facilitator? e.g. Bias, assumptions, reasons and interests in the research topic	
**Domain 2: Study design**			
Theoretical framework			
Methodological orientation and Theory	9	What methodological orientation was stated to underpin the study? e.g. grounded theory, discourse analysis, ethnography, phenomenology, content analysis	
Participant selection			
Sampling	10	How were participants selected? e.g. purposive, convenience, consecutive, snowball	
Method of approach	11	How were participants approached? e.g. face-to-face, telephone, mail, email	
Sample size	12	How many participants were in the study?	
Non-participation	13	How many people refused to participate or dropped out? Reasons?	
Setting			
Setting of data collection	14	Where was the data collected? e.g. home, clinic, workplace	
Presence of non-participants	15	Was anyone else present besides the participants and researchers?	
Description of sample	16	What are the important characteristics of the sample? e.g. demographic data, date	
Data collection			
Interview guide	17	Were questions, prompts, guides provided by the authors? Was it pilot tested?	
Repeat interviews	18	Were repeat inter views carried out? If yes, how many?	
Audio/visual recording	19	Did the research use audio or visual recording to collect the data?	
Field notes	20	Were field notes made during and/or after the inter view or focus group?	
Duration	21	What was the duration of the inter views or focus group?	
Data saturation	22	Was data saturation discussed?	
Transcripts returned	23	Were transcripts returned to participants for comment and/or	

For peer review only

Topic	Item No.	Guide Questions/Description	Reported on Page No.
		correction?	
**Domain 3: analysis and findings**			
Data analysis			
Number of data coders	24	How many data coders coded the data?	
Description of the coding tree	25	Did authors provide a description of the coding tree?	
Derivation of themes	26	Were themes identified in advance or derived from the data?	
Software	27	What software, if applicable, was used to manage the data?	
Participant checking	28	Did participants provide feedback on the findings?	
Reporting			
Quotations presented	29	Were participant quotations presented to illustrate the themes/findings? Was each quotation identified? e.g. participant number	
Data and findings consistent	30	Was there consistency between the data presented and the findings?	
Clarity of major themes	31	Were major themes clearly presented in the findings?	
Clarity of minor themes	32	Is there a description of diverse cases or discussion of minor themes?	

Developed from: Tong A, Sainsbury P, Craig J. Consolidated criteria for reporting qualitative research (COREQ): a 32-item checklist for interviews and focus groups. *International Journal for Quality in Health Care*. 2007. Volume 19, Number 6: pp. 349 – 357

Once you have completed this checklist, please save a copy and upload it as part of your submission. DO NOT include this checklist as part of the main manuscript document. It must be uploaded as a separate file.

For peer review only

BMJ Open

Why India is struggling to feed their young children? A Qualitative analysis for tribal communities

Journal:	*BMJ Open*
Manuscript ID	bmjopen-2021-051558.R1
Article Type:	Original research
Date Submitted by the Author:	28-Dec-2021
Complete List of Authors:	Lakhanpaul, Monica; University College London, Population, Policy and Practice, UCL Great Ormond Street Institute of Child Health; Whittington Health NHS Trust<br>Roy, Susrita; Save The Children<br>Benton, Lorna; University College London, 1. Population, Policy and Practice, UCL Great Ormond Street Institute of Child Health<br>Lall, Marie; University College London, Institute of Education<br>Khanna, Rajesh; Save The Children<br>Vijay, Virendra ; Indian Institute of Technology Delhi<br>Sharma, Sanjay ; University College London, Engineering for International Development Centre<br>Manikam, Logan; University College London Institute of Epidemiology and Health Care, Department of Epidemiology and Public Health; Aceso Global Health Consultants<br>Santwani, Neha; Save the Children Fund, Health and Nutrition<br>Reddy, Hanimi; Save the Children, National Support Office<br>Chaturvedi, Hemant; Save the Children<br>Allaham, Shereen ; UCL, Department of Epidemiology and Public Health; Aceso Global Health Consultants Ltd,<br>Pattanaik, Satya Prakash ; Save the Children<br>Singh, Tol; Save the Children<br>Pandya, Pramod; Save the Children<br>Dang, Priyanka; Save the Children<br>Parikh, Priti; University Colege London, Engineering for International Development Centre, Faculty of the Built Environment, London, UK
**Primary Subject Heading**:	Global health
Secondary Subject Heading:	Evidence based practice, General practice / Family practice, Nutrition and metabolism, Paediatrics, Qualitative research
Keywords:	NUTRITION & DIETETICS, Community child health < PAEDIATRICS, PUBLIC HEALTH

SCHOLARONE™
Manuscripts

I, the Submitting Author has the right to grant and does grant on behalf of all authors of the Work (as defined in the below author licence), an exclusive licence and/or a non-exclusive licence for contributions from authors who are: i) UK Crown employees; ii) where BMJ has agreed a CC-BY licence shall apply, and/or iii) in accordance with the terms applicable for US Federal Government officers or employees acting as part of their official duties; on a worldwide, perpetual, irrevocable, royalty-free basis to BMJ Publishing Group Ltd ("BMJ") its licensees and where the relevant Journal is co-owned by BMJ to the co-owners of the Journal, to publish the Work in this journal and any other BMJ products and to exploit all rights, as set out in our licence.

The Submitting Author accepts and understands that any supply made under these terms is made by BMJ to the Submitting Author unless you are acting as an employee on behalf of your employer or a postgraduate student of an affiliated institution which is paying any applicable article publishing charge ("APC") for Open Access articles. Where the Submitting Author wishes to make the Work available on an Open Access basis (and intends to pay the relevant APC), the terms of reuse of such Open Access shall be governed by a Creative Commons licence – details of these licences and which Creative Commons licence will apply to this Work are set out in our licence referred to above.

Other than as permitted in any relevant BMJ Author's Self Archiving Policies, I confirm this Work has not been accepted for publication elsewhere, is not being considered for publication elsewhere and does not duplicate material already published. I confirm all authors consent to publication of this Work and authorise the granting of this licence.

For peer review only

Why India is struggling to feed their young children? A Qualitative analysis for tribal communities

Professor Monica Lakhanpaul; MBBS, DM, MPH, FRCPCH [1,2]

Susrita Roy; MA, MPhil[3]

Dr Lorna Benton; PhD [1]

Professor Marie-Carine Lall; MPhil, PhD [4]

Dr. Rajesh Khanna; MBBS, DNB, MSc [3]

Professor Virendra Kumar Vijay, PhD [5]

Sanjay Sharma; MSc, LLB [6]

Dr Logan Manikam, MBChB, FFPH, PhD [7,8]

Neha Santwani; MSc [3]

Dr Hanimi Reddy; MPhil, MSc, PhD [3]

Hemant Chaturvedi; MSc [9]

Shereen Allaham; PharmD, MPH [7,8]

Satya Prakash Pattanaik [6]

Tol Singh [9]

Pramod Pandya [9]

Priyanka Dang; MBA [3]

Dr Priti Parikh; MPhil, PhD [10]

Institutions

1. Population, Policy and Practice, UCL Great Ormond Street Institute of Child Health, University College London, London, WC1N 1EH, UK
2. Whittington Health NHS Trust, London, N19 5NF, UK
3. Save the Children, National Support Office, Gurugram, India
4. Institute of Education, University College London, UK
5. Indian Institute of Technology Delhi (IIT), India
6. Save the Children India, Rajasthan State Programme Office, Jaipur, India
7. Department of Epidemiology and Public Health, University College London Institute of Epidemiology and Health Care, London, UK.
8. Aceso Global Health Consultants Limited, London, UK.
9. Save the Children, Rajasthan, India
10. UCL Engineering for International Development Centre, Bartlett School of Construction and Project Management, The Bartlett, UCL Faculty of the Built Environment, London, UK

For peer review only - http://bmjopen.bmj.com/site/about/guidelines.xhtml

Corresponding author:

Monica Lakhanpaul,

Professor of Integrated Community Child Health

Population, Policy and Practice, UCL Great Ormond Street Institute of Child Health, 30 Guilford Street, University College London, London WC1N 1EH.

Email address: m.lakhanpaul@ucl.ac.uk

Word count: 8075

Acknowledgements:

PANChSHEEEL gratefully acknowledges the input from our community champions and field team, which made this project possible. We would like to thank MRC for funding the PANChSHEEEL project. We would also like to thank Isabel-Catherine Demel, a medical student who helped out in formatting the paper. The opinions in this paper solely reflect the views of the authors and not of organizations who have financially supported this research. All data used in this research is the property of the authors. The data that support the findings of this study are available from the corresponding author, Prof Monica Lakhanpaul, upon reasonable request. Prof Monica Lakhanpaul was supported by the National Institute for Health Research (NIHR) Collaboration for Leadership in Applied Health Research and Care (CLAHRC) North Thames at Bart's Health NHS Trust. This work is supported by the NIHR GOSH BRC. Dr Logan Manikam and Shereen Al Laham are funded via an NIHR Advanced Fellowship (Ref: NIHR300020).

For peer review only - http://bmjopen.bmj.com/site/about/guidelines.xhtml

Why India is struggling to feed their young children? A Qualitative analysis for tribal communities

Abstract

Objective: This interdisciplinary qualitative study aims to explore the health, education, engineering and environment (HEEE) factors impacting on feeding practices in rural India. The ultimate goal of the PANChSHEEEL project is to identify challenges and opportunities for improvement to subsequently develop socio-culturally appropriate, tailored, innovative interventions for the successful implementation of appropriate IYCF (Infant and Young Child Feeding) practices locally.

Design: Qualitative research method, involving 5 phases, (1) identification of local feeding practices; (2) identification of the local needs and opportunities for children aged 6-24 months; (3)-(5) analysis of the gathered qualitative data, intervention design, review and distribution.

Setting: Nine villages in two community development blocks, i.e. Ghatol and Kushalgarh, located in the Banswara district in Rajasthan, India.

Participants: 68 participants completed semi-structured interviews or focus group discussions including: mothers, grandmothers, Auxiliary Nurse Midwife, Anganwadi Worker, ASHA Sahyogini, school teachers, and local elected representative.

Phenomenon of Interest: IYCF practices and the factors associated with it.

Analysis: Thematic analysis.

Results: Our results could be broadly categorized into two domains: (1) The current practices of IYCF and (2) The key drivers and challenges of IYCF. We explicate the complex phenomena and emergent model focusing upon: mother's role and autonomy, knowledge and attitude towards feeding of young children, availability of services and resources that shape these practices set against the context of agriculture and livelihood patterns and its contribution to availability of food as well as on migration cycles thereby affecting the lives of 'left behind' and access to basic health, education and infrastructure services.

Conclusions: This interdisciplinary and participatory study explored determinants impacting feeding practices across political, village and household environments. These results shaped the process for co-creation of our context-specific intervention package.

Word count: 281

For peer review only - http://bmjopen.bmj.com/site/about/guidelines.xhtml

Keywords: Feeding practices; Children; Infant; Early Interventions**;** Participatory; Community.

Article Summary

Strengths and Limitations of this study

Strengths

- As an interdisciplinary study, the PANChSHEEEL (Participatory Approach for Nutrition in Children: Strengthening Health Education Engineering and Environment Linkages - HEEE) project explored a plethora of themes across the four domains of health, education, engineering and environment (HEEE) to evaluate contributing factors, current challenges and potential opportunities in Infant and Young Child Feeding (IYCF) practices in India. Using the qualitative data presented in this paper, the ultimate objective was to co-develop a context-specific multisectoral HEEE Nutrition intervention package that is socio-culturally appropriate, targeted and innovative to enhance IYCF practices 6-24 months aged children locally.
- This study employed a bottom-up approach, involving a close collaboration with the local affected population, interdisciplinary teams of researchers, non-government organizations (NGOs), as well as pre-existing and emergent research evidence in identifying current feeding practices, as well as perceived barriers and future opportunities for intervention.

Limitations

- The study was conducted on a relatively limited geographic scale, with data gathered merely across two community blocks in Rajasthan. Further research be conducted in neighboring rural areas in India will be needed to ensure the generalizability of the findings.

For peer review only - http://bmjopen.bmj.com/site/about/guidelines.xhtml

Introduction

The Global Nutrition Report (2018) reported that globally 150.8 million and 50.5 million children under the age of five years are stunted (inadequate height for age) and wasted (inadequate weight for height) respectively [1]. With 38.4% stunted children, India contributed a third of the world's burden for stunting in children under 5 years. With respect to wasting, India accounts for an even larger proportion, with 25.5 million children affected nationally – a number equivalent to half of the global burden of wasting [2]. Conscious of the fact that adequate nutrition enhances cumulative lifelong learning capacity and adult productivity, the Government of India (GOI) recognizes that improving nutrition will be of vital importance in poverty alleviation and the country's economic development in the long-run [3].

A variety of conceptual models have been used to explain the myriad and complex set of undernutrition determinants. One such model, proposed by the United Nations Children's Fund [4], illustrates the socio-ecological determinants influencing undernutrition. According to this model, inadequate dietary intake and infectious diseases are two key proximate factors for undernutrition alongside household food insecurity, inadequate care and feeding practices and unhealthy household environments.

Infant Young Child Feeding (IYCF) practices entail initiation of breastfeeding in the first hour of birth, exclusive breastfeeding till 6 months followed by complementary feeding after sixth months as well as optimal feeding practices essential for children until the age of two. Complementary feeding includes the introduction of age-appropriate semi-solid food alongside breast milk after six months of exclusive breast feeding [5]. Universal coverage of optimal breastfeeding can prevent 13% of global deaths in children less than five years of age, while appropriate complementary feeding practices could result in an additional 6% reduction in under-five mortality [6]. With 80-85% of brain growth taking place during the first two years of life, optimal IYCF plays an essential role also with respect to cognitive development, as reiterated by The Global Nutrition Report [7].

When comparing India's IYCF indicators with the global and regional indicators, two important phenomena become apparent: (i) India's breastfeeding indicators (early initiation 41.5%, exclusive breastfeeding 54.9%) are at par with the global and South Asian averages, however they are lower than that of its immediate neighbours Nepal (early initiation 54.9%, exclusive breastfeeding 65.2%) and Bangladesh (early initiation 50.8%, exclusive breastfeeding 55.3%) even though these countries have lower HDI score compared to India; and, (ii) India's complementary feeding indicators are lagging not only the global and South Asian averages, but also its neighbouring countries. While Minimum Dietary Diversity (MDD) in India (19.9%) is similar to the South Asian average, it is lower than that of the global average (29%) and

For peer review only - http://bmjopen.bmj.com/site/about/guidelines.xhtml

its neighbours Nepal (45%) and Bangladesh (26.6%). The major challenge is seen for Minimum Acceptable Diet (MAD) as the rate in India (MAD 9.6%) is lower not only than that of the global (18%) and regional average (12%), but also it is much lower than that of its neighbors Nepal (35.8%) and Bangladesh (22.8%) . [Ref no 8: UNICEF 2019]

Complementary feeding rates declined in India between the two National Family Health Surveys of 2005-06 and 2015-16 from 52.6% to 42.7%. The decline was seen in nearly all the regions and states but there was marked intra-country variation in the degree of decline. The highest decline was observed in the southern states (14% to 31%) which have comparatively better performing health systems in the country, while the northern states with weaker health systems had a lower degree of decline (8% to 10%). This could be attributed to the fact that the northern states already had much lower level of complementary feeding rate (during NFHS-3) compared to the southern states, so that any further decline becomes even more concerning. In the northern region, Rajasthan showed a decline of 9% in the complementary feeding rate. (supplementary file 1).

Disaggregated data from the NFHS 4 [9] points to Rajasthan state in the northern region recording one of the lowest IYCF indicators. While 28% children under age 3 years were breastfed within one hour of birth and 58% children under age 6 months exclusively breastfed, only 30% of children aged 6-8 months received complementary feeding along with breastmilk. About one-third (34%) of children age 6-23 months were fed the recommended minimum number of times per day, 9.7% had MDD and only 3.4% had access to MAD. The MAD score in Rajasthan is the lowest score among the larger states in India. Within the state, district Banswara which is a predominantly tribal area, has IYCF indicators worse than the state average and many of the other 32 districts. Table 1 shows the IYCF indicators in India, Rajasthan and Banswara districts.

Table 1: IYCF Indicators in India, Rajasthan and Banswara district, NFHS-4; 2015-16 (in %)

For peer review only - http://bmjopen.bmj.com/site/about/guidelines.xhtml

For peer review only

Indicators	India	Rajasthan	Banswara
Early initiation of breastfeeding	41.5	28.4	37.8
Exclusive breastfeeding under 6 months	54.9	58.2	57.1
Introduction of solid, semi-solid or soft foods (6-8) months	42.7	30.1	Not available
Complementary feeding – Minimum Acceptable Diet	9.6	3.4	0.8

Several Indian studies have sought to unravel some key aspects of India's malnutrition scenario, addressing important determinants at three levels: (i) household (maternal time constraint, dwindling family size, mother's age and education; lack of adequate knowledge; poor uptake of existing nutritional services; child targeted market with wide availability and consumption of ready-to-eat marketed food items); (ii) community (social and economic context; feminization of agriculture; fragile food security/seasonal food paucity due to less focus on food crops and vegetables; dwindling livestock – especially milk producing animals; low connectivity to remote locations; migration; exposure to media); and, (iii) government (inadequate and unresponsive Integrated Child Development Scheme [ICDS] and health care system; paucity of technical knowledge among service providers regarding IYCF). Several studies over the last decade have examined varying associations between household environmental characteristics and stunting in under-five children, highlighting the need for interdisciplinary research [9] [10-15].

Our study, the Participatory Approach for Nutrition in Children: Strengthening Health, Education, Environment and Engineering Linkage (PANChSHEEEL) funded by the Medical Research Council (UK) was designed to: (i) explore health, education, engineering and environment (HEEE) factors that influenced Infant and Young Child Feeding (IYCF) practices and (ii) develop a socio-culturally appropriate, tailored, innovative and integrated cross sector HEEE package to support optimal IYCF practices. This is central to the convergent action planning process of India's National Nutrition Mission or the POSHAN Abhiyaan (Prime Minister's Overarching Scheme for Holistic Nutrition) that has IYCF as the first target to be monitored.

Conceptual framework

Drawing upon the review of the above studies, the principal investigator and the co-investigators synthesized the determinants from the literature review to frame the HEEE conceptual framework as a

For peer review only - http://bmjopen.bmj.com/site/about/guidelines.xhtml

socio-ecological model (Figure 1). This framework sought to synthesize the complex interplay of factors across three environments: political, village and household; and the inter-linkages between institutions, initiatives (schools, health services, ICDS and public distribution system) and communities. The qualitative studies that were reviewed focused on undernutrition or stunting and not specifically on IYCF and child feeding practices that have assumed crisis proportions but have received scanty attention among public health nutrition researchers in India.

Figure 1: HEEE Conceptual Framework

The formative phase of the PANChSHEEEL study was thus framed to develop an understanding of a deep-dive interplay of the determinants of IYCF to inform the co-designing of an intervention model that can address this crisis in such contexts.

This paper presents the qualitative findings of the PANChSHEEEL study; that aims to identify and document local community IYCF practices including water, sanitation & hygiene (WASH) and energy practices, to identify local challenges, drivers, resources, opportunities and needs for IYCF in 6-24 month old children at individual, household, community and environmental level; and, to map the linkages between identified opportunities and challenges in order to determine how the needs identified can be addressed.

Methodology

The larger PANCHShEEEL study used both qualitative and quantitative research methods, triangulating the two to synthesize evidence. This paper presents the qualitative component of the formative study, in line with COREQ guidelines.

Study setting

The study was conducted in nine villages in two community development blocks (herein after, blocks) of Banswara district in Rajasthan, India. These two blocks, Ghatol and Kushalgarh, were purposively chosen to represent district diversity. The Ghatol Block is located in the 'command area' of the Mahi River Dam; all villages are irrigated by canals and have multiple crops yearly. In contrast, Kushalgarh is dry and semi-

For peer review only - http://bmjopen.bmj.com/site/about/guidelines.xhtml

arid with poor irrigation; consequently, most villages are mono-crop areas. The village selection process is represented in the following consort diagram (Figure 2).

Figure 2: Consort Diagram for selection of Study Villages

Patients and Public involvement and Engagement

In line with PANChSHEEL participatory approach, considerable efforts were placed on Patient and Public Involvement and Engagement (PPIE). Suitable individuals and representatives from both the Ghatol and Kushalgarh Block were identified and subsequently engaged in all steps of the study, i.e. protocol development, study design, results dissemination.

The Core Research Team was further supported by Community Researches from the two study blocks who aided in steering the project phases locally. With the input from 'Save the Children' Rajasthan, two well-suited individuals were identified who had been proactive collaborators in previous projects by 'Save the Children' India. They were mainly involved in selecting suitable candidates who could engage actively in project activities, as well as coordinating with the Community Champions (CCs), who were crucial in establishing networks with potential study participants among Community Members (CMs).

Relevant stakeholders were identified and selected in a stakeholder mapping workshop as part of the formative study phase. Through snowballing, i.e. a recruitment method where existing participants are involved in the identification and recruitment of future participants using their social networks, the research team remained open to potential new stakeholder(s) identified during the data collection process. The recruitment of study participants was based on willingness to participate and availability.

Respondent selection

The respondents included stakeholders who play a critical role in feeding and caring for young children. There are three cadres of frontline workers at the village level in Rajasthan: (i) the Auxiliary Nurse Midwife (ANM), the multi-purpose female health worker; (ii) the Anganwadi Worker (AWW), the cadre of the Integrated Child Health Services (ICDS) who runs the Anganwadi Centre (AWC) at each village; and (iii) the ASHA Sahyogini (AS), the community health worker (ASHA in other states). One ANM was selected from each of the sub-centers in the study villages; in case of sub-centers with 2 ANMs, the senior ANM was selected. Owing to the size of the village generally one AWW or AS are chosen bit in the case where the village has more than one AWW or AS, then both shall be interviewed as they represent different geographic and social strata. One school from each village was chosen and the principal/head or the most

For peer review only - http://bmjopen.bmj.com/site/about/guidelines.xhtml

senior teacher was interviewed. The ward or local Sarpanch of each village was also interviewed. At household level, mothers and grandmothers who were literate and conversational (with at least one 6–24-month-old child) were identified and selected based on their availability and willingness to participate either during the household survey or by frontline health workers.

Data collection

Key Informant Interviews (KIIs) and Focus Group Discussions (FGDs) were used for collecting primary data. Semi-structured open-ended interview guides were used to understand respondent knowledge and feeding practices perceptions. FGDs were conducted with mothers and grandmothers. The date, time and meeting place were decided in consultation with AWW and AS. FGD guides included topics on maternal time use, household care giving roles, breastfeeding and complementary feeding, hygiene, play and communication. An approximate of 5-9 questions were asked under each topic and the data collected was classified into 4 major themes (Health and Nutrition, Education, WASH and Cooking Fuels). Participants were encouraged to share their frank opinions, irrespective of the views of others in the group, in order to elicit insights about IYCF issues (Table 2).

FGDs were conducted by community researchers from the same blocks or adjoining block who were able to communicate fluently in the local dialect, *Wagdi.* They were trained on FGD techniques by the principal investigator and co-investigators which included principles and methods of qualitative research with a special focus on interview and group discussions, and an outline of data analysis. Hands-on support was provided by other team members who were well versed with the research methods and local contexts. The duration of the interviews ranged between 25-40 minutes and the FGDs ranged between 45-75 minutes.
We relied on a hybrid data saturation approach: achieving both: (i) a priori thematic saturation (the degree to which identified codes or themes are exemplified in the data) that informed sampling strategy and (i) inductive thematic saturation (the emergence of new codes or themes) that informed data analysis [16].
Field notes based on observations played a crucial role in data collection and analysis. Digital recorders were used to record all KIIs and FGDs. Written consent was taken from key informants whilst for FGDs verbal consent was sought and formally recorded. Audio data was then transcribed (from local language to Hindi) and translated (from Hindi to English) with back translations done for quality assurance. Both audio data and text were anonymised to maintain confidentiality and securely stored. A pilot round was conducted in January 2018 following which formal data collection continued for four months. Caution was maintained regarding selection bias of FGD participants due to the active role of frontline health workers in service delivery. FGDs were organized at Integrated Child Development Services (ICDS) or Anganwadi Centers

For peer review only - http://bmjopen.bmj.com/site/about/guidelines.xhtml

(AWC) centers at each village with care taken to ensure that residents residing far from AWCs were not excluded. This study was conducted according to the guidelines laid down in the Declaration of Helsinki and all procedures involving research study participants were approved by the UCL ethics [Ethics ID 4032/002] and Sigma IRB [10025/IRB/D/17-18].

Topic Guide

Through the means of a literature review, expert advice and pilot testing with different population subsets, a thematic guide was designed prior to the qualitative data collection process. This guide facilitated the conductance of the KIIs and FDGs, allowing the topic of IYCF to be explored across the four major dimensions (1) Health and Nutrition, (2) Education, (3) Water, Sanitation and Hygiene and (4) Energy to capture current practices, barriers and possible opportunities to foster positive change across all four areas.

Data processing and analysis

The research team undertook daily meetings to reflect upon their key observations and data collected from field notes. Data was transcribed and translated to bring out common major themes based on commonality and differences. Data generated from all sources were subjected to triangulation of mixed qualitative and quantitative methodology [17] and linked thematically for key findings. The qualitative data analysis software, IQDAS was used for the data analysis process. The data was coded and analyzed jointly by the manuscript authors (co-investigators) to ensure consistency and reliability in interpretation. Codes were analyzed across axial and selective codes across respondent categories and sites (villages and blocks). Triangulation was performed across methods and respondents. Results from KIIs and FGDs were compared and assessed for similarities and differences across respondent categories. Findings from villages within a block conveyed similar interpretations and hence reinforced data validity. The investigator group reached a consensus to merge village and block level findings, except in those themes or sub-themes where they presented novel findings. Data analysis was first completed independently by interviewers followed by a series of joint sessions between interviewers and co-investigators.

Respondent validation

A series of meetings were conducted with representatives from all respondent categories from the same villages to validate the data. Within the qualitative data sets, all steps of a thematic analysis, i.e. (1)

For peer review only - http://bmjopen.bmj.com/site/about/guidelines.xhtml

identifying, (2) analyzing and (3) interpreting patterns of meaning, i.e. themes, were employed. Respondent validation was facilitated by Community Researchers in September 2018 and closely overseen by local Save the Children staff. This enabled us to refine the emergent themes and model to its final shape.

Quality assurance

The inter-disciplinary team comprised of experts in public health nutrition, epidemiology, social sciences, paediatrics, education and civil and environmental engineering. A dedicated team of co-investigators supervised training, data collection, transcription and translation, data management and analysis. Field teams were oriented with the study protocol, tools and research techniques, including hands-on exercises, before actual data collection began.

Results

A total of 68 participants were recruited (table 2). The information was obtained from FGDs with 17 mothers and paternal grandmothers and 51 key respondents across 9 villages. More than half of the mothers were aged between 18 to 26 years and majority of them (approximately 92%) had either one or two children under 5 years in their household. 61% of the women including both mothers and grandmothers were illiterate. Of the rest who attended formal schooling, there was roughly a 10% dropout rate because they got married early or participation in household work. More than 90% of the women interviewed were involved in the agriculture industry either in the form of farming or livestock rearing. Based on the present study that defines time spent in tending to farming and livestock as 'market work', women spent a greater amount of time in activities of farming and tending livestock.

Table 2: Respondents Categories and Methods

Category of Respondent	Method Used	Numbers
Mothers and Grandmothers	FGD	17
ANM	KII	7
AWW	KII	13
ASHA Sahyogini	KII	13
School Teacher	KII	9
Local elected representative	KII	9

For peer review only - http://bmjopen.bmj.com/site/about/guidelines.xhtml

Deriving from the study objectives, this section is divided into two parts. The first section provides an in-depth description of IYCF practices. The second section will explain the factors (drivers and challenges) associated with it. Online supplementary file 2 illustrates the themes identified and example quotes from focus groups and interviews with respondents. Figure 3 illustrates the current practices of IYCF.

IYCF practices

Figure 3. Infant and Young Child Feeding (IYCF) – Current Practices

✶ *Core theme 1: Partial breastfeeding is a common practice in first six months.*

Most mothers and grandmothers reported that 90% of the births take place in hospitals due to the presence of trained staff who are equipped to handle medical emergencies and breastfeeding was initiated within the first two to three hours of childbirth in hospital. Of the few women who had given birth at home, also confirmed that breastfeeding starts as soon after birth as possible. Delay in breastfeeding was reported mainly due to "*secretion of milk not starting*." [Grandmothers FGD, Kushalgarh].

"If the baby is delivered at home breastfeeding is starts immediately after birth."
[AS, Kushalgarh]

Giving colostrum was considered mandatory among a majority of respondents, with only a minority of grandmothers in Kushalgarh expressing negative views about colostrum as "dirty milk". All respondents denied giving any pre-lacteals like honey to the children though these practices were reportedly followed in cases of home births, albeit very few.

Most mothers in Ghatol reported that they exclusively breastfed for four to six months. In Kushalgarh, a majority of respondents breastfed till the third month. Breastfeeding frequency in the first six months was reported to be 'on cue', as and when the child cried.

"Mothers of children who are on breast milk work inside the village,
so they can be summoned home whenever the child cries"
[Mothers, Ghatol]

For peer review only - http://bmjopen.bmj.com/site/about/guidelines.xhtml

Mothers who resumed their agricultural work after three months reported coming home from work every two hours for breastfeeding. On further prompting it was revealed that, at times, water was given to children below six months, especially during summer months due to the belief that the child gets thirsty. Dissolving biscuits in milk or water and feeding it to children in case they cried or the mother was not available appeared to occur on occasion, thus compromising exclusive breastfeeding. This practice was also promoted by Frontline Health Workers (FHWs). Animal milk, especially goat milk, was given when the mother was unavailable or when the mother's milk amount was perceived to be inadequate.

"*In the summer season the child becomes thirsty, so water is given to the child. We also recommend giving water sometimes*."

[AWW, Ghatol]

✶ *Core theme 2: Biscuits given as complementary food after six months*

Consumption of biscuits as convenience foods increased after six months in both blocks. Soaked in water or tea, they were used as a semi-solid food for local children. Two reasons were cited for this practice: (i) easy availability and low costs (5 INR or 0.7 USD per packet) and, (ii) children liked the sweet taste; hence making it easy to feed.

"Children like biscuits and it is easy to feed them."

[Grandmothers FGD, Kushalgarh]

"*This is the cheapest option and is easily available in the village*."

[Mothers FGD, Ghatol]

These were given in the morning as the first meal of the day, and/or between meals when children cried. FHWs commonly approved of this practice.

"*We tell the mothers that they can give biscuits to the child, at least it will keep them full*."

[ANM, Kushalgarh]

For peer review only - http://bmjopen.bmj.com/site/about/guidelines.xhtml

Biscuits were often employed as a method to *pacify* the child when the mother or other care givers were busy in their daily chores. The number of biscuits given per day varied from five to ten per day.

✶ *Core theme 3: Dietary preferences and recent shifts*

On average, complementary feeding was commenced around 7-8months. The main reason of delayed complementary feeding was the child's "lack of interest" in eating semi-solid foods, marked by prolonged feeding time, crying and also fidgeting while eating. A majority of participants reported to be confident regarding appropriate complementary feeding.

"*The baby starts drinking milk (dairy) after 6 months. We know what to give and what not to.*"
[Mothers FGD, Ghatol]

The foods included pulses (in watery consistency), *khichdi* (savoury rice and pulse gruel), *dalia* (porridge with ground wheat with milk or water, mostly sweet) or small pieces of roti dipped in milk. The use of commercial baby food was nominal. FHWs reported that children were fed four to five times a day on average; mothers and grandmothers, however, reported demand feeding to be common practice, i.e. feeding when the children cried.

"*Whenever the children get hungry we feed them.*"
[Mothers FGD, Kushalgarh]

Most mothers from both blocks reported that, because children ate in small quantities, it was difficult to cook separately for them every day. Neither FHWs nor the mothers had clarity about the frequency and quantity to be fed to children. Portion size varied depending on the child's "interest". All mothers confirmed that they received packets of Take-Home Ration (THR) regularly, but had little knowledge of the proper recipe.

Consumption of non-vegetarian food was dwindling while those who consumed animal products, e.g. meat and eggs, did so discretely. This was attributed to religious reasons, locally termed the '*bhagat*' (disciple) culture.

For peer review only - http://bmjopen.bmj.com/site/about/guidelines.xhtml

"This is a recent trend. Hawan (worshipping with holy fire) was conducted in the village after which every family has stopped eating. No question of giving it to children."

[Ward Panch, Ghatol]

This practice was more prevalent in Ghatol, where more respondents ascribed to these beliefs.

In Kushalgarh, hens were reared in most households, consequently resulting in a higher availability and consumption of non-vegetarian foods. However, due to religious values, respondents often denied its consumption. .

"This is made only if there is a guest at home or there is any occasion. Chicken is mostly eaten (on such occasions)."

[Teacher, Kushalgarh]

Responses from both blocks confirmed that children under five years were rarely given non-vegetarian food, and if so, only in small quantities.

Consumption of fruits, though unanimously considered to be beneficial, was rare on account of availability and cost. Fruits are generally available in the markets located far away from the villages; more so in Kushalgarh. Purchase was possible only when a family member visited these markets.

"*Fruits are not very common in daily diet. Only when the parents go to the market they get fruits."*

[Teacher, Kushalgarh]

Vegetables consumption also comprised "*potatoes, tomatoes and onions almost daily*." [ANM, Ghatol] There was less consumption of green leafy vegetables, for children, even though it was readily available.

Key Drivers and Challenges

IYCF practices described in the previous sections are attributable to contextual issues emerging at the household, community and government level. Differences evident between Ghatol and Kushalgarh include cropping patterns, market access and fruit and vegetable availability. These differences aside, there were similarities in the drivers of IYCF practices between these two blocks. Figure 4 presents the connecting subthemes of the key drivers and challenges of IYCF practices.

For peer review only - http://bmjopen.bmj.com/site/about/guidelines.xhtml

Figure 4. Infant and Young Child Feeding (IYCF) – Key Drivers and Challenges: Connecting subthemes.

✶ *Core theme 4: Time-constrained mother and role of other family members*

Time constraints amongst mothers emerged as a critical determinant accounting for lack of exclusive breastfeeding (infants below six months) and feeding (home cooked food) at regular intervals (to older children).

"*The mother cannot sit at home taking care of the child. Who will look after the animals, get water or cook? They also have to do agricultural work.*"
[Grandmothers FGD, Kushalgarh]

All mothers, while being primary caregivers, reported that they were guided and influenced by grandmothers, other family members and FHWs. Mothers stayed at or worked in close proximity to their homes until the child was about two months old. During this time, they continued with cooking and other household chores, as well as outdoor work, i.e. firewood and water collection. Mothers from Ghatol reported that they did not go out for work outside the villages. Occasionally, mothers took up daily work in nearby factories, but only once their child was at least one year of age. Women in Kushalgarh migrated to nearby towns and districts as a family, once children were above six months.

"*When my daughter-in-law works outside, then we grandmothers take care of the children.*"
[Grandmothers FGD, Ghatol]

Teachers reported that also elder siblings, especially girls, were often absent from school to take care of younger siblings, especially in families where both parents had migrated (seasonally) or if the primary care giver was unavailable.

✶ *Core theme 5: Livelihood challenges in a chronic poverty setting*

Agriculture is the main source of livelihood and revenue for families in both blocks, despite clear differences in irrigation facilities and cropping pattern in Ghatol and Kushalgarh.

For peer review only - http://bmjopen.bmj.com/site/about/guidelines.xhtml

> "*Most people here are in agriculture. Both males and females of all families take part in agriculture.*
> [School Teacher, Kushalgarh]

Women in all study villages were engaged in agriculture and livestock farming throughout the year; men took part in agriculture only during sowing and harvesting seasons. Some women were engaged under the National Rural Employment Guarantee Scheme though employing mothers with children aged less than two years was not common practice. Local wage labor was reported to be a common source of income for men residing in Ghatol villages due to the proximity to the district town and a cloth mill. Circular migration to urban areas of the adjacent states of Madhya Pradesh and Gujarat was common among men in Kushalgarh.

> "*Some men from the village work in Mayur mills (textile mill near Banswara). Some also work as wage labour, but only during lean seasons.*" [School Teacher, Ghatol]

✶ *Core theme 6: Childcare services fails to deliver on most counts*

Proactive steps are being taken by government programs to address IYCF related challenges. Breastfeeding information was mostly provided at childbirth facilities and, these practices were much better compared to complementary feeding. The overwhelming evidence from all the villages was that level of knowledge and counseling skills were poor among AWW and AS. They were not aware of malnutrition indicators and appropriate complementary foods, often promoting the introduction of animal milk, semi-solid or solid food into a baby's diet before the age of six months. Infant weight was recorded in some cases.

> *"We take weight, but currently the weighing machine is not working."*
> [AWW, Kushalgarh]

In many villages, several (non-health) key informants reported that AWWs mostly catered to community needs staying close to the AWC. The supervisory cadres rarely visited the villages.

> "*Neither LS (Lady Supervisor) nor the CDPO (Child Development Project Officer) visits our centre. We only go to the sector meetings where they ask us about the number of pregnant women registered, children born and packets of THR distributed. "*
> [AWW, Kushalgarh]

For peer review only - http://bmjopen.bmj.com/site/about/guidelines.xhtml

Ghatol received more support from the district level than Kushalgarh owing to its proximity to the headquarters; this was further complemented by the presence of NGOs and other civil society actors in Ghatol.

✶ *Core theme 7: Emerging role of schools and panchayats in health and nutrition programs*

School teachers were an important resource for local communities; the schools functioned regularly and efficiently with teachers attending school daily. Mid-day meals were provided. With male members mostly working outside the village (either daily wage or migration), parents' participation in these meetings were occasional. According to school teachers (mostly male), fathers interacted with them with few mothers participating. The state has recently formulated at policy for co-location of AWC at school premises.

"*The AWC is in the school premises; we can always keep a check whether children are getting their meals. The AWW and the other staff also come in time and regularly open the centre*."

[School Teacher, Kushalgarh]

Many teachers reported taking a personal interest in visiting and monitoring the functioning of AWCs; however due to their own busy schedule and excessive workload (in part due to staff shortage), they were not always proactive.

"*We are already overburdened with the work of the school. It is not possible to check the functioning of the AWC.*"

[School Teacher, Ghatol]

All the ward panches (elected Panchayat [local self-government] member) were unaware of the Village Health Sanitation and Nutrition Committees (VHSNC) and played no role in this critical village level platform for promoting health and nutrition of mothers and children.

✶ *Core theme 8: WASH practices can play a vital role*

Caregivers were unanimous in their perception that illnesses such as diarrhea, fever or cough represented one contributing factor of inappropriate nutrition among children. There was little awareness that these diseases may be partially linked to WASH deficiencies. Hand pumps were the only source of drinking water in both blocks. Unlike in Ghatol where a majority of hand pumps have water throughout the year, some sources in Kushalgarh dry up "*due to scarcity of water*" [ANM, Kushalgarh] during the summer months. The only measure for drinking water treatment was using a cloth for filtering the water while filling; this

For peer review only - http://bmjopen.bmj.com/site/about/guidelines.xhtml

practice (as reported by the FHWs) was more common in Kushalgarh than in Ghatol. In several villages of Kushalgarh, a local reverse osmosis plant using solar panels was set up under a new scheme by the Public Health and Engineering department.

Mothers, grandmothers and other key informants reported that communities in Ghatol were more willing to construct sanitary facilities than those in Kushalgarh, even though there is a national sanitation program which incentivizes toilet construction at household level.

"Those who constructed the toilets have not got their due payments. I have been following up with the Sarpanch's office regularly, but there is no response. This discourages those who have not yet constructed."

[Ward Panch, Kushalgarh]

In both blocks, however, a majority of households had access to functional toilets.

"The government has constructed toilets in every household. Whatever facilities the government has provided us, I have provided in this village."

[Ward Panch, Ghatol]

Most members reported using toilets, with the exception of a few elders who prefer open defecation. None of the under-two children defecated in toilets. Small children defecated on pieces of cloth which were subsequently disposed of in open fields near the house. The older children defecated in the open, near their homes, except some in Ghatol who were reported to use toilets.

Awareness on the importance of hand washing was high in all study villages. Hand washing with water and soap before cooking and eating was unanimously reported in Ghatol, but less so in Kushalgarh due to water scarcity.

Discussion

IYCF practices can be categorized as optimal and suboptimal. Optimal practices such as early breastfeeding initiation, reduction in pre-lacteal use, mothers being encouraged to exclusively breastfeed the child (till six months) and continue breastfeeding till two years were reported in our study villages. Suboptimal practices were more widespread and included giving water in the first six months, feeding biscuits from the fourth

For peer review only - http://bmjopen.bmj.com/site/about/guidelines.xhtml

month and complementary feeds lacking in quantity, frequency and diversity. The emergent model bears out the interactions of the socio-ecological elements framed in our conceptual framework (Figure 5).

Figure 5: Emergent Model

It is in this backdrop that we sought to identify factors that influence and shape these existing practices and then design interventions that are able to address barriers and promote improvement. The evidence presented in this paper forms the basis of the socio-culturally appropriate, tailored, integrated and interdisciplinary interventions developed as part of the PANCHSHEEL project drawing on the sectors of: (i) health and nutrition, (ii) education and (iii) engineering and environment.

Most of the facilitators and barriers were in the realm of knowledge and skills. The two most common barriers across most household were lack of appropriate knowledge of mothers and other caregivers about: (i) complementary feeding practices (frequency, quantity and quality) and, (ii) recipe for cooking Take Home Ration (supplementary nutrition) distributed by the Anganwadi Centre. The knowledge gaps also resonated with incomplete information that was provided by the FHWs; this was also one of the barriers to promoting positive IYCF practices. Two such (highly prevalent) practices were giving water to babies less than 6 months and feeding biscuits. We also noted the divergence between perceptions of mothers and grandmothers. This was largely on account of mothers caring and feeding the children more actively in contrast to grandmothers who looked after children largely when mothers were away for work and were not often the final decision makers of care and feeding practices.

Resource constraints mothers emerged as another significant factor. Lack of mother's time to continue exclusive breastfeeding as well as dedicating time for separate cooking and feeding was one of the commonest barriers to positive IYCF practice. As male members travelled to nearby towns for daily wage labor or migrated to nearby states, (owing to lack of employment opportunities in the villages), women were compelled to dedicate more time to agricultural work. The poor environmental conditions and limited access to basic water and sanitation services also contributed to the time and resource constraints faced by women.

Little or no availability of animal milk resulted in children between 6 months to 2 years hardly receiving any milk in their diet. Limited use of vegetables and fruits was due lack of markets, inadequate resources to cultivate and high cost. As a result, children's diets were seldom rich in iron and vitamins. The absence of adequate resources was not merely a household level issue, but also rooted in the local cultural and religious contexts. The remaining two determinant categories; motivation and attitude, were mostly linked to either knowledge or resources.

For peer review only - http://bmjopen.bmj.com/site/about/guidelines.xhtml

Delineation of these factors helped in identifying key 'actors' who could bring about improvement in IYCF practices. To ensure continued support to mothers and other caregivers on positive IYCF practices, the knowledge and skills of FHWs, especially ASHA Sahyogini and Anganwadi Workers, need to be upgraded. Finally, there is a need for garnering community support for these children for which the role of important members like school teachers and elected representative was also identified as crucial.

This study was also subjected to certain logistical limitations and research related concerns. The language was a barrier to be addressed, everyone knew Hindi, but only two people in the team were versed with Wagdi, the local dialect. This resulted in challenges for translation and transcription of the data from the discussions. Moreover, the availability of the functionaries and the key stakeholders including the teachers at the schools, local official representatives were limited. This also added to the time constraint. All data collected was based on the observation and interactions held in a limited time of 2 hours per village. Sometimes the recall of information was difficult for participants, this was seen when the exact dates of births could not be retrieved resulting in calculation of the child's age based on estimated information on their month and year of birth. Besides, there were challenges to encourage women to participate in the interviews. They either did not feel empowered to contribute or were busy with household chores. For the same reason, availability of mothers was limited considering their demand for domestic work is a lot more. To encourage them there were multiple attempts to organize FGDs and KIIs. The researchers had to spend a significant amount of time with the caregivers to build the space where some of them could freely and confidently contribute to the discussion. On top of this, the decision to include mothers who have children at the age of less than 2 years old led to a varied number of eligible mothers from 35 households to 68 households in some villages, which was not align with the initial plan to cover 50 households per village. Lastly, there might be a selection bias of the participants of the FGDs due to the active role of AWW and ASHA workers in the selection of mothers which implied that only those with whom they have a good rapport would be identified. The prior training about the process could also influence the information given by some of the mothers. Another reason for the selection bias could also be that most of the FDGs were held in the AWC, as a result of which only the residents near the AWC could take part excluding the ones that are far away. We acknowledged the limitations and aimed to find ways to overcome the challenges we faced.

This study, and the urgent need for IYCF interventions, still holds a significance in the light of the recently released Global Hunger Index 2019 data [18]. India's Global Hunger Index (GHI) score declined from 38.8

For peer review only

For peer review only - http://bmjopen.bmj.com/site/about/guidelines.xhtml

in 2000 (rank: 83 out of 113 countries) to 30.3 in 2019 (rank: 102 out of 117 countries), placing the country in the 'serious' category. Achieving internationally agreed targets on stunting and wasting in under-five children is key to achieving Sustainable Development Goals. The complex set of factors and barriers to appropriate IYCF practices – captured in the HEEE conceptual framework and the qualitative data – pose a challenge in achieving the SDGs. While the Government of India has designed and implemented several programs to address these barriers, they reported limited success primarily because of the nature of isolated interventions as opposed to much needed multifaceted approach. The task at hand is therefore a well-designed, locally feasible, multi-sectoral interventions across health and nutrition, education as well as water and sanitation. Through this study we demonstrate the need for integrated and cross-sectoral research to comprehensively shape the feeding practices of children; thereby creating a scope for coordinating the ongoing interventions in a manner to achieve the desired target (the subject of another paper).

Ethics approval and consent to participate: The PANChSHEEEL Project, including qualitative and quantitative data collection methods as well as the tools obtained ethical approval from the UCL ethics committee (REF NO: 4032/002) in United Kingdom and the Sigma-IRB (REF NO: 10025/IRB/D/17-18 REF NO: 4032/002) in India.

Declaration of Conflicting Interest:

The authors declared no potential conflicts of interest with respect to the research, authorship and/or publication of this paper.

Financial Support and Funding:

This work was also supported by the Global Challenges Research Fund and funded by the MRC, AHRC, BBSRC, ESRC and NERC [grant number: MR/P024114/1].

Author Contributions:

ML, ML, PP, RK, and SS conceived the original concept of the study and designed the research methodology. SR, ML, PP, RK, HC, SS, SPP, TS, HR, and PP carried out the interviews, analysed the data, and wrote the paper. ML, ML, PP, SR, HC, NS, SS, SPP, LB, VV, PD and RK validate the study and revised the manuscript critically for important intellectual content. SA contributed to the manuscript writing, edited the final manuscript and prepared for submission. ML had primary responsibility for the

For peer review only - http://bmjopen.bmj.com/site/about/guidelines.xhtml

final content. All authors read and contributed to the reviewing the analysis of the data, the designing of the manuscript, and the approval of the final manuscript.

Data Statement:

The data of this study is available from the corresponding author upon reasonable request.

For peer review only

References

1. Fanzo J, Hawkes C, Udomkesmalee E, et al. (2018) Global Nutrition Report: Shining a light to spur action on nutrition.
2. UNICEF-WHO-World Bank: Joint Child Malnutrition Estimates 2018. Available From: https://data.unicef.org/resources/levels-and-trends-in-child-malnutrition-2018/
3. Niti Aayog. (2017). National Nutrition Strategy. New Delhi; India: Niti Ayog, Government of India. Available from: http://niti.gov.in/writereaddata/files/document_publication/Nutrition_Strategy_Booklet.pdf.
4. UNICEF. (2017). Forest Lanterns. New Delhi: Penguin Random House.
5. World Health Organization. Infant and young child feeding: model chapter for textbooks for medical students and allied health professionals. Available from: https://www.who.int/nutrition/publications/infantfeeding/9789241597494.pdf
6. Jones AD, Ickes SB, Smith LE, et al. (2013) World Health Organization infant and young child feeding indicators and their associations with child growth: a synthesis of recent findings.
7. Gupta A, Thakur N. Infant and young feeding practices in india: current status and progress towards SDG targets. Proceedings of the Indian National Science Academy. (2018) Nov 13;84(4):853-65.
8. UNICEF. (2019). Global UNICEF Global Databases: Infant and Young Child Feeding: Minimum acceptable diet, Minimum diet diversity, Minimum meal frequency. New York; USA: UNICEF. Available from: https://www.unicef.org/nutrition/files/IYCF_programming_guide_Apr_2013.pdf
9. International Institute of Population Sciences. (2017). National Family Health Survey, India: Key Findings from NFHS-4 (2015-16). Mumbai, India: International Institute of Population Sciences. Available from: https://dhsprogram.com/pubs/pdf/FR339/FR339.pdf
10. Imran M, Ilyas N, Mahmood T. Infant and young child feeding (IYCF) practices. Rawal Medical Journal. 2017 Oct 1;42(4):546-9.
11. Chaturvedi S, Ramji S, Arora NK, Rewal S, Dasgupta R, Deshmukh V. Time-constrained mother and expanding market: emerging model of under-nutrition in India. BMC public health. 2016 Dec;16(1):632.
12. Dasgupta R, Arora NK, Ramji S, Chaturvedi S, Rewal S, Suresh K, Deshmukh V, Thakur N. Managing Childhood Under-Nutrition: Role and Scope of Health Services. Economic and Political Weekly. 2012 Apr 21:15-9.
13. Ramji S. Impact of infant & young child feeding & caring practices on nutritional status & health. Indian J Med Res. 2009 Nov 1;130(5):624-6.

For peer review only - http://bmjopen.bmj.com/site/about/guidelines.xhtml

14. Desai S, Banerji M. Negotiated identities: Male migration and left-behind wives in India. Journal of Population Research. 2008 Oct 1;25(3):337-55.
15. Lee C, Lakhanpaul M, Maza Stern B, Parikh P. Associations between the household environment and stunted child growth in rural India: A cross-sectional analysis. UCL Open: Environment. 2019 Mar 6.
16. Saunders B, Sim J, Kingstone T, Baker S, Waterfield J, Bartlam B, Burroughs H, Jinks C. Saturation in qualitative research: exploring its conceptualization and operationalization. Quality & quantity. 2018 Jul 1;52(4):1893-907.
17. Halcomb, E.J. and Hickman, L., 2015. Mixed methods research.
18. Welt Hunger Hilfe. (2019). Global Hunger Index: The Challenge of Hunger and Climate Change. Dublin, Ireland: Welt Hunger Hilfe. Available from: https://www.globalhungerindex.org/pdf/en/2019.pdf
19. Dasgupta R, Chaand I, Barla KR. The slippery slope of child feeding practices in India. Indian pediatrics. 2018 Apr 1;55(4):284-6.

For peer review only

For peer review only - http://bmjopen.bmj.com/site/about/guidelines.xhtml

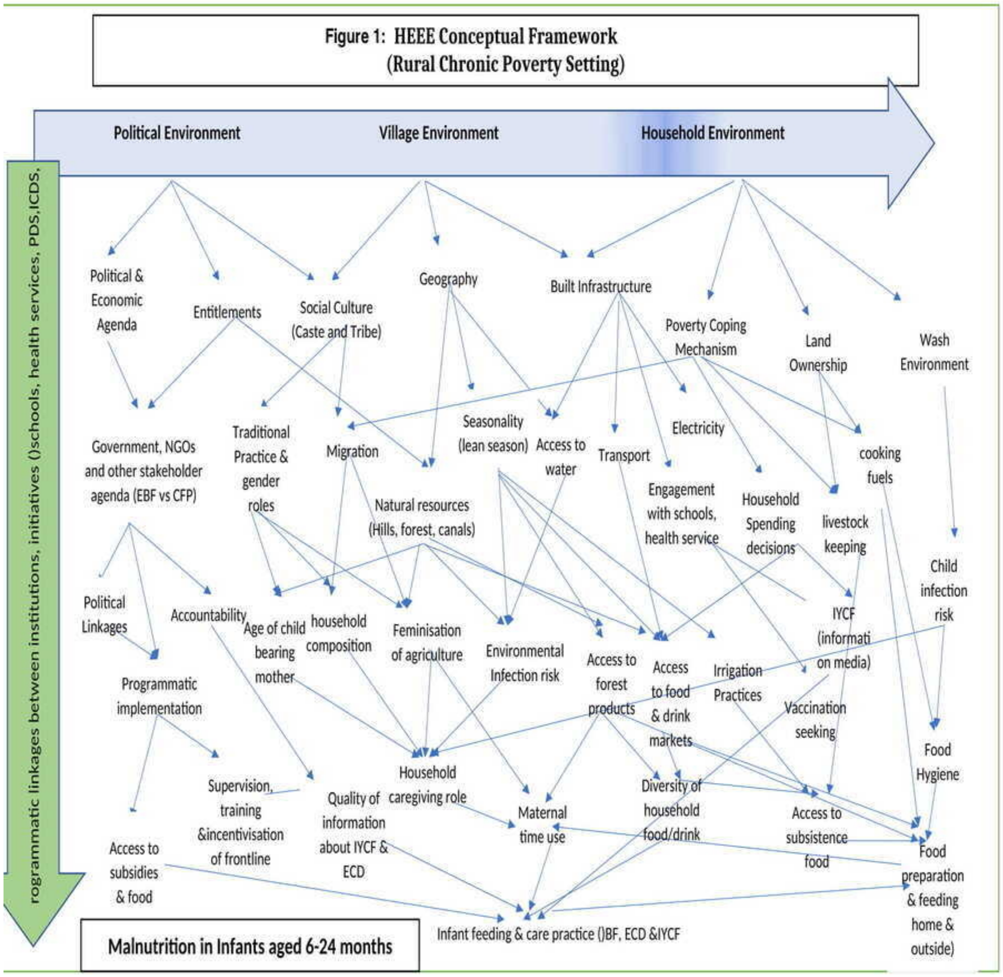

HEEE Conceptual Framework

76x76mm (300 x 300 DPI)

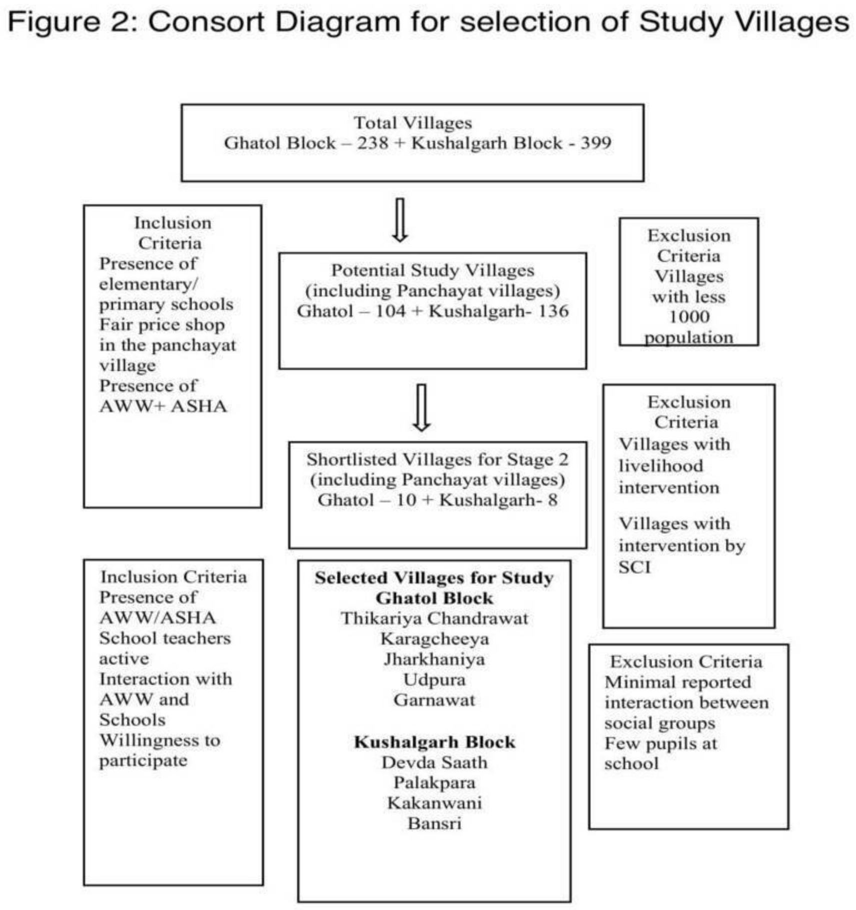

Consort Diagram for selection of Study Villages

76x76mm (300 x 300 DPI)

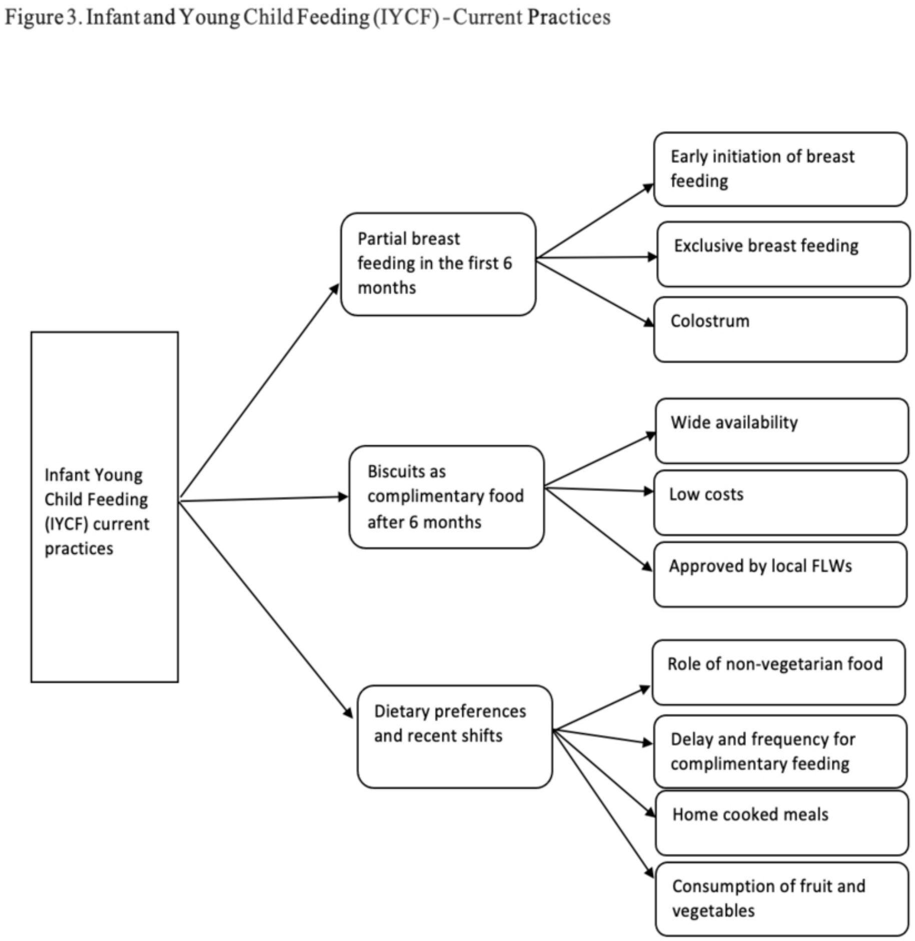

Infant and Young Child Feeding (IYCF) – Current Practices

193x193mm (118 x 118 DPI)

Figure 4. Infant and Young Child Feeding (IYCF) -Key Drivers and Challenges

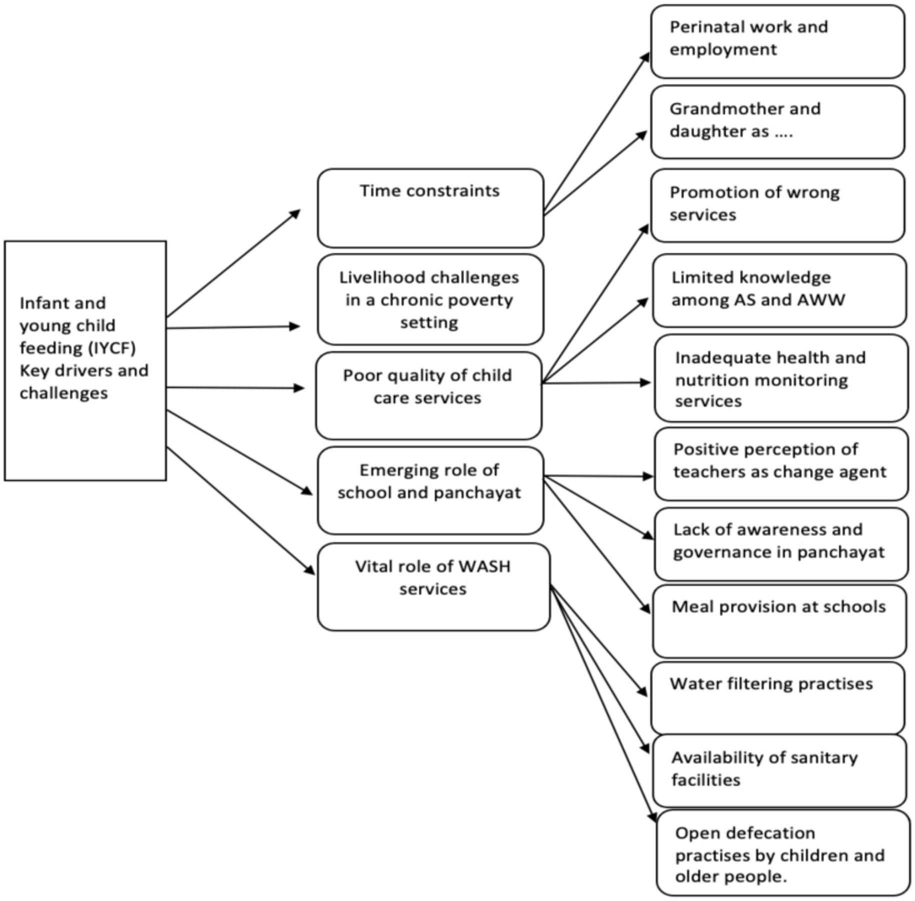

Infant and Young Child Feeding (IYCF) – Key Drivers and Challenges: Connecting subthemes

193x193mm (118 x 118 DPI)

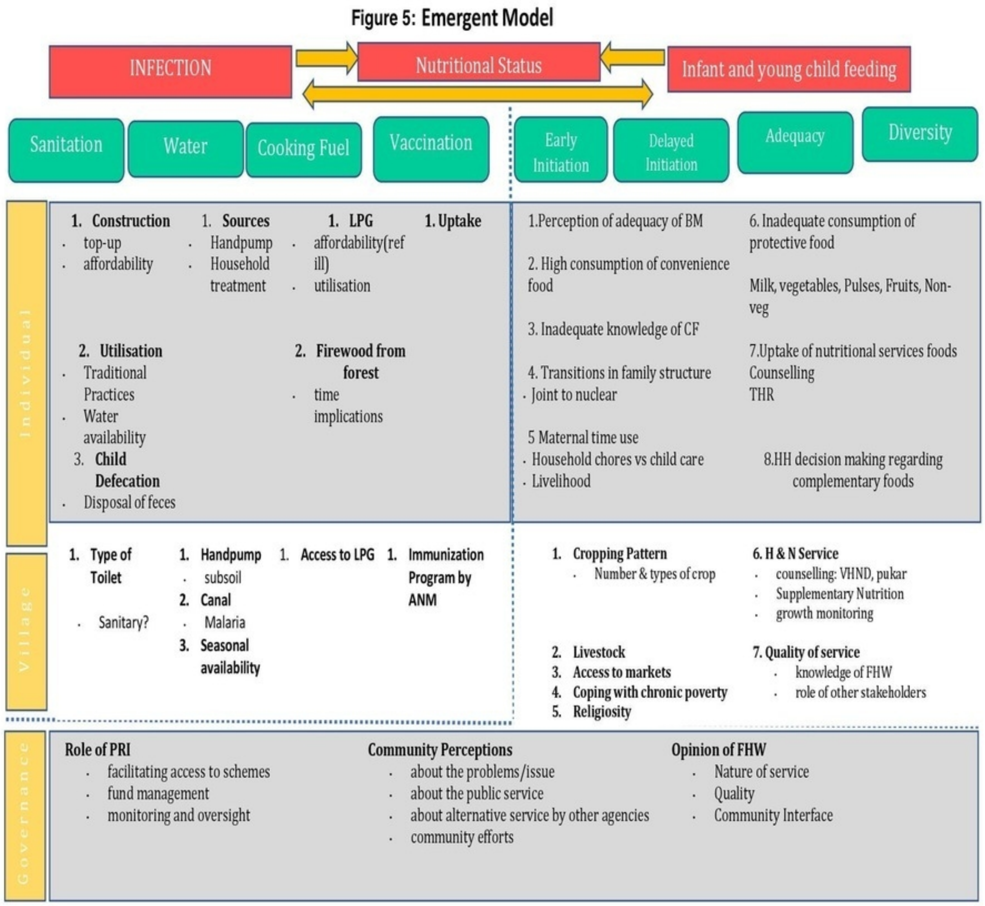

Emergent Model

76x76mm (300 x 300 DPI)

Supplementary Table

Trends in IYCF indicators in India (in %)

	States	Children receiving solid or semi-solid food and breast milk (%)		Trends in IYCF (%)
		NFHS 3 (2005 – 2006)	NFHS 4 (2015 – 2016)	
**India**		52.6	42.7	-9.9
**North**	Rajasthan	38.7	30.1	-8.6
	Punjab	50.9	41.1	-9.8
	Uttar Pradesh	41.2	32.6	-8.6
**North East**	Sikkim	85.4	61.8	-23.6
	Manipur	77.4	78.38	1.4
	Arunachal Pradesh	80.2	53.6	-26.6
**West**	Gujarat	54.1	49.4	-4.7
	Maharashtra	45.5	43.3	-2.2
**Central**	Madhya Pradesh	46	38.1	-7.9
	Chhattisgarh	49	53.8	4.8
**East**	Bihar	54.5	30.7	-23.8
	Jharkhand	60.2	47.2	-13
	West Bengal	47.1	52	4.9
	Odisha	65.4	54.9	-10.5
**South**	Karnataka	69.7	46	-23.7
	Tamil Nadu	81.2	67.5	-13.7
	Kerala	93.9	63.1	-30.8

Source: Dasgupta, Chaand and Rakshit (2018) [19]

For peer review only

Core Theme	Sub-theme	Participant type	FDG/KII	Example Quote
**Infant and Young Child Feeding (IYCF) – Current Practices**	*Partial breastfeeding in the first 6 months*	Mothers, Kushalgarh	FDG	"*When the child starts crying after birth then breast milk is produced and the mother is ready to breastfeed the child.*"
		Mothers, Ghatol	FDG	"*No, we do not breastfeed the child soon after childbirth. Breastfeeding depends on the mother's ability to produce milk. Breastfeeding begins only after midnight if a birth occurs during the evening.*
		AS, Kushalgarh	KII	"*If the baby is delivered at home breastfeeding is starts immediately after birth. Most cases I am present during the childbirth and hence I ensure that breastfeeding starts within an hour for cases that I attend.*"
		Grandmothers, Kushalgarh	FGD	"*Only when the mother's milk secretion does not start, goat's milk is given. Honey or any water is not given.*"
		Mothers, Ghatol	FGD	"*Mothers of children who are on breast milk work inside the village, so they can be summoned home whenever the child cries"*
		AWW, Ghatol	KII	"*In the summer season the child becomes thirsty, so water is given to the child. We also recommend giving water sometimes.*"
		Grandmothers, Kushalgarh	FDG	"*When the child's mother is out of the village due to some work or is working in the field, goat milk is given when the child cries.*"
	Biscuits as complementary food after 6 months	Grandmothers, Kushalgarh	FGD	"*Children like biscuits and it is easy to feed them. We give them one biscuit and they keep eating it. We do not have to feed them.*"
		Mothers, Ghatol	FGD	"*This is the cheapest option and is easily available in the village.*"
		ANM, Kushalgarh	KII	"*We tell the mothers that they can give biscuits to the child, at least it will keep them full.*"
		Mothers, Ghatol	FDG	"*The baby starts drinking milk (dairy) after 6 months. We also give rab (grounded maize porridge), biscuits dipped in the water and sometime daal ka pani (lentil soup). We know what to give and what not to. The AWW also tells us about this. We only take care of all things related to the child like cooking and feeding.*"

For peer review only

	Dietary preferences and recent shifts	Mothers FGD, Kushalgarh	FGD	*"We feed them separately. We do not feed them with us. Whenever the children get hungry we feed them. They do not understand anything; they eat whatever we give them."*
		Ward Panch, Ghatol	KII	*"Nobody eats non-vegetarian food as they have become 'followers'. This is a recent trend. Hawan (worshipping with holy fire) was conducted in the village after which every family has stopped eating. No question of giving it to children."*
		Teacher, Kushalgarh	KII	*"Very few families eat non-vegetarian (foods). Those who eat also give their children. This is made only if there is a guest at home or there is any occasion. Chicken is mostly eaten (on such occasions). Eggs are not eaten as they are left to hatch and the goats are sold."*
		Teacher, Kushalgarh	KII	*"Fruits are not very common in daily diet. Only when the parents go to the market they get fruits. There is availability of local fruits which are not too good for children, like tamarind and plum."*
		ANM, Ghatol	KII	*"Some vegetables are grown in the backyard of every home, mostly beans and brinjal. This is used quite regularly. Potatoes, tomatoes and onion are cooked almost daily. The local vegetables like spinach are seldom grown as people do not want to eat them."*
	Time Constraints	Grandmother, Kushalgarh	FDG	*"The mother cannot sit at home taking care of the child. Who will look after the animals, get water or cook? They also have to do agricultural work."*
		ANM, Ghatol	KII	*"Some mothers work a lot and hence are not able to take care of the child adequately. Grandmothers help, but they are also old people and cannot do much."*
		Grandmother, Ghatol	FDG	*When my daughter-in-law works outside, then we grandmothers take care of the children. Like when the child is crying and have to be fed; we keep them in our laps and make them comfortable. We keep an eye on the children and their activities."*
		School Teacher, Kushalgarh	KII	*"Most people here are in agriculture. Both males and females of all families take part in agriculture. Men play an important role of sowing and harvesting while women do the rest. Women are engaged all year round."*
		Ward Panch, Ghatol	KII	*"Most houses keep goats, some also have cows. Buffaloes are very less in the village. All of these animals do not give milk. Milk from goat is used for consumption at home. Cow*

**Infant and Young Child Feeding (IYCF) – Key Drivers and Challenges**	*Livelihood challenges in a chronic poverty setting*			*and buffalo milk is used for making ghee. Few families also sell it. They do not feed milk to their children"*
		Mothers, Ghatol	FDG	*Taking care of the animals is the work of women as they stay at home. They are the ones who clean the shed and give the animals fodder and water."*
		School Teacher, Ghatol	KII	*"Some men from the village work in Mayur mills (textile mill near Banswara). Some also work as wage labour, but only during lean seasons. Women with small children do not go out of the village to work."*
	Poor Quality of Childcare services	AWW, Kushalgarh	KII	*"Neither LS (Lady Supervisor) or the CDPO (Child Development Project Officer) visits our centre. We only go to the sector meetings where they ask us about the number of pregnant women registered, children born and packets of THR distributed. They sometime give us new information. The focus is on children coming to AWC."*
		AWW, Ghatol	KII	*"We take the weight of the child who comes for the vaccination. Sometimes the mothers do not get the card when they come for vaccination, so weight cannot be recorded in the (Mamta) card."*
		AWW, Kushalgarh	KII	*"We take weight, but currently the weighing machine is not working."*
	Emerging Role of Schools and Panchayats	School Teacher, Kushalgarh	KII	*"The AWC is in the school premises; we can always keep a check whether children are getting their meals. The AWW and the other staff also come in time and regularly open the centre."*
		School Teacher, Ghatol	KII	*"We are already overburdened with the work of the school. It is not possible to check the functioning of the AWC. However sometimes while going back from school I go to the AWC and speak to the workers. I have seen their records and gave them suggestions to maintain it better. What more can we do?"*
		AWW, Ghatol	KII	*"Toilets have been constructed in every household but not everyone is using it. Elder members prefer open defecation."*
		ANM, Kushalgarh	KII	*"Few families have constructed the toilets and very few members use it due to scarcity of water."*

For peer review only

	Vital role of WASH practices	Ward Panch, Kushalgarh	KII	*"Those who constructed the toilets have not got their due payments. I have been following up with the Sarpanch's office regularly, but there is no response. This discourages those who have not yet constructed."*
		Ward Panch, Ghatol	KII	*"The government has constructed toilets in every household. Whatever facilities the government has provided us, I have provided in this village. Half of the villagers use toilets. There is a delay in payment, but the Sarpanch has told me that it will be soon released."*

For peer review only

COREQ (COnsolidated criteria for REporting Qualitative research) Checklist

A checklist of items that should be included in reports of qualitative research. You must report the page number in your manuscript where you consider each of the items listed in this checklist. If you have not included this information, either revise your manuscript accordingly before submitting or note N/A.

Topic	Item No.	Guide Questions/Description	Reported on Page No.
**Domain 1: Research team and reflexivity**			
Personal characteristics			
Interviewer/facilitator	1	Which author/s conducted the interview or focus group?	
Credentials	2	What were the researcher's credentials? E.g. PhD, MD	
Occupation	3	What was their occupation at the time of the study?	
Gender	4	Was the researcher male or female?	
Experience and training	5	What experience or training did the researcher have?	
Relationship with participants			
Relationship established	6	Was a relationship established prior to study commencement?	
Participant knowledge of the interviewer	7	What did the participants know about the researcher? e.g. personal goals, reasons for doing the research	
Interviewer characteristics	8	What characteristics were reported about the inter viewer/facilitator? e.g. Bias, assumptions, reasons and interests in the research topic	
**Domain 2: Study design**			
Theoretical framework			
Methodological orientation and Theory	9	What methodological orientation was stated to underpin the study? e.g. grounded theory, discourse analysis, ethnography, phenomenology, content analysis	
Participant selection			
Sampling	10	How were participants selected? e.g. purposive, convenience, consecutive, snowball	
Method of approach	11	How were participants approached? e.g. face-to-face, telephone, mail, email	
Sample size	12	How many participants were in the study?	
Non-participation	13	How many people refused to participate or dropped out? Reasons?	
Setting			
Setting of data collection	14	Where was the data collected? e.g. home, clinic, workplace	
Presence of non-participants	15	Was anyone else present besides the participants and researchers?	
Description of sample	16	What are the important characteristics of the sample? e.g. demographic data, date	
Data collection			
Interview guide	17	Were questions, prompts, guides provided by the authors? Was it pilot tested?	
Repeat interviews	18	Were repeat inter views carried out? If yes, how many?	
Audio/visual recording	19	Did the research use audio or visual recording to collect the data?	
Field notes	20	Were field notes made during and/or after the inter view or focus group?	
Duration	21	What was the duration of the inter views or focus group?	
Data saturation	22	Was data saturation discussed?	
Transcripts returned	23	Were transcripts returned to participants for comment and/or	

For peer review only

Topic	Item No.	Guide Questions/Description	Reported on Page No.
		correction?	
**Domain 3: analysis and findings**			
Data analysis			
Number of data coders	24	How many data coders coded the data?	
Description of the coding tree	25	Did authors provide a description of the coding tree?	
Derivation of themes	26	Were themes identified in advance or derived from the data?	
Software	27	What software, if applicable, was used to manage the data?	
Participant checking	28	Did participants provide feedback on the findings?	
Reporting			
Quotations presented	29	Were participant quotations presented to illustrate the themes/findings? Was each quotation identified? e.g. participant number	
Data and findings consistent	30	Was there consistency between the data presented and the findings?	
Clarity of major themes	31	Were major themes clearly presented in the findings?	
Clarity of minor themes	32	Is there a description of diverse cases or discussion of minor themes?	

Developed from: Tong A, Sainsbury P, Craig J. Consolidated criteria for reporting qualitative research (COREQ): a 32-item checklist for interviews and focus groups. *International Journal for Quality in Health Care*. 2007. Volume 19, Number 6: pp. 349 – 357

Once you have completed this checklist, please save a copy and upload it as part of your submission. DO NOT include this checklist as part of the main manuscript document. It must be uploaded as a separate file.

For peer review only

BMJ Open

Why India is struggling to feed their young children? A Qualitative analysis for tribal communities

Journal:	*BMJ Open*
Manuscript ID	bmjopen-2021-051558.R2
Article Type:	Original research
Date Submitted by the Author:	14-Jun-2022
Complete List of Authors:	Lakhanpaul, Monica; University College London, Population, Policy and Practice, UCL Great Ormond Street Institute of Child Health; Whittington Health NHS Trust<br>Roy, Susrita; Save The Children<br>Benton, Lorna; University College London, Population, Policy and Practice, UCL Great Ormond Street Institute of Child Health<br>Lall, Marie; University College London, Institute of Education<br>Khanna, Rajesh; Save The Children<br>Vijay, Virendra ; Indian Institute of Technology Delhi<br>Sharma, Sanjay ; University College London, Engineering for International Development Centre<br>Manikam, Logan; University College London Institute of Epidemiology and Health Care, Department of Epidemiology and Public Health; Aceso Global Health Consultants<br>Santwani, Neha; Save the Children Fund, Health and Nutrition<br>Reddy, Hanimi; Save the Children, National Support Office<br>Chaturvedi, Hemant; Save the Children<br>Allaham, Shereen ; University College London Institute of Epidemiology and Health Care, Department of Epidemiology and Public Health; Aceso Global Health Consultants Ltd,<br>Pattanaik, Satya Prakash ; Save the Children<br>Singh, Tol; Save the Children<br>Pandya, Pramod; Save the Children<br>Dang, Priyanka; Save the Children<br>Parikh, Priti; University Colege London, Engineering for International Development Centre, Faculty of the Built Environment, London, UK
**Primary Subject Heading**:	Global health
Secondary Subject Heading:	Evidence based practice, General practice / Family practice, Nutrition and metabolism, Paediatrics, Qualitative research
Keywords:	NUTRITION & DIETETICS, Community child health < PAEDIATRICS, PUBLIC HEALTH

SCHOLARONE™
Manuscripts

I, the Submitting Author has the right to grant and does grant on behalf of all authors of the Work (as defined in the below author licence), an exclusive licence and/or a non-exclusive licence for contributions from authors who are: i) UK Crown employees; ii) where BMJ has agreed a CC-BY licence shall apply, and/or iii) in accordance with the terms applicable for US Federal Government officers or employees acting as part of their official duties; on a worldwide, perpetual, irrevocable, royalty-free basis to BMJ Publishing Group Ltd ("BMJ") its licensees and where the relevant Journal is co-owned by BMJ to the co-owners of the Journal, to publish the Work in this journal and any other BMJ products and to exploit all rights, as set out in our licence.

The Submitting Author accepts and understands that any supply made under these terms is made by BMJ to the Submitting Author unless you are acting as an employee on behalf of your employer or a postgraduate student of an affiliated institution which is paying any applicable article publishing charge ("APC") for Open Access articles. Where the Submitting Author wishes to make the Work available on an Open Access basis (and intends to pay the relevant APC), the terms of reuse of such Open Access shall be governed by a Creative Commons licence – details of these licences and which Creative Commons licence will apply to this Work are set out in our licence referred to above.

Other than as permitted in any relevant BMJ Author's Self Archiving Policies, I confirm this Work has not been accepted for publication elsewhere, is not being considered for publication elsewhere and does not duplicate material already published. I confirm all authors consent to publication of this Work and authorise the granting of this licence.

For peer review only

Why India is struggling to feed their young children? A Qualitative analysis for tribal communities

Professor Monica Lakhanpaul; MBBS, DM, MPH, FRCPCH [1,2]
Susrita Roy; MA, MPhil[3]
Dr Lorna Benton; PhD [1]
Professor Marie-Carine Lall; MPhil, PhD [4]
Dr. Rajesh Khanna; MBBS, DNB, MSc [3]
Professor Virendra Kumar Vijay, PhD [5]
Sanjay Sharma; MSc, LLB [6]
Dr Logan Manikam, MBChB, FFPH, PhD [7,8]
Neha Santwani; MSc [3]
Dr Hanimi Reddy; MPhil, MSc, PhD [3]
Hemant Chaturvedi; MSc [9]
Shereen Allaham; PharmD, MPH [7,8]
Satya Prakash Pattanaik [6]
Tol Singh [9]
Pramod Pandya [9]
Priyanka Dang; MBA [3]
Dr Priti Parikh; MPhil, PhD [10]

Institutions

1. Population, Policy and Practice, UCL Great Ormond Street Institute of Child Health, University College London, London, WC1N 1EH, UK
2. Whittington Health NHS Trust, London, N19 5NF, UK
3. Save the Children, National Support Office, Gurugram, India
4. Institute of Education, University College London, UK
5. Indian Institute of Technology Delhi (IIT), India
6. Save the Children India, Rajasthan State Programme Office, Jaipur, India
7. Department of Epidemiology and Public Health, University College London Institute of Epidemiology and Health Care, London, UK.
8. Aceso Global Health Consultants Limited, London, UK.
9. Save the Children, Rajasthan, India
10. UCL Engineering for International Development Centre, Bartlett School of Construction and Project Management, The Bartlett, UCL Faculty of the Built Environment, London, UK

For peer review only - http://bmjopen.bmj.com/site/about/guidelines.xhtml

Corresponding author:
Monica Lakhanpaul,
Professor of Integrated Community Child Health
Population, Policy and Practice, UCL Great Ormond Street Institute of Child Health, 30 Guilford Street, University College London, London WC1N 1EH.
Email address: m.lakhanpaul@ucl.ac.uk

Word count: 8075

Acknowledgements:

PANChSHEEEL gratefully acknowledges the input from our community champions and field team, which made this project possible. We would like to thank MRC for funding the PANChSHEEEL project. We would also like to thank Isabel-Catherine Demel, a medical student who helped out in formatting the paper and Shyantani Saha, a research assistant at University College London who helped in incorporating the reviews and developing the manuscript. The opinions in this paper solely reflect the views of the authors and not of organizations who have financially supported this research. All data used in this research is the property of the authors. The data that support the findings of this study are available from the corresponding author, Prof Monica Lakhanpaul, upon reasonable request. Prof Monica Lakhanpaul was supported by the National Institute for Health Research (NIHR) Collaboration for Leadership in Applied Health Research and Care (CLAHRC) North Thames at Bart's Health NHS Trust. This work is supported by the NIHR GOSH BRC. Dr Logan Manikam and Shereen Al Laham are funded via an NIHR Advanced Fellowship (Ref: NIHR300020).

For peer review only - http://bmjopen.bmj.com/site/about/guidelines.xhtml

Why India is struggling to feed their young children? A Qualitative analysis for tribal communities

Abstract

Objective: This interdisciplinary qualitative study aims to explore the health, education, engineering and environment (HEEE) factors impacting on feeding practices in rural India. The ultimate goal of the PANChSHEEEL project is to identify challenges and opportunities for improvement to subsequently develop socio-culturally appropriate, tailored, innovative interventions for the successful implementation of appropriate IYCF (Infant and Young Child Feeding) practices locally.

Design: Qualitative research method, involving 5 phases, (1) identification of local feeding practices; (2) identification of the local needs and opportunities for children aged 6-24 months; (3)-(5) analysis of the gathered qualitative data, intervention design, review and distribution.

Setting: Nine villages in two community development blocks, i.e. Ghatol and Kushalgarh, located in the Banswara district in Rajasthan, India.

Participants: 68 participants completed semi-structured interviews or focus group discussions including: mothers, grandmothers, Auxiliary Nurse Midwife, Anganwadi Worker, ASHA Sahyogini, school teachers, and local elected representative.

Phenomenon of Interest: IYCF practices and the factors associated with it.

Analysis: Thematic analysis.

Results: Our results could be broadly categorized into two domains: (1) The current practices of IYCF and (2) The key drivers and challenges of IYCF. We explicate the complex phenomena and emergent model focusing upon: mother's role and autonomy, knowledge and attitude towards feeding of young children, availability of services and resources that shape these practices set against the context of agriculture and livelihood patterns and its contribution to availability of food as well as on migration cycles thereby affecting the lives of 'left behind' and access to basic health, education and infrastructure services.

Conclusions: This interdisciplinary and participatory study explored determinants impacting feeding practices across political, village and household environments. These results shaped the process for co-creation of our context-specific intervention package.

Word count: 281

For peer review only - http://bmjopen.bmj.com/site/about/guidelines.xhtml

Keywords: Feeding practices; Children; Infant; Early Interventions**;** Participatory; Community.

Article Summary

Strengths and Limitations of this study

Strengths

- As an interdisciplinary study, the PANChSHEEEL (Participatory Approach for Nutrition in Children: Strengthening Health Education Engineering and Environment Linkages - HEEE) project explored a plethora of themes across the four domains of health, education, engineering and environment (HEEE) to evaluate contributing factors, current challenges and potential opportunities in Infant and Young Child Feeding (IYCF) practices in India
- The ultimate objective being to co-develop a context specific multisectoral HEEE Nutrition Intervention Package that is socio-culturally appropriate, innovative and targeted to enhance IYCF practices of 6-24 months aged children locally
- This study employed a bottom-up approach, involving a close collaboration with the local affected population, interdisciplinary teams of researchers, non-government organizations (NGOs), as well as pre-existing and emergent research evidence not only in identifying current feeding practices, and perceived barriers but also in future opportunities for intervention.

Limitations

- Notwithstanding the scope of further research to be conducted in neighboring areas in rural India to ensure the validity and generalizability of the findings, this study was conducted in a limited geographic scale with data gathered across two community blocks in Rajasthan.

For peer review only - http://bmjopen.bmj.com/site/about/guidelines.xhtml

Introduction

The Global Nutrition Report (2018) reported that globally 150.8 million and 50.5 million children under the age of five years are stunted (inadequate height for age) and wasted (inadequate weight for height) respectively [1]. With 38.4% stunted children, India contributed a third of the world's burden for stunting in children under 5 years. With respect to wasting, India accounts for an even larger proportion, with 25.5 million children affected nationally – a number equivalent to half of the global burden of wasting [2]. Conscious of the fact that adequate nutrition enhances cumulative lifelong learning capacity and adult productivity, the Government of India (GOI) recognizes that improving nutrition will be of vital importance in poverty alleviation and the country's economic development in the long-run [3].

A variety of conceptual models have been used to explain the myriad and complex set of undernutrition determinants. One such model, proposed by the United Nations Children's Fund [4], illustrates the socio-ecological determinants influencing undernutrition. According to this model, inadequate dietary intake and infectious diseases are two key proximate factors for undernutrition alongside household food insecurity, inadequate care and feeding practices and unhealthy household environments.

Infant Young Child Feeding (IYCF) practices entail initiation of breastfeeding in the first hour of birth, exclusive breastfeeding till 6 months followed by complementary feeding after sixth months as well as optimal feeding practices essential for children until the age of two. Complementary feeding includes the introduction of age-appropriate semi-solid food alongside breast milk after six months of exclusive breast feeding [5]. Universal coverage of optimal breastfeeding can prevent 13% of global deaths in children less than five years of age, while appropriate complementary feeding practices could result in an additional 6% reduction in under-five mortality [6]. With 80-85% of brain growth taking place during the first two years of life, optimal IYCF plays an essential role also with respect to cognitive development, as reiterated by The Global Nutrition Report [7].

When comparing India's IYCF indicators with the global and regional indicators, two important phenomena become apparent: (i) India's breastfeeding indicators (early initiation 41.5%, exclusive breastfeeding 54.9%) are at par with the global and South Asian averages, however they are lower than that of its immediate neighbours, Nepal (early initiation 54.9%, exclusive breastfeeding 65.2%) and Bangladesh (early initiation 50.8%, exclusive breastfeeding 55.3%), even though these countries have lower HDI score compared to India; and, (ii) India's complementary feeding indicators are lagging not only the global and South Asian averages, but also related to its neighbouring countries. While Minimum Dietary Diversity (MDD) in India (19.9%) is similar to the South Asian average, it is lower than that of the global average

For peer review only - http://bmjopen.bmj.com/site/about/guidelines.xhtml

(29%) and its neighbours Nepal (45%) and Bangladesh (26.6%). The major challenge is seen for Minimum Acceptable Diet (MAD) as the rate in India (MAD 9.6%) is lower not only than that of the global (18%) and regional average (12%), but also it is much lower than that of its neighbors Nepal (35.8%) and Bangladesh (22.8%). [8]

Complementary feeding rates declined in India between the two National Family Health Surveys of 2005-06 and 2015-16 from 52.6% to 42.7%. The contents of NFHS-5 (2019-2021) are similar to NHFS-4 and allow for comparison over time. [9] The decline was seen in nearly all the regions and states but there was marked intra-country variation in the degree of decline. The highest decline was observed in the southern states (14% to 31%) which have comparatively better performing health systems in the country, while the northern states with weaker health systems had a lower degree of decline (8% to 10%). This could be attributed to the fact that the northern states already had much lower level of complementary feeding rate (during NFHS-3) compared to the southern states, so that any further decline becomes even more concerning. In the northern region, Rajasthan showed a decline of 9% in the complementary feeding rate. (supplementary file 1).

Disaggregated data from the NFHS 5[] and NHFS 4 [10] points to Rajasthan state in the northern region recording one of the lowest IYCF indicators. While an average of 35 % children under age 3 years were breastfed within one hour of birth and 64% children under age 6 months exclusively breastfed, only 34% of children aged 6-8 months received complementary feeding along with breastmilk. About one-third (34%) of children age 6-23 months were fed the recommended minimum number of times per day, 9.7% had MDD and only 3.4% had access to MAD [9] [10] The MAD score in Rajasthan is the lowest score among the larger states in India [10]. Within the state, the district Banswara which is a predominantly tribal area, has IYCF indicators worse than the state average and many of the other 32 districts[11]. Table 1 shows the IYCF indicators in India, Rajasthan and Banswara districts.

Table 1: IYCF Indicators in India, Rajasthan and Banswara district, NFHS-4; 2015-16 (in %) & NFHS-5(2019-2021)

For peer review only - http://bmjopen.bmj.com/site/about/guidelines.xhtml

	NFHS-4			NFHS-5		
**Indicators**	**India**	**Rajasthan**	**Banswara**	**India**	**Rajasthan**	**Banswara**
Early initiation of breastfeeding	41.5	28.4	37.8	41.8	40.7	33.5
Exclusive breastfeeding under 6 months	54.9	58.2	57.1	63.7	70.4	66.2
Introduction of solid, semi-solid or soft foods (6-8) months	42.7	30.1	Not available	45.9	38.0	Not available
Complementary feeding – Minimum Acceptable Diet	9.6	3.4	0.8	11.3	8.4	9.7

Several Indian studies have sought to unravel some key aspects of India's malnutrition scenario, addressing important determinants at three levels: (i) household (maternal time constraint, dwindling family size, mother's age and education; lack of adequate knowledge; poor uptake of existing nutritional services; child targeted market with wide availability and consumption of ready-to-eat marketed food items); (ii) community (social and economic context; feminization of agriculture; fragile food security/seasonal food paucity due to less focus on food crops and vegetables; dwindling livestock – especially milk producing animals; low connectivity to remote locations; migration; exposure to media); and, (iii) government (inadequate and unresponsive Integrated Child Development Scheme [ICDS] and health care system; paucity of technical knowledge among service providers regarding IYCF). Several studies over the last decade have examined varying associations between household environmental characteristics and stunting in under-five children, highlighting the need for interdisciplinary research [9]][11-16].

Our study, the Participatory Approach for Nutrition in Children: Strengthening Health, Education, Environment and Engineering Linkage (PANChSHEEEL) funded by the Medical Research Council (UK) was designed to: (i) explore health, education, engineering and environment (HEEE) factors that influenced Infant and Young Child Feeding (IYCF) practices and (ii) develop a socio-culturally appropriate, tailored, innovative and integrated cross sector HEEE package to support optimal IYCF practices. This is central to the convergent action planning process of India's National Nutrition Mission or the POSHAN Abhiyaan (Prime Minister's Overarching Scheme for Holistic Nutrition) that has IYCF as the first target to be monitored.

For peer review only - http://bmjopen.bmj.com/site/about/guidelines.xhtml

Conceptual framework

Drawing upon the review of the above studies, the principal investigator and the co-investigators synthesized the determinants from the literature review to frame the HEEE conceptual framework as a socio-ecological model (Figure 1). This framework sought to synthesize the complex interplay of factors across three environments: political, village and household; and the inter-linkages between institutions, initiatives (schools, health services, ICDS and public distribution system) and communities. The qualitative studies that were reviewed focused on undernutrition or stunting and not specifically on IYCF and child feeding practices that have assumed crisis proportions but have received scanty attention among public health nutrition researchers in India.

Figure 1: HEEE Conceptual Framework [17][18]

The formative phase of the PANChSHEEEL study was thus framed to develop an understanding of a deep-dive interplay of the determinants of IYCF to inform the co-designing of an intervention model that can address this crisis in such contexts.

This paper presents the qualitative findings of the PANChSHEEEL study; that aims to identify and document local community IYCF practices including water, sanitation & hygiene (WASH) and energy practices, to identify local challenges, drivers, resources, opportunities and needs for IYCF in 6-24 month old children at individual, household, community and environmental level; and, to map the linkages between identified opportunities and challenges in order to determine how the needs identified can be addressed.

Methodology

The larger PANCHShEEEL study used both qualitative and quantitative research methods, triangulating the two to synthesize evidence. This paper presents the qualitative component of the formative study, in line with COREQ guidelines.

Study setting

For peer review only - http://bmjopen.bmj.com/site/about/guidelines.xhtml

The study was conducted in nine villages in two community development blocks (herein after, blocks) of Banswara district in Rajasthan, India. These two blocks, Ghatol and Kushalgarh, were purposively chosen to represent district diversity. The Ghatol Block is located in the 'command area' of the Mahi River Dam; all villages are irrigated by canals and have multiple crops yearly. In contrast, Kushalgarh is dry and semi-arid with poor irrigation; consequently, most villages are mono-crop areas. The village selection process is represented in the following consort diagram (Figure 2).

Figure 2: Consort Diagram for selection of Study Villages [18]

Patients and Public involvement and Engagement

In line with the PANChSHEEL participatory approach, considerable efforts were placed on Patient and Public Involvement and Engagement (PPIE). Suitable individuals and representatives from both the Ghatol and Kushalgarh Block were identified and subsequently engaged in all steps of the study, i.e. protocol development, study design, results dissemination.

The Core Research Team was further supported by Community Researches from the two study blocks who aided in steering the project phases locally. With the input from 'Save the Children' Rajasthan, two well-suited individuals were identified who had been proactive collaborators in previous projects by 'Save the Children' India. They were mainly involved in selecting suitable candidates who could engage actively in project activities, as well as coordinating with the Community Champions (CCs), who were crucial in establishing networks with potential study participants among Community Members (CMs).

Relevant stakeholders were identified and selected in a stakeholder mapping workshop as part of the formative study phase. Through snowballing, i.e. a recruitment method where existing participants are involved in the identification and recruitment of future participants using their social networks, the research team remained open to potential new stakeholder(s) identified during the data collection process. The recruitment of study participants was based on willingness to participate and availability.

Respondent selection

The respondents included stakeholders who play a critical role in feeding and caring for young children. There are three cadres of frontline workers at the village level in Rajasthan: (i) the Auxiliary Nurse Midwife (ANM), the multi-purpose female health worker; (ii) the Anganwadi Worker (AWW), the cadre of the Integrated Child Health Services (ICDS) who runs the Anganwadi Centre (AWC) at each village; and (iii) the ASHA Sahyogini (AS), the community health worker (ASHA in other states). One ANM was selected

For peer review only - http://bmjopen.bmj.com/site/about/guidelines.xhtml

from each of the sub-centers in the study villages; in case of sub-centers with 2 ANMs, the senior ANM was selected. Owing to the size of the village, generally one AWW or AS was chosen but in the case where the village had more than one AWW or AS, then both were interviewed as they represented different geographic and social strata. One school from each village was chosen and the principal/head or the most senior teacher was interviewed. The ward or local Sarpanch of each village was also interviewed. At household level, mothers and grandmothers who were literate and conversational (with at least one 6–24-month-old child) were identified and selected based on their availability and willingness to participate either during the household survey or by frontline health workers.

Data collection

Key Informant Interviews (KIIs) and Focus Group Discussions (FGDs) were used for collecting primary data. Semi-structured open-ended interview guides were used to understand respondent knowledge and feeding practices perceptions. FGDs were conducted with mothers and grandmothers. The date, time and meeting place were decided in consultation with AWW and AS. FGD guides included topics on maternal time use, household care giving roles, breastfeeding and complementary feeding, hygiene, play and communication. An approximate of 5-9 questions were asked under each topic and the data collected was classified into 4 major themes (Health and Nutrition, Education, WASH and Cooking Fuels). Participants were encouraged to share their frank opinions, irrespective of the views of others in the group, in order to elicit insights about IYCF issues (Table 2).

FGDs were conducted by community researchers from the same blocks or adjoining block who were able to communicate fluently in the local dialect, *Wagdi.* They were trained on FGD techniques by the principal investigator and co-investigators which included principles and methods of qualitative research with a special focus on interview and group discussions, and an outline of data analysis. Hands-on support was provided by other team members who were well versed with the research methods and local contexts. The duration of the interviews ranged between 25-40 minutes and the FGDs ranged between 45-75 minutes.
We relied on a hybrid data saturation approach: achieving both: (i) a priori thematic saturation (the degree to which identified codes or themes are exemplified in the data) that informed sampling strategy and (i) inductive thematic saturation (the emergence of new codes or themes) that informed data analysis [19].
Field notes based on observations played a crucial role in data collection and analysis. Digital recorders were used to record all KIIs and FGDs. Written consent was taken from key informants whilst for FGDs verbal consent was sought and formally recorded. Audio data was then transcribed (from local language to Hindi) and translated (from Hindi to English) with back translations done for quality assurance. Both audio

For peer review only - http://bmjopen.bmj.com/site/about/guidelines.xhtml

data and text were anonymised to maintain confidentiality and securely stored. A pilot round was conducted in January 2018 following which formal data collection continued for four months. Caution was maintained regarding selection bias of FGD participants due to the active role of frontline health workers in service delivery. FGDs were organized at Integrated Child Development Services (ICDS) or Anganwadi Centers (AWC) centers at each village with care taken to ensure that residents residing far from AWCs were not excluded. This study was conducted according to the guidelines laid down in the Declaration of Helsinki and all procedures involving research study participants were approved by the UCL ethics [Ethics ID 4032/002] and Sigma IRB [10025/IRB/D/21-22]].

Topic Guide

Through the means of a literature review, expert advice and pilot testing with different population subsets, a thematic guide was designed prior to the qualitative data collection process. This guide facilitated the conductance of the KIIs and FDGs, allowing the topic of IYCF to be explored across the four major dimensions (1) Health and Nutrition, (2) Education, (3) Water, Sanitation and Hygiene and (4) Energy to capture current practices, barriers and possible opportunities to foster positive change across all four areas.

Data processing and analysis

The research team undertook daily meetings to reflect upon their key observations and data collected from field notes. Data was transcribed and translated to bring out common major themes based on commonality and differences. Data generated from all sources were subjected to triangulation of mixed qualitative and quantitative methodology [20] and linked thematically for key findings. The qualitative data analysis software, IQDAS was used for the data analysis process. The data was coded and analyzed jointly by the manuscript authors (co-investigators) to ensure consistency and reliability in interpretation. Codes were analyzed across axial and selective codes across respondent categories and sites (villages and blocks). Triangulation was performed across methods and respondents. Results from KIIs and FGDs were compared and assessed for similarities and differences across respondent categories. Findings from villages within a block conveyed similar interpretations and hence reinforced data validity. The investigator group reached a consensus to merge village and block level findings, except in those themes or sub-themes where they presented novel findings. Data analysis was first completed independently by interviewers followed by a series of joint sessions between interviewers and co-investigators.

For peer review only - http://bmjopen.bmj.com/site/about/guidelines.xhtml

For peer review only

Respondent validation

A series of meetings were conducted with representatives from all respondent categories from the same villages to validate the data. Within the qualitative data sets, all steps of a thematic analysis, i.e. (1) identifying, (2) analyzing and (3) interpreting patterns of meaning, i.e. themes, were employed. Respondent validation was facilitated by Community Researchers in September 2018 and closely overseen by local Save the Children staff. This enabled us to refine the emergent themes and model to its final shape.

Quality assurance

The inter-disciplinary team comprised of experts in public health nutrition, epidemiology, social sciences, paediatrics, education and civil and environmental engineering. A dedicated team of co-investigators supervised training, data collection, transcription and translation, data management and analysis. Field teams were oriented with the study protocol, tools and research techniques, including hands-on exercises, before actual data collection began.

Results

A total of 68 participants were recruited (table 2). The information was obtained from FGDs with 17 mothers and paternal grandmothers and 51 key respondents across 9 villages. More than half of the mothers were aged between 18 to 26 years and majority of them (approximately 92%) had either one or two children under 5 years in their household. 61% of the women including both mothers and grandmothers were illiterate. Of the rest who attended formal schooling, there was roughly a 10% dropout rate because they got married early or participation in household work. More than 90% of the women interviewed were involved in the agriculture industry either in the form of farming or livestock rearing. Based on the present study that defines time spent in tending to farming and livestock as 'market work', women spent a greater amount of time in activities of farming and tending livestock.

Table 2: Respondents Categories and Methods

Category of Respondent	Method Used	Numbers
Mothers and Grandmothers	FGD	17
ANM	KII	7

For peer review only - http://bmjopen.bmj.com/site/about/guidelines.xhtml

AWW	KII	13
ASHA Sahyogini	KII	13
School Teacher	KII	9
Local elected representative	KII	9

Deriving from the study objectives, this section is divided into two parts. The first section provides an in-depth description of IYCF practices. The second section will explain the factors (drivers and challenges) associated with it. Online supplementary file 2 illustrates the themes identified and example quotes from focus groups and interviews with respondents. Figure 3 illustrates the current practices of IYCF.

IYCF practices

Figure 3. Infant and Young Child Feeding (IYCF) – Current Practices

✶ *Core theme 1: Partial breastfeeding is a common practice in first six months.*

Most mothers and grandmothers reported that 90% of the births take place in hospitals due to the presence of trained staff who are equipped to handle medical emergencies and breastfeeding was initiated within the first two to three hours of childbirth in hospital. Of the few women who had given birth at home, they also confirmed that breastfeeding starts as soon after birth as possible. Delay in breastfeeding was reported mainly due to "*secretion of milk not starting*." [Grandmothers FGD, Kushalgarh].

"If the baby is delivered at home breastfeeding is starts immediately after birth."
[AS, Kushalgarh]

Giving colostrum was considered mandatory among a majority of respondents, with only a minority of grandmothers in Kushalgarh expressing negative views about colostrum as "dirty milk". All respondents denied giving any pre-lacteals like honey to the children though these practices were reportedly followed in cases of home births, albeit very few.

Most mothers in Ghatol reported that they exclusively breastfed for four to six months. In Kushalgarh, a majority of respondents breastfed till the third month. Breastfeeding frequency in the first six months was reported to be 'on cue', as and when the child cried.

For peer review only - http://bmjopen.bmj.com/site/about/guidelines.xhtml

"Mothers of children who are on breast milk work inside the village, so they can be summoned home whenever the child cries"
[Mothers, Ghatol]

Mothers who resumed their agricultural work after three months reported coming home from work every two hours for breastfeeding. On further prompting it was revealed that, at times, water was given to children below six months, especially during summer months due to the belief that the child gets thirsty. Dissolving biscuits in milk or water and feeding it to children in case they cried or the mother was not available appeared to occur on occasion, thus compromising exclusive breastfeeding. This practice was also promoted by Frontline Health Workers (FHWs). Animal milk, especially goat milk, was given when the mother was unavailable or when the mother's milk amount was perceived to be inadequate.

"*In the summer season the child becomes thirsty, so water is given to the child. We also recommend giving water sometimes*."
[AWW, Ghatol]

✶ *Core theme 2: Biscuits given as complementary food after six months*

Consumption of biscuits as convenience foods increased after six months in both blocks. Soaked in water or tea, they were used as a semi-solid food for local children. Two reasons were cited for this practice: (i) easy availability and low costs (5 INR or 0.7 USD per packet) and, (ii) children liked the sweet taste; hence making it easy to feed.

"Children like biscuits and it is easy to feed them."
[Grandmothers FGD, Kushalgarh]

"*This is the cheapest option and is easily available in the village.*"
[Mothers FGD, Ghatol]

These were given in the morning as the first meal of the day, and/or between meals when children cried. FHWs commonly approved of this practice.

For peer review only - http://bmjopen.bmj.com/site/about/guidelines.xhtml

"*We tell the mothers that they can give biscuits to the child, at least it will keep them full.*"
[ANM, Kushalgarh]

Biscuits were often employed as a method to *pacify* the child when the mother or other care givers were busy in their daily chores. The number of biscuits given per day varied from five to ten per day.

✶ *Core theme 3: Dietary preferences and recent shifts*

On average, complementary feeding was commenced around 7-8months. The main reason of delayed complementary feeding was the child's "lack of interest" in eating semi-solid foods, marked by prolonged feeding time, crying and also fidgeting while eating. A majority of participants reported to be confident regarding appropriate complementary feeding.

"*The baby starts drinking milk (dairy) after 6 months. We know what to give and what not to.*"
[Mothers FGD, Ghatol]

The foods included pulses (in watery consistency), *khichdi* (savoury rice and pulse gruel), *dalia* (porridge with ground wheat with milk or water, mostly sweet) or small pieces of roti dipped in milk. The use of commercial baby food was nominal. FHWs reported that children were fed four to five times a day on average; mothers and grandmothers, however, reported demand feeding to be common practice, i.e. feeding when the children cried.

"*Whenever the children get hungry we feed them.*"
[Mothers FGD, Kushalgarh]

Most mothers from both blocks reported that, because children ate in small quantities, it was difficult to cook separately for them every day. Neither FHWs nor the mothers had clarity about the frequency and quantity to be fed to children. Portion size varied depending on the child's "interest". All mothers confirmed that they received packets of Take-Home Ration (THR) regularly, but had little knowledge of the proper recipe.

Consumption of non-vegetarian food was dwindling while those who consumed animal products, e.g. meat and eggs, did so discretely. This was attributed to religious reasons, locally termed the '*bhagat'* (disciple) culture.

For peer review only - http://bmjopen.bmj.com/site/about/guidelines.xhtml

"This is a recent trend. Hawan (worshipping with holy fire) was conducted in the village after which every family has stopped eating. No question of giving it to children."
[Ward Panch, Ghatol]

This practice was more prevalent in Ghatol, where more respondents ascribed to these beliefs.
In Kushalgarh, hens were reared in most households, consequently resulting in a higher availability and consumption of non-vegetarian foods. However, due to religious values, respondents often denied its consumption. .

"This is made only if there is a guest at home or there is any occasion. Chicken is mostly eaten (on such occasions)."
[Teacher, Kushalgarh]

Responses from both blocks confirmed that children under five years were rarely given non-vegetarian food, and if so, only in small quantities.
Consumption of fruits, though unanimously considered to be beneficial, was rare on account of availability and cost. Fruits are generally available in the markets located far away from the villages; more so in Kushalgarh. Purchase was possible only when a family member visited these markets.

"*Fruits are not very common in daily diet. Only when the parents go to the market they get fruits."*
[Teacher, Kushalgarh]

Vegetables consumption also comprised "*potatoes, tomatoes and onions almost daily*." [ANM, Ghatol] There was less consumption of green leafy vegetables, for children, even though it was readily available.

Key Drivers and Challenges

IYCF practices described in the previous sections are attributable to contextual issues emerging at the household, community and government level. Differences evident between Ghatol and Kushalgarh include cropping patterns, market access and fruit and vegetable availability. These differences aside, there were similarities in the drivers of IYCF practices between these two blocks. Figure 4 presents the connecting

For peer review only - http://bmjopen.bmj.com/site/about/guidelines.xhtml

subthemes of the key drivers and challenges of IYCF practices.

Figure 4. Infant and Young Child Feeding (IYCF) – Key Drivers and Challenges: Connecting subthemes.

✶ *Core theme 4: Time-constrained mother and role of other family members*

Time constraints amongst mothers emerged as a critical determinant accounting for lack of exclusive breastfeeding (infants below six months) and feeding (home cooked food) at regular intervals (to older children).

"*The mother cannot sit at home taking care of the child. Who will look after the animals, get water or cook? They also have to do agricultural work.*"
[Grandmothers FGD, Kushalgarh]

All mothers, while being primary caregivers, reported that they were guided and influenced by grandmothers, other family members and FHWs. Mothers stayed at or worked in close proximity to their homes until the child was about two months old. During this time, they continued with cooking and other household chores, as well as outdoor work, i.e. firewood and water collection. Mothers from Ghatol reported that they did not go out for work outside the villages. Occasionally, mothers took up daily work in nearby factories, but only once their child was at least one year of age. Women in Kushalgarh migrated to nearby towns and districts as a family, once children were above six months.

"*When my daughter-in-law works outside, then we grandmothers take care of the children.*"
[Grandmothers FGD, Ghatol]

Teachers reported that also elder siblings, especially girls, were often absent from school to take care of younger siblings, especially in families where both parents had migrated (seasonally) or if the primary care giver was unavailable.

✶ *Core theme 5: Livelihood challenges in a chronic poverty setting*

For peer review only - http://bmjopen.bmj.com/site/about/guidelines.xhtml

Agriculture is the main source of livelihood and revenue for families in both blocks, despite clear differences in irrigation facilities and cropping pattern in Ghatol and Kushalgarh.

"*Most people here are in agriculture. Both males and females of all families take part in agriculture.* [School Teacher, Kushalgarh]

Women in all study villages were engaged in agriculture and livestock farming throughout the year; men took part in agriculture only during sowing and harvesting seasons. Some women were engaged under the National Rural Employment Guarantee Scheme though employing mothers with children aged less than two years was not common practice. Local wage labor was reported to be a common source of income for men residing in Ghatol villages due to the proximity to the district town and a cloth mill. Circular migration to urban areas of the adjacent states of Madhya Pradesh and Gujarat was common among men in Kushalgarh.

"*Some men from the village work in Mayur mills (textile mill near Banswara). Some also work as wage labour, but only during lean seasons.*" [School Teacher, Ghatol]

✶ *Core theme 6: Childcare services fails to deliver on most counts*

Proactive steps are being taken by government programs to address IYCF related challenges. Breastfeeding information was mostly provided at childbirth facilities and, these practices were much better compared to complementary feeding. The overwhelming evidence from all the villages was that level of knowledge and counseling skills were poor among AWW and AS. They were not aware of malnutrition indicators and appropriate complementary foods, often promoting the introduction of animal milk, semi-solid or solid food into a baby's diet before the age of six months. Infant weight was recorded in some cases.

"We take weight, but currently the weighing machine is not working." [AWW, Kushalgarh]

In many villages, several (non-health) key informants reported that AWWs mostly catered to community needs staying close to the AWC. The supervisory cadres rarely visited the villages.

"*Neither LS (Lady Supervisor) nor the CDPO (Child Development Project Officer) visits our centre. We only go to the sector meetings where they ask us about the number of pregnant women registered,*

For peer review only - http://bmjopen.bmj.com/site/about/guidelines.xhtml

children born and packets of THR distributed. "

[AWW, Kushalgarh]

Ghatol received more support from the district level than Kushalgarh owing to its proximity to the headquarters; this was further complemented by the presence of NGOs and other civil society actors in Ghatol.

✶ *Core theme 7: Emerging role of schools and panchayats in health and nutrition programs*

School teachers were an important resource for local communities; the schools functioned regularly and efficiently with teachers attending school daily. Mid-day meals were provided. With male members mostly working outside the village (either daily wage or migration), parents' participation in these meetings were occasional. According to school teachers (mostly male), fathers interacted with them with few mothers participating. The state has recently formulated at policy for co-location of AWC at school premises.

"*The AWC is in the school premises; we can always keep a check whether children are getting their meals. The AWW and the other staff also come in time and regularly open the centre*."

[School Teacher, Kushalgarh]

Many teachers reported taking a personal interest in visiting and monitoring the functioning of AWCs; however due to their own busy schedule and excessive workload (in part due to staff shortage), they were not always proactive.

"*We are already overburdened with the work of the school. It is not possible to check the functioning of the AWC."*

[School Teacher, Ghatol]

All the ward Panches (elected Panchayat [local self-government] member) were unaware of the Village Health Sanitation and Nutrition Committees (VHSNC) and played no role in this critical village level platform for promoting health and nutrition of mothers and children.

✶ *Core theme 8: WASH practices can play a vital role*

Caregivers were unanimous in their perception that illnesses such as diarrhea, fever or cough represented one contributing factor of inappropriate nutrition among children. There was little awareness that these diseases may be partially linked to WASH deficiencies. Hand pumps were the only source of drinking water

For peer review only - http://bmjopen.bmj.com/site/about/guidelines.xhtml

in both blocks. Unlike in Ghatol where a majority of hand pumps have water throughout the year, some sources in Kushalgarh dry up "*due to scarcity of water*" [ANM, Kushalgarh] during the summer months. The only measure for drinking water treatment was using a cloth for filtering the water while filling; this practice (as reported by the FHWs) was more common in Kushalgarh than in Ghatol. In several villages of Kushalgarh, a local reverse osmosis plant using solar panels was set up under a new scheme by the Public Health and Engineering department.

Mothers, grandmothers and other key informants reported that communities in Ghatol were more willing to construct sanitary facilities than those in Kushalgarh, even though there is a national sanitation program which incentivizes toilet construction at household level.

"*Those who constructed the toilets have not got their due payments. I have been following up with the Sarpanch's office regularly, but there is no response. This discourages those who have not yet constructed.*"
[Ward Panch, Kushalgarh]

In both blocks, however, a majority of households had access to functional toilets.

"*The government has constructed toilets in every household. Whatever facilities the government has provided us, I have provided in this village.*"
[Ward Panch, Ghatol]

Most members reported using toilets, with the exception of a few elders who prefer open defecation. None of the under-two children defecated in toilets. Small children defecated on pieces of cloth which were subsequently disposed of in open fields near the house. The older children defecated in the open, near their homes, except some in Ghatol who were reported to use toilets.

Awareness on the importance of hand washing was high in all study villages. Hand washing with water and soap before cooking and eating was unanimously reported in Ghatol, but less so in Kushalgarh due to water scarcity.

Discussion

IYCF practices can be categorized as optimal and suboptimal. Optimal practices such as early breastfeeding initiation, reduction in pre-lacteal use, mothers being encouraged to exclusively breastfeed the child (till six

For peer review only - http://bmjopen.bmj.com/site/about/guidelines.xhtml

months) and continued breastfeeding till two years were reported in our study villages. Suboptimal practices were more widespread and included giving water in the first six months, feeding biscuits from the fourth month and complementary feeds lacking in quantity, frequency and diversity. The emergent model bears out the interactions of the socio-ecological elements framed in our conceptual framework (Figure 5).

Figure 5: Emergent Model

It is in this backdrop that we sought to identify factors that influence and shape these existing practices and then design interventions that are able to address barriers and promote improvement. The evidence presented in this paper forms the basis of the socio-culturally appropriate, tailored, integrated and interdisciplinary interventions developed as part of the PANCHSHEEL project drawing on the sectors of: (i) health and nutrition, (ii) education and (iii) engineering and environment.

Most of the facilitators and barriers were in the realm of knowledge and skills. The two most common barriers across most households were lack of appropriate knowledge of mothers and other caregivers about: (i) complementary feeding practices (frequency, quantity and quality) and, (ii) recipe for cooking Take Home Ration (supplementary nutrition) distributed by the Anganwadi Centre. The knowledge gaps also resonated with incomplete information that was provided by the FHWs; this was also one of the barriers to promoting positive IYCF practices. Two such (highly prevalent) practices were giving water to babies less than 6 months and feeding biscuits. We also noted the divergence between perceptions of mothers and grandmothers. This was largely on account of mothers caring and feeding the children more actively in contrast to grandmothers who looked after children largely when mothers were away for work and were not often the final decision makers of care and feeding practices.

Resource constraints for mothers emerged as another significant factor. Lack of mother's time to continue exclusive breastfeeding as well as dedicating time for separate cooking and feeding was one of the commonest barriers to positive IYCF practice. As male members travelled to nearby towns for daily wage labor or migrated to nearby states, (owing to lack of employment opportunities in the villages), women were compelled to dedicate more time to agricultural work. The poor environmental conditions and limited access to basic water and sanitation services also contributed to the time and resource constraints faced by women.

Little or no availability of animal milk resulted in children between 6 months to 2 years hardly receiving any milk in their diet. Limited use of vegetables and fruits was due lack of markets, inadequate resources to cultivate and high cost. As a result, children's diets were seldom rich in iron and vitamins. The absence of adequate resources was not merely a household level issue, but also rooted in the local cultural and

For peer review only - http://bmjopen.bmj.com/site/about/guidelines.xhtml

religious contexts. The remaining two determinant categories; motivation and attitude, were mostly linked to either knowledge or resources.

Delineation of these factors helped in identifying key 'actors' who could bring about improvement in IYCF practices. To ensure continued support to mothers and other caregivers on positive IYCF practices, the knowledge and skills of FHWs, especially ASHA Sahyogini and Anganwadi Workers, need to be upgraded. Finally, there is a need for garnering community support for these children for which the role of important members like school teachers and elected representative was also identified as crucial.

This study was also subjected to certain logistical limitations and research related concerns. The language was a barrier to be addressed, everyone knew Hindi, but only two people in the team were versed with Wagdi, the local dialect. This resulted in challenges for translation and transcription of the data from the discussions. Moreover, the availability of the functionaries and the key stakeholders including the teachers at the schools, local official representatives were limited. This also added to the time constraint. All data collected was based on the observation and interactions held in a limited time of 2 hours per village. Sometimes the recall of information was difficult for participants, this was seen when the exact dates of births could not be retrieved resulting in calculation of the child's age based on estimated information on their month and year of birth. Besides, there were challenges to encourage women to participate in the interviews. They either did not feel empowered to contribute or were busy with household chores. For the same reason, availability of mothers was limited considering their demand for domestic work is a lot more. To encourage them there were multiple attempts to organize FGDs and KIIs. The researchers had to spend a significant amount of time with the caregivers to build the space where some of them could freely and confidently contribute to the discussion. On top of this, the decision to include mothers who have children at the age of less than 2 years old led to a varied number of eligible mothers from 35 households to 68 households in some villages, which was not align with the initial plan to cover 50 households per village. Lastly, there might be a selection bias of the participants of the FGDs due to the active role of AWW and ASHA workers in the selection of mothers which implied that only those with whom they have a good rapport would be identified. The prior training about the process could also influence the information given by some of the mothers. Another reason for the selection bias could also be that most of the FDGs were held in the AWC, as a result of which only the residents near the AWC could take part excluding the ones that are far away. We acknowledged the limitations and aimed to find ways to overcome the challenges we faced.

For peer review only - http://bmjopen.bmj.com/site/about/guidelines.xhtml

This study, and the urgent need for IYCF interventions, still holds a significance in the light of the recently released Global Hunger Index 2019 data [21]. India's Global Hunger Index (GHI) score declined from 38.8 in 2000 (rank: 83 out of 113 countries) to 30.3 in 2019 (rank: 102 out of 117 countries), placing the country in the 'serious' category. Achieving internationally agreed targets on stunting and wasting in under-five children is key to achieving Sustainable Development Goals. The complex set of factors and barriers to appropriate IYCF practices – captured in the HEEE conceptual framework and the qualitative data – pose a challenge in achieving the SDGs. While the Government of India has designed and implemented several programs to address these barriers, they reported limited success primarily because of the nature of isolated interventions as opposed to much needed multifaceted approach. The task at hand is therefore a well-designed, locally feasible, multi-sectoral interventions across health and nutrition, education as well as water and sanitation. Through this study we demonstrate the need for integrated and cross-sectoral research to comprehensively shape the feeding practices of children; thereby creating a scope for coordinating the ongoing interventions in a manner to achieve the desired target (the subject of another paper).

Ethics approval and consent to participate: The PANChSHEEEL Project, including qualitative and quantitative data collection methods as well as the tools obtained ethical approval from the UCL ethics committee (REF NO: 4032/002) in United Kingdom and the Sigma-IRB (REF NO: 10025/IRB/D/17-18 REF NO: 4032/002) in India.

Declaration of Conflicting Interest:

The authors declared no potential conflicts of interest with respect to the research, authorship and/or publication of this paper.

Financial Support and Funding:

This work was also supported by the Global Challenges Research Fund and funded by the MRC, AHRC, BBSRC, ESRC and NERC [grant number: MR/P024114/1].

Author Contributions:

ML, ML, PP, RK, and SS conceived the original concept of the study and designed the research methodology. SR, ML, PP, RK, HC, SS, SPP, TS, HR, and PP carried out the interviews, analysed the data, and wrote the paper. ML, ML, PP, SR, HC, NS, SS, SPP, LB, VV, PD, HR and RK validate the study and

For peer review only - http://bmjopen.bmj.com/site/about/guidelines.xhtml

For peer review only

revised the manuscript critically for important intellectual content. SA contributed to the manuscript writing, edited the final manuscript and prepared for submission. ML had primary responsibility for the final content. All authors read and contributed to the reviewing the analysis of the data, the designing of the manuscript, and the approval of the final manuscript.

Data Statement:

The data of this study is available from the corresponding author upon reasonable request.

For peer review only

For peer review only - http://bmjopen.bmj.com/site/about/guidelines.xhtml

References

1. Fanzo J, Hawkes C, Udomkesmalee E, et al. (2018) Global Nutrition Report: Shining a light to spur action on nutrition.
2. UNICEF-WHO-World Bank: Joint Child Malnutrition Estimates 2018. Available From: https://data.unicef.org/resources/levels-and-trends-in-child-malnutrition-2018/
3. Niti Aayog. (2017). National Nutrition Strategy. New Delhi; India: Niti Ayog, Government of India. Available from: http://niti.gov.in/writereaddata/files/document_publication/Nutrition_Strategy_Booklet.pdf.
4. UNICEF. (2017). Forest Lanterns. New Delhi: Penguin Random House.
5. World Health Organization. Infant and young child feeding: model chapter for textbooks for medical students and allied health professionals. Available from: https://www.who.int/nutrition/publications/infantfeeding/9789241597494.pdf
6. Jones AD, Ickes SB, Smith LE, et al. (2013) World Health Organization infant and young child feeding indicators and their associations with child growth: a synthesis of recent findings.
7. Gupta A, Thakur N. Infant and young feeding practices in india: current status and progress towards SDG targets. Proceedings of the Indian National Science Academy. (2018) Nov 13;84(4):853-65.
8. UNICEF. (2019). Global UNICEF Global Databases: Infant and Young Child Feeding: Minimum acceptable diet, Minimum diet diversity, Minimum meal frequency. New York; USA: UNICEF. Available from: https://www.unicef.org/nutrition/files/IYCF_programming_guide_Apr_2013.pdf
9. International Institute of Population Sciences. (2017). National Family Health Survey, India: Key Findings from NFHS-5(2019-21). Mumbai, India: International Institute of Population Sciences

10.International Institute of Population Sciences. (2017). National Family Health Survey, India: Key Findings from NFHS-4 (2015-16). Mumbai, India: International Institute of Population Sciences. Available from: https://dhsprogram.com/pubs/pdf/FR339/FR339.pdf

11.Imran M, Ilyas N, Mahmood T. Infant and young child feeding (IYCF) practices. Rawal Medical Journal. 2017 Oct 1;42(4):546-9.

12.Chaturvedi S, Ramji S, Arora NK, Rewal S, Dasgupta R, Deshmukh V. Time-constrained mother and expanding market: emerging model of under-nutrition in India. BMC public health. 2016 Dec;16(1):632.

For peer review only - http://bmjopen.bmj.com/site/about/guidelines.xhtml

13.Dasgupta R, Arora NK, Ramji S, Chaturvedi S, Rewal S, Suresh K, Deshmukh V, Thakur N. Managing Childhood Under-Nutrition: Role and Scope of Health Services. Economic and Political Weekly. 2012 Apr 21:15-9.

14. Ramji S. Impact of infant & young child feeding & caring practices on nutritional status & health. Indian J Med Res. 2009 Nov 1;130(5):624-6.

15. Desai S, Banerji M. Negotiated identities: Male migration and left-behind wives in India. Journal of Population Research. 2008 Oct 1;25(3):337-55.

16. Lee C, Lakhanpaul M, Maza Stern B, Parikh P. Associations between the household environment and stunted child growth in rural India: A cross-sectional analysis. UCL Open: Environment. 2019 Mar 6.

17. Modugu, H.R., Khanna, R., Dash, A. *et al.* Influence of gender and parental migration on IYCF practices in 6–23-month-old tribal children in Banswara district, India: findings from the cross-sectional PANChSHEEEL study. *BMC Nutr* **8,** 10 (2022)

18. Parikh, P., Kwami, C. S., et al. Linkages between environmental factors (WASH and energy) and Infant and Young Child Feeding practices in rural India: implications for cross-sectoral interventions for child health. Journal of Water, Sanitation and Hygiene for Development.(2021)

19. Saunders B, Sim J, Kingstone T, Baker S, Waterfield J, Bartlam B, Burroughs H, Jinks C. Saturation in qualitative research: exploring its conceptualization and operationalization. Quality & quantity. 2018 Jul 1;52(4):1893-907.

20. Halcomb, E.J. and Hickman, L., 2015. Mixed methods research.

21. Welt Hunger Hilfe. (2019). Global Hunger Index: The Challenge of Hunger and Climate Change. Dublin, Ireland: Welt Hunger Hilfe. Available from: https://www.globalhungerindex.org/pdf/en/2019.pdf

For peer review only

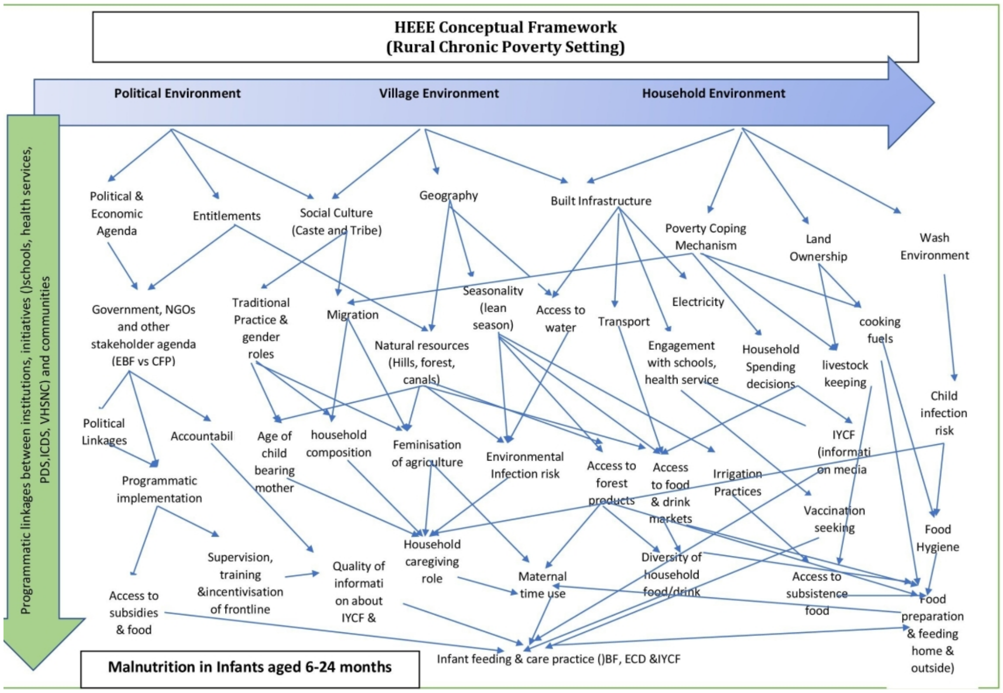

Figure 1: HEEE Conceptual Framework

297x209mm (200 x 200 DPI)

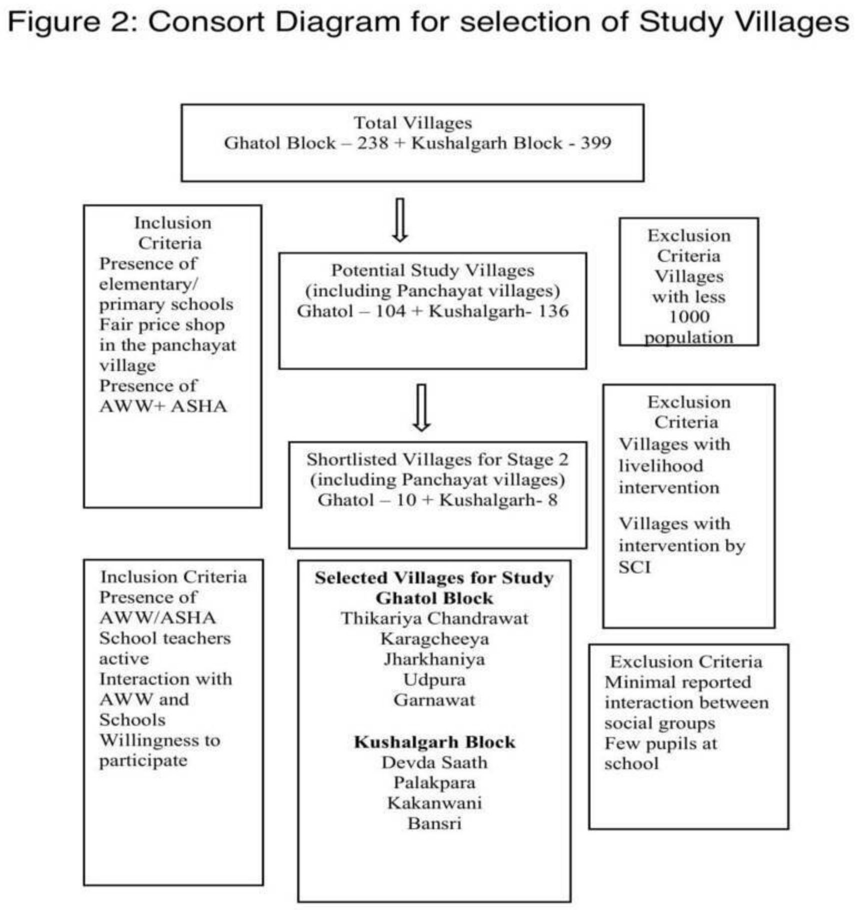

Consort Diagram for selection of Study Villages

76x76mm (300 x 300 DPI)

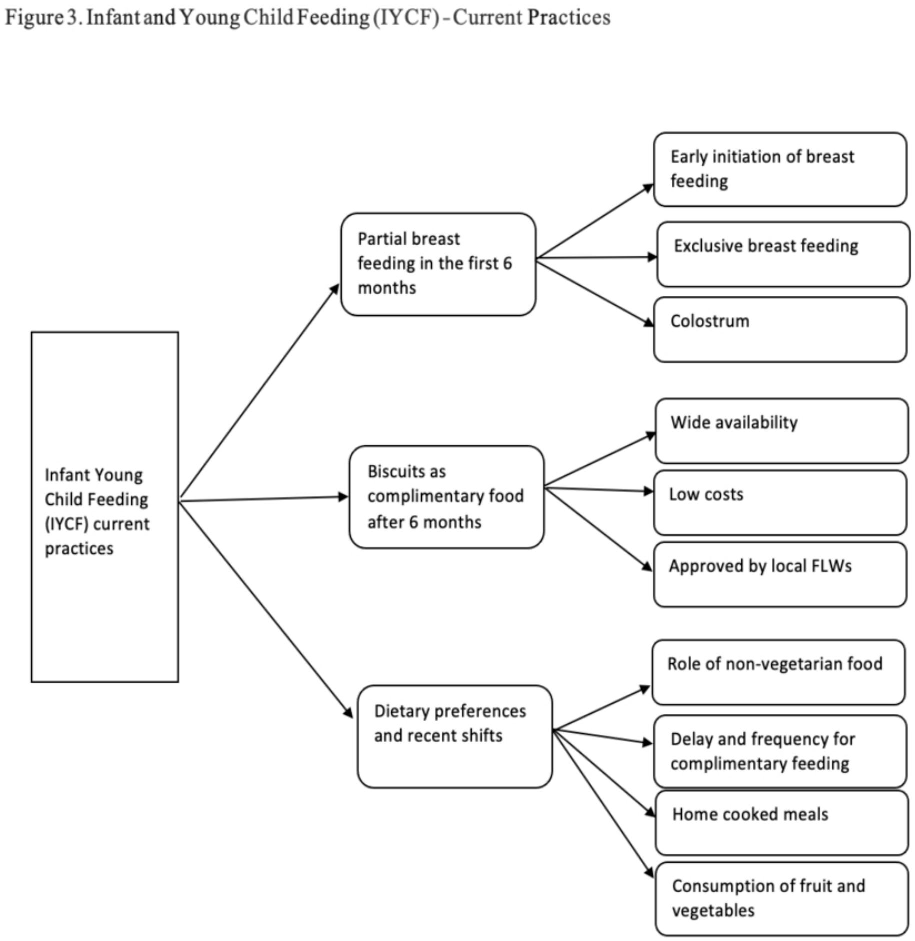

Infant and Young Child Feeding (IYCF) – Current Practices

193x193mm (118 x 118 DPI)

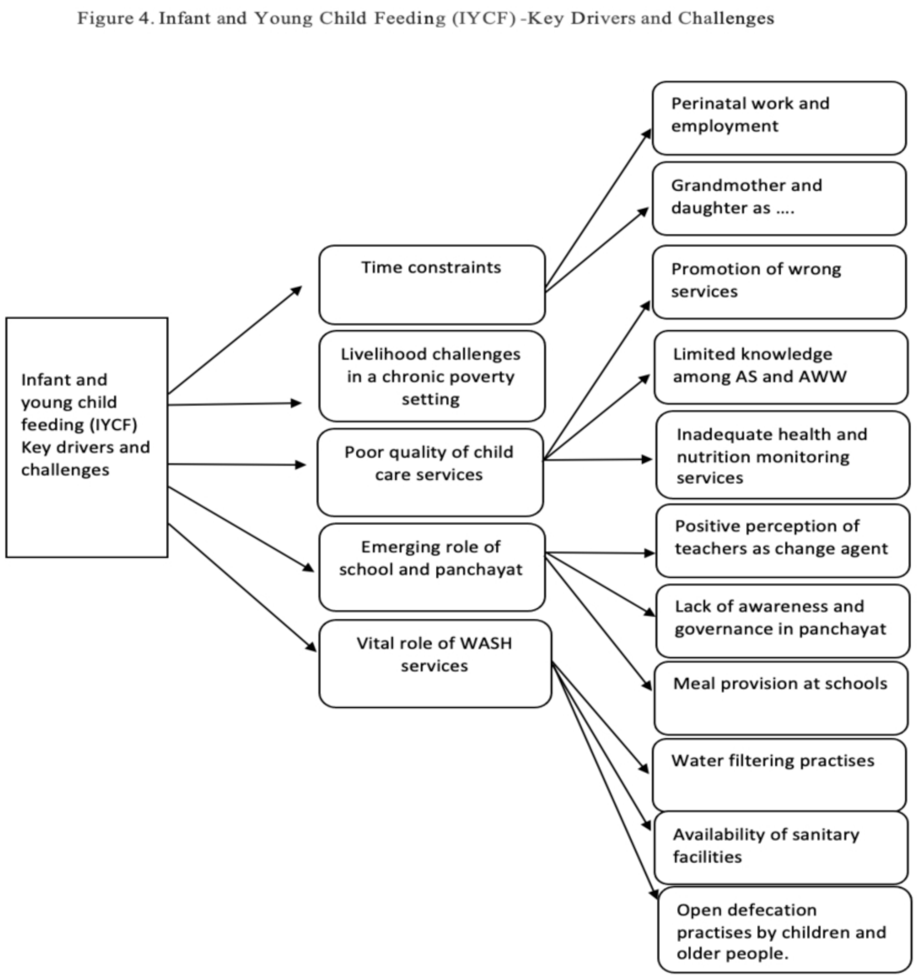

Infant and Young Child Feeding (IYCF) – Key Drivers and Challenges: Connecting subthemes

193x193mm (118 x 118 DPI)

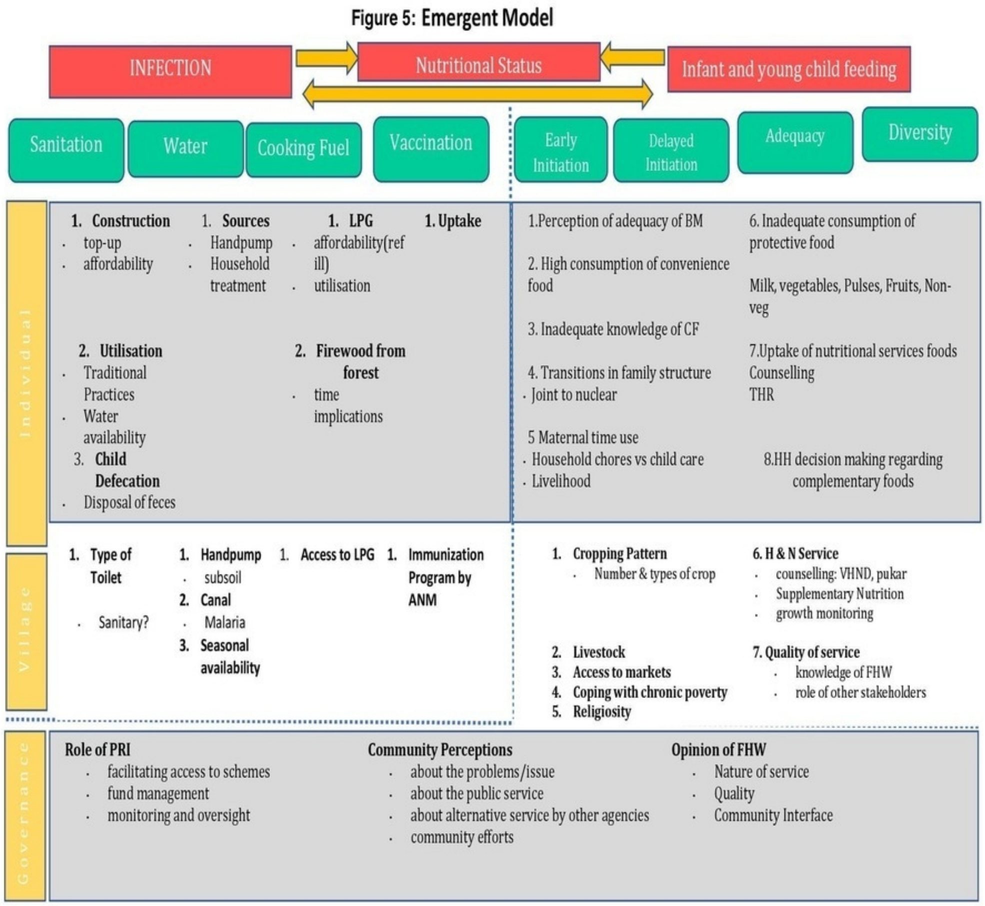

Emergent Model

76x76mm (300 x 300 DPI)

Supplementary Table

Trends in IYCF indicators in India (in %)

	States	Children receiving solid or semi-solid food and breast milk (%)		Trends in IYCF (%)
		NFHS 3 (2005 – 2006)	NFHS 4 (2015 – 2016)	
**India**		52.6	42.7	-9.9
**North**	Rajasthan	38.7	30.1	-8.6
	Punjab	50.9	41.1	-9.8
	Uttar Pradesh	41.2	32.6	-8.6
**North East**	Sikkim	85.4	61.8	-23.6
	Manipur	77.4	78.38	1.4
	Arunachal Pradesh	80.2	53.6	-26.6
**West**	Gujarat	54.1	49.4	-4.7
	Maharashtra	45.5	43.3	-2.2
**Central**	Madhya Pradesh	46	38.1	-7.9
	Chhattisgarh	49	53.8	4.8
**East**	Bihar	54.5	30.7	-23.8
	Jharkhand	60.2	47.2	-13
	West Bengal	47.1	52	4.9
	Odisha	65.4	54.9	-10.5
**South**	Karnataka	69.7	46	-23.7
	Tamil Nadu	81.2	67.5	-13.7
	Kerala	93.9	63.1	-30.8

Source: Dasgupta, Chaand and Rakshit (2018) [19]

For peer review only

Core Theme	Sub-theme	Participant type	FDG/KII	Example Quote
**Infant and Young Child Feeding (IYCF) – Current Practices**	*Partial breastfeeding in the first 6 months*	Mothers, Kushalgarh	FDG	*"When the child starts crying after birth then breast milk is produced and the mother is ready to breastfeed the child."*
		Mothers, Ghatol	FDG	*"No, we do not breastfeed the child soon after childbirth. Breastfeeding depends on the mother's ability to produce milk. Breastfeeding begins only after midnight if a birth occurs during the evening.*
		AS, Kushalgarh	KII	*"If the baby is delivered at home breastfeeding is starts immediately after birth. Most cases I am present during the childbirth and hence I ensure that breastfeeding starts within an hour for cases that I attend."*
		Grandmothers, Kushalgarh	FGD	*"Only when the mother's milk secretion does not start, goat's milk is given. Honey or any water is not given."*
		Mothers, Ghatol	FGD	*"Mothers of children who are on breast milk work inside the village, so they can be summoned home whenever the child cries"*
		AWW, Ghatol	KII	*"In the summer season the child becomes thirsty, so water is given to the child. We also recommend giving water sometimes."*
		Grandmothers, Kushalgarh	FDG	*"When the child's mother is out of the village due to some work or is working in the field, goat milk is given when the child cries."*
	Biscuits as complementary food after 6 months	Grandmothers, Kushalgarh	FGD	*"Children like biscuits and it is easy to feed them. We give them one biscuit and they keep eating it. We do not have to feed them."*
		Mothers, Ghatol	FGD	*"This is the cheapest option and is easily available in the village."*
		ANM, Kushalgarh	KII	*"We tell the mothers that they can give biscuits to the child, at least it will keep them full."*
		Mothers, Ghatol	FDG	*"The baby starts drinking milk (dairy) after 6 months. We also give rab (grounded maize porridge), biscuits dipped in the water and sometime daal ka pani (lentil soup). We know what to give and what not to. The AWW also tells us about this. We only take care of all things related to the child like cooking and feeding."*

For peer review only

	Dietary preferences and recent shifts	Mothers FGD, Kushalgarh	FGD	*"We feed them separately. We do not feed them with us. Whenever the children get hungry we feed them. They do not understand anything; they eat whatever we give them."*
		Ward Panch, Ghatol	KII	*"Nobody eats non-vegetarian food as they have become 'followers'. This is a recent trend. Hawan (worshipping with holy fire) was conducted in the village after which every family has stopped eating. No question of giving it to children."*
		Teacher, Kushalgarh	KII	*"Very few families eat non-vegetarian (foods). Those who eat also give their children. This is made only if there is a guest at home or there is any occasion. Chicken is mostly eaten (on such occasions). Eggs are not eaten as they are left to hatch and the goats are sold."*
		Teacher, Kushalgarh	KII	*"Fruits are not very common in daily diet. Only when the parents go to the market they get fruits. There is availability of local fruits which are not too good for children, like tamarind and plum."*
		ANM, Ghatol	KII	*"Some vegetables are grown in the backyard of every home, mostly beans and brinjal. This is used quite regularly. Potatoes, tomatoes and onion are cooked almost daily. The local vegetables like spinach are seldom grown as people do not want to eat them."*
	Time Constraints	Grandmother, Kushalgarh	FDG	*"The mother cannot sit at home taking care of the child. Who will look after the animals, get water or cook? They also have to do agricultural work."*
		ANM, Ghatol	KII	*"Some mothers work a lot and hence are not able to take care of the child adequately. Grandmothers help, but they are also old people and cannot do much."*
		Grandmother, Ghatol	FDG	*When my daughter-in-law works outside, then we grandmothers take care of the children. Like when the child is crying and have to be fed; we keep them in our laps and make them comfortable. We keep an eye on the children and their activities."*
		School Teacher, Kushalgarh	KII	*"Most people here are in agriculture. Both males and females of all families take part in agriculture. Men play an important role of sowing and harvesting while women do the rest. Women are engaged all year round."*
		Ward Panch, Ghatol	KII	*"Most houses keep goats, some also have cows. Buffaloes are very less in the village. All of these animals do not give milk. Milk from goat is used for consumption at home. Cow*

For peer review only

**Infant and Young Child Feeding (IYCF) – Key Drivers and Challenges**	*Livelihood challenges in a chronic poverty setting*			*and buffalo milk is used for making ghee. Few families also sell it. They do not feed milk to their children"*
		Mothers, Ghatol	FDG	*Taking care of the animals is the work of women as they stay at home. They are the ones who clean the shed and give the animals fodder and water."*
		School Teacher, Ghatol	KII	*"Some men from the village work in Mayur mills (textile mill near Banswara). Some also work as wage labour, but only during lean seasons. Women with small children do not go out of the village to work."*
	Poor Quality of Childcare services	AWW, Kushalgarh	KII	*"Neither LS (Lady Supervisor) or the CDPO (Child Development Project Officer) visits our centre. We only go to the sector meetings where they ask us about the number of pregnant women registered, children born and packets of THR distributed. They sometime give us new information. The focus is on children coming to AWC."*
		AWW, Ghatol	KII	*"We take the weight of the child who comes for the vaccination. Sometimes the mothers do not get the card when they come for vaccination, so weight cannot be recorded in the (Mamta) card."*
		AWW, Kushalgarh	KII	*"We take weight, but currently the weighing machine is not working."*
	Emerging Role of Schools and Panchayats	School Teacher, Kushalgarh	KII	*"The AWC is in the school premises; we can always keep a check whether children are getting their meals. The AWW and the other staff also come in time and regularly open the centre."*
		School Teacher, Ghatol	KII	*"We are already overburdened with the work of the school. It is not possible to check the functioning of the AWC. However sometimes while going back from school I go to the AWC and speak to the workers. I have seen their records and gave them suggestions to maintain it better. What more can we do?"*
		AWW, Ghatol	KII	*"Toilets have been constructed in every household but not everyone is using it. Elder members prefer open defecation."*
		ANM, Kushalgarh	KII	*"Few families have constructed the toilets and very few members use it due to scarcity of water."*

For peer review only

	Vital role of WASH practices	Ward Panch, Kushalgarh	KII	*"Those who constructed the toilets have not got their due payments. I have been following up with the Sarpanch's office regularly, but there is no response. This discourages those who have not yet constructed."*
		Ward Panch, Ghatol	KII	*"The government has constructed toilets in every household. Whatever facilities the government has provided us, I have provided in this village. Half of the villagers use toilets. There is a delay in payment, but the Sarpanch has told me that it will be soon released."*

For peer review only

COREQ (COnsolidated criteria for REporting Qualitative research) Checklist

A checklist of items that should be included in reports of qualitative research. You must report the page number in your manuscript where you consider each of the items listed in this checklist. If you have not included this information, either revise your manuscript accordingly before submitting or note N/A.

Topic	Item No.	Guide Questions/Description	Reported on Page No.
**Domain 1: Research team and reflexivity**			
Personal characteristics			
Interviewer/facilitator	1	Which author/s conducted the interview or focus group?	
Credentials	2	What were the researcher's credentials? E.g. PhD, MD	
Occupation	3	What was their occupation at the time of the study?	
Gender	4	Was the researcher male or female?	
Experience and training	5	What experience or training did the researcher have?	
Relationship with participants			
Relationship established	6	Was a relationship established prior to study commencement?	
Participant knowledge of the interviewer	7	What did the participants know about the researcher? e.g. personal goals, reasons for doing the research	
Interviewer characteristics	8	What characteristics were reported about the inter viewer/facilitator? e.g. Bias, assumptions, reasons and interests in the research topic	
**Domain 2: Study design**			
Theoretical framework			
Methodological orientation and Theory	9	What methodological orientation was stated to underpin the study? e.g. grounded theory, discourse analysis, ethnography, phenomenology, content analysis	
Participant selection			
Sampling	10	How were participants selected? e.g. purposive, convenience, consecutive, snowball	
Method of approach	11	How were participants approached? e.g. face-to-face, telephone, mail, email	
Sample size	12	How many participants were in the study?	
Non-participation	13	How many people refused to participate or dropped out? Reasons?	
Setting			
Setting of data collection	14	Where was the data collected? e.g. home, clinic, workplace	
Presence of non-participants	15	Was anyone else present besides the participants and researchers?	
Description of sample	16	What are the important characteristics of the sample? e.g. demographic data, date	
Data collection			
Interview guide	17	Were questions, prompts, guides provided by the authors? Was it pilot tested?	
Repeat interviews	18	Were repeat inter views carried out? If yes, how many?	
Audio/visual recording	19	Did the research use audio or visual recording to collect the data?	
Field notes	20	Were field notes made during and/or after the inter view or focus group?	
Duration	21	What was the duration of the inter views or focus group?	
Data saturation	22	Was data saturation discussed?	
Transcripts returned	23	Were transcripts returned to participants for comment and/or	

For peer review only

Topic	Item No.	Guide Questions/Description	Reported on Page No.
		correction?	
**Domain 3: analysis and findings**			
Data analysis			
Number of data coders	24	How many data coders coded the data?	
Description of the coding tree	25	Did authors provide a description of the coding tree?	
Derivation of themes	26	Were themes identified in advance or derived from the data?	
Software	27	What software, if applicable, was used to manage the data?	
Participant checking	28	Did participants provide feedback on the findings?	
Reporting			
Quotations presented	29	Were participant quotations presented to illustrate the themes/findings? Was each quotation identified? e.g. participant number	
Data and findings consistent	30	Was there consistency between the data presented and the findings?	
Clarity of major themes	31	Were major themes clearly presented in the findings?	
Clarity of minor themes	32	Is there a description of diverse cases or discussion of minor themes?	

Developed from: Tong A, Sainsbury P, Craig J. Consolidated criteria for reporting qualitative research (COREQ): a 32-item checklist for interviews and focus groups. *International Journal for Quality in Health Care*. 2007. Volume 19, Number 6: pp. 349 – 357

Once you have completed this checklist, please save a copy and upload it as part of your submission. DO NOT include this checklist as part of the main manuscript document. It must be uploaded as a separate file.

For peer review only
